# *N*,*N′*‐Ethylene‐Bridged Bis‐2‐Aryl‐Pyrrolinium Cations to *E*‐Diaminoalkenes: Non‐Identical Stepwise Reversible Double‐Redox Coupled Bond Activation Reactions

**DOI:** 10.1002/chem.202000255

**Published:** 2020-03-09

**Authors:** Mithilesh Kumar Nayak, Jessica Stubbe, Nicolás I. Neuman, Ramakirushnan Suriya Narayanan, Sandipan Maji, Carola Schulzke, Vadapalli Chandrasekhar, Biprajit Sarkar, Anukul Jana

**Affiliations:** ^1^ Tata Institute of Fundamental Research Hyderabad Gopanpally Hyderabad 500107 India; ^2^ Institut für Chemie und Biochemie Anorganische Chemie Freie Universität Berlin Fabeckstraße 34–36 14195 Berlin Germany; ^3^ Instituto de Desarrollo Tecnológico para la Industria Química CCT Santa Fe CONICET-UNL Colectora Ruta Nacional 168, Km 472, Paraje El Pozo 3000 Santa Fe Argentina; ^4^ Institut für Biochemie Universität Greifswald Felix-Hausdorff-Straße 4 17487 Greifswald Germany; ^5^ Department of Chemistry Indian Institute of Technology Kanpur Kanpur 208016 India; ^6^ Institut für Anorganische Chemie Lehrstuhl für Anorganische Koordinationschemie Universität Stuttgart Pfaffenwaldring 55 70569 Stuttgart Germany

**Keywords:** carbocations, electrochemistry, non-identical reversible reaction, radical reactions, redox chemistry

## Abstract

This work presents a stepwise reversible two‐electron transfer induced hydrogen shift leading to the conversion of a bis‐pyrrolinium cation to an *E*‐diaminoalkene and vice versa. Remarkably, the forward and the reverse reaction, which are both reversible, follow two completely different reaction pathways. Establishing such unprecedented property in this type of processes was possible by developing a novel synthetic route towards the starting dication. All intermediates involved in both the forward and the backward reactions were comprehensively characterized by a combination of spectroscopic, crystallographic, electrochemical, spectroelectrochemical, and theoretical methods. The presented synthetic route opens up new possibilities for the generation of multi‐pyrrolinium cation scaffold‐based organic redox systems, which constitute decidedly sought‐after molecules in contemporary chemistry.

## Graphical abstract

***N***,***N′*** **‐Ethylene bridged** bis‐2‐aryl pyrrolinium cations are reported in this work, which (a) undergo stepwise reversible two‐electron reduction coupled two hydrogen transfer to form *E*‐diaminoalkenes; and (b) have non identical pathways for reduction and oxidation.

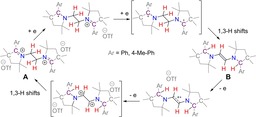

## Introduction

Well‐defined, redox‐induced bond‐breaking and bond‐making processes are at the heart of a huge variety of chemical transformations, including important examples in bio‐ and homogeneous catalysis.^1^ Many such catalytic reactions, particularly in biological systems, function through the simultaneous or stepwise transfer of multiple electrons and protons.^2^ In cases where the redox‐induced chemical transformations are reversible, redox switchable or bistable materials can be devised. The observation and investigation of multiple electron and proton transfer steps have traditionally been the realm of transition metal complexes.^3^ This is because the frontier orbitals of transition metal complexes are readily tunable and accessible for reversible electron transfer. However, there are some recent examples in contemporary chemistry based on *p*‐block elements which provide an alternative to the traditional paradigm of transition metal chemistry.^4^ In this regard, *N*‐heterocyclic carbenes (NHCs)^5^ and cyclic(alkyl)(amino)carbenes (CAACs)^6^ have been established as compounds of particular interest.

**Scheme 1:**
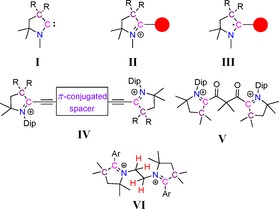
Schematic representation of CAAC‐scaffolds containing mono‐ and dications as redox‐active compounds.

**Figure 1:**
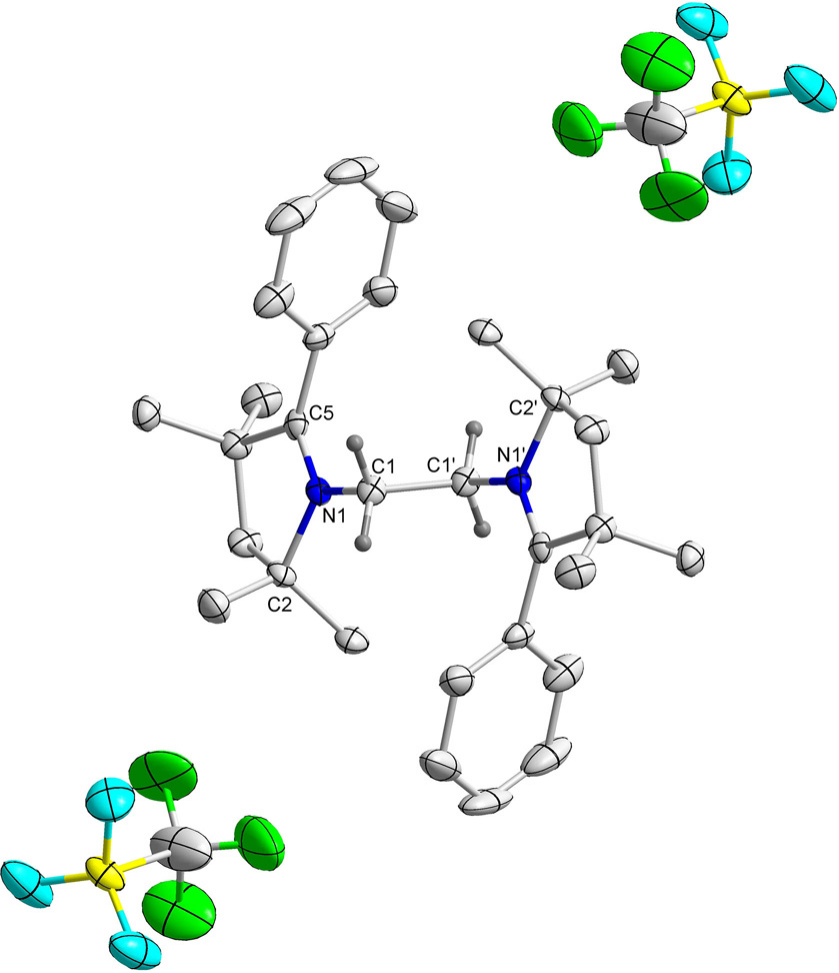
Molecular structure of **3^H^** with thermal ellipsoids at 50 % probability level. All hydrogen atoms except at C1 and C1′ have been omitted for clarity. Selected bond lengths (Å) and bond angles (°): C1−C1′ 1.522(5), N1−C1 1.481(3), N1−C2 1.522(3), N1−C5 1.283(4); C5‐N1‐C2 114.0(2), C5‐N1‐C1 124.8(2), C2‐N1‐C1 121.2(2), N1‐C1‐C1′ 109.8(3).

In recent years, CAAC‐scaffolds were found to display pivotal roles in stabilizing different types of open‐shell compounds.^7^ In these cases, CAACs, **I**, have been used to first synthesize the corresponding pyrrolinium salts, **II**, which are converted to carbon‐based radicals, **III**, through a one‐electron reduction (Scheme 1). Recently, we have disclosed a new synthetic strategy for 2‐substituted pyrrolinium salts without employing CAAC as precursors.^8^ The *C*‐2 center of the 2‐substituted pyrrolinium cation is redox active and therefore the synthesis of a compound with multiple 2‐substituted pyrrolinium cation moieties, consequently, envisioned to result in a molecule with multiple redox centers. Recently, CAAC‐derived bis‐pyrrolinium cations, **IV** 9 and **V** 10 have been reported in the context of mixed‐valent and redox bistable organic compounds, respectively (Figure 1), in which the redox‐active *C*‐2 centers of two pyrrolinium cations are connected by a spacer.

In the present study, *N*,*N′*‐ethylene‐bridged bis‐2‐aryl‐pyrrolinium dications **VI** were synthesized, where two redox‐active moieties were connected by their redox inactive *N*‐centers (Figure 1). In compound **VI** two strong electron‐accepting groups are strategically connected through an ethylene bridge, and which are capable of undergoing C−H bond activation. Furthermore, these systems participate in reversible multiple electron transfer and concomitant bond activation steps. Most notably, it was found that the forward and the backward two‐electron processes follow non‐identical pathways along with multiple C−H bond activation events. It should be mentioned here that transition metal complexes which participate in reversible redox‐induced bond activation reactions (electrochemical‐chemical, EC and electrochemical‐chemical‐electrochemical, ECE mechanisms) often do so by following identical reaction sequences for the forward and reverse transformations.^11^ Even in the case of proton‐coupled electron transfer, PCET reactions the pathway for forward and backward steps are alike.^12^

## Results and Discussion

**Scheme 2:**
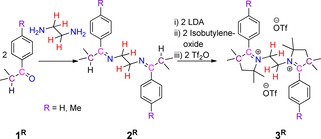
Synthesis of *N*,*N′*‐ethylene bridged bis‐2‐aryl pyrrolinium salts **3^R^**.

**Figure 2:**
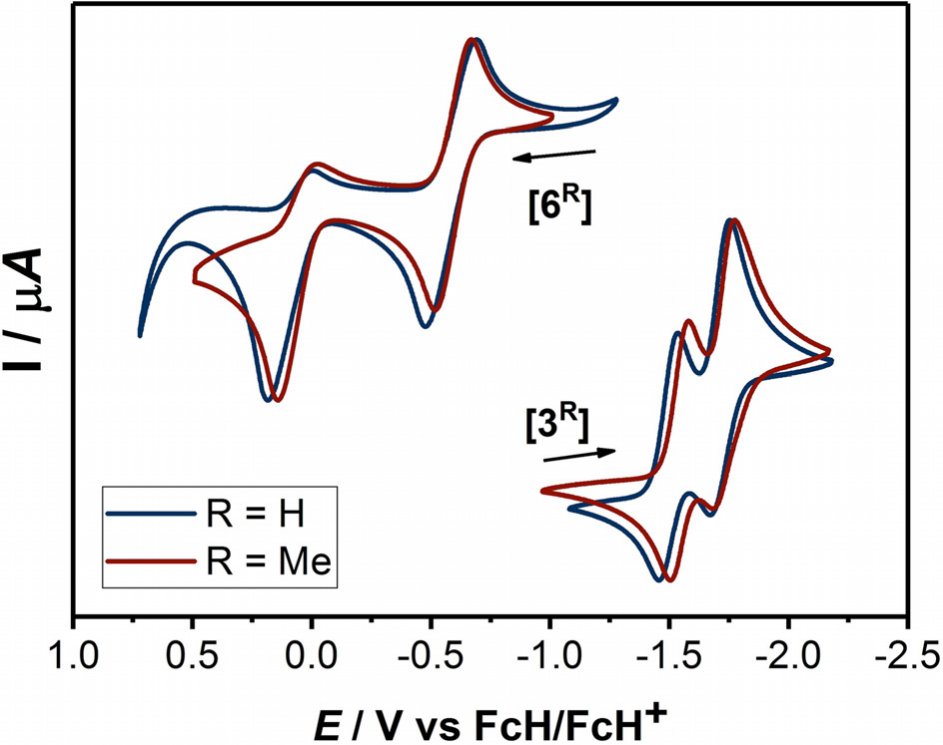
Cyclic voltammograms of **3^R^** in MeCN and 6^R^ in THF at 100 mV s^−1^ with 0.1 m Bu_4_NPF_6_.

In order to obtain the target systems containing two redox‐active centers with a CAAC scaffold, we prepared *N*,*N′*‐ethylene‐bridged bis‐2‐aryl pyrrolinium cations **3^R^**, through the reaction of ethylenediamine with aryl‐isopropyl‐ketone **1^R^**, to get first *N*,*N′*‐ethylene‐bridged bis‐imine **2^R^** (Scheme 2).^13^ **2^R^** were characterized by solution state ^1^H and ^13^C{^1^H} NMR spectroscopy as well as by single‐crystal X‐ray diffraction analysis (see Figures S1 and S2 in Supporting Information). Subsequent sequential one‐pot reactions of **2^R^** with freshly prepared lithium diisopropylamide (LDA), isobutylene oxide, and triflic anhydride afforded *N*,*N′*‐ethylene‐bridged bis‐2‐aryl pyrrolinium cations, **3^R^**, with triflate counter anions, as white crystalline solids (Scheme 2).^13^ ^1^H NMR spectra of compounds **3^H^** and **3^Me^** show singlets at *δ*=3.71 and 3.67 ppm for the C*H*_2_ resonance of the bridging ethylene moiety supporting the expected presence of *C*2‐symmetry. The symmetry was further and unambiguously confirmed by the solid‐state molecular structures of **3^H^** and **3^Me^** (Figure 2 and Figure S3 in Supporting Information).

The cyclic voltammograms of compounds **3^H^** and **3^Me^** exhibit two reduction waves (for **3^H^** at *E*_1/2_=−1.50 and −1.71 V and for **3^Me^** at *E*_1/2_=−1.54 and −1.73 V) in CH_3_CN/0.1 M NBu_4_PF_6_ (Figure 2).^13^ The influence of the different substituents on the redox potentials of the two compounds is marginal. Both reduction waves appear reversible on the cyclic voltammetry timescale. However, spectroelectrochemical measurements showed that only the first reduction step for both compounds is reversible in an electrochemical sense (vide infra). In addition, a third reduction at more negative potentials was observed. This reduction step is irreversible for both compounds and was not probed further.

**Figure 3:**
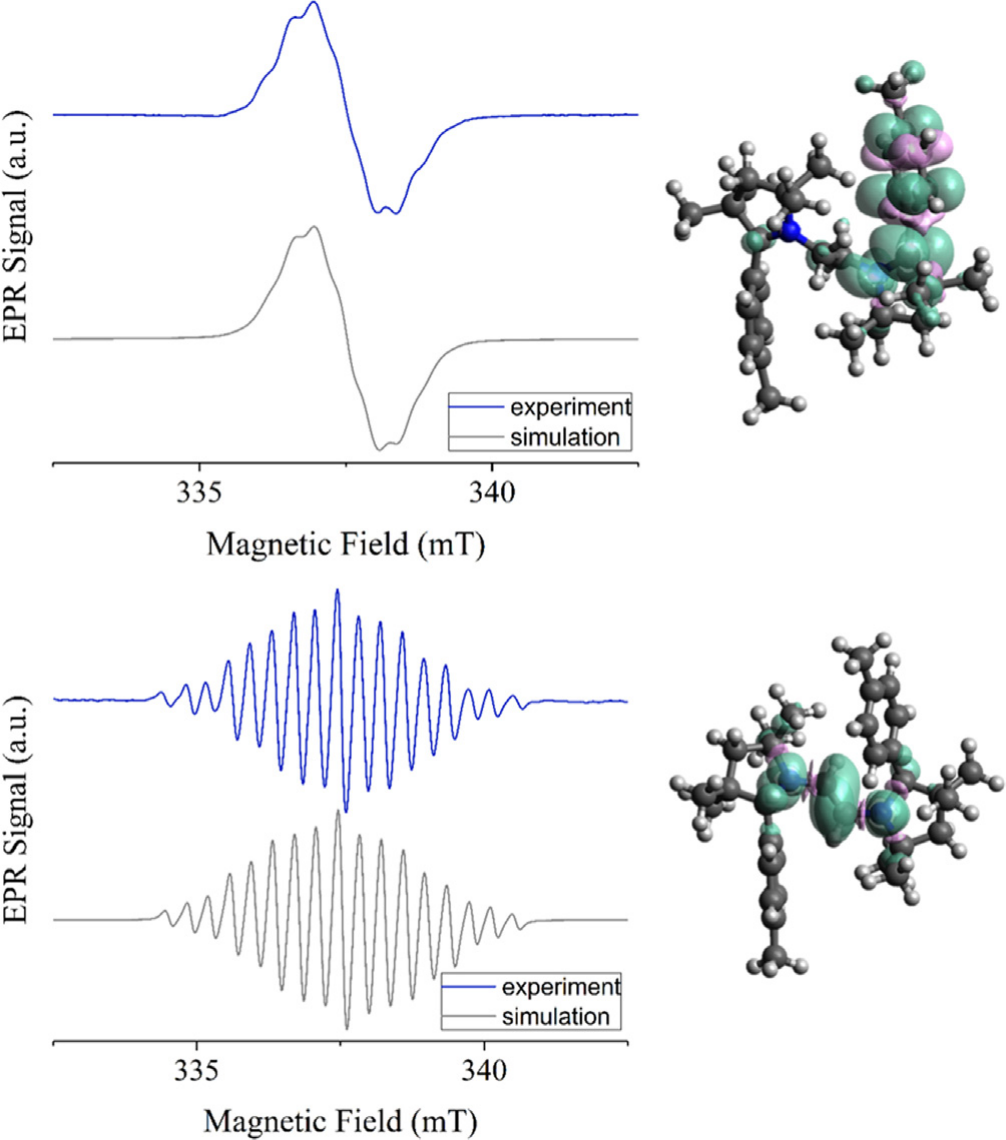
Left: Experimental (blue) and simulated (grey) EPR spectra of electrochemically generated **4^Me^** (top) and **7^Me^** (bottom). Right: calculated spin densities for **4^Me^** (top) and **7^Me^** (bottom).

**Scheme 3:**
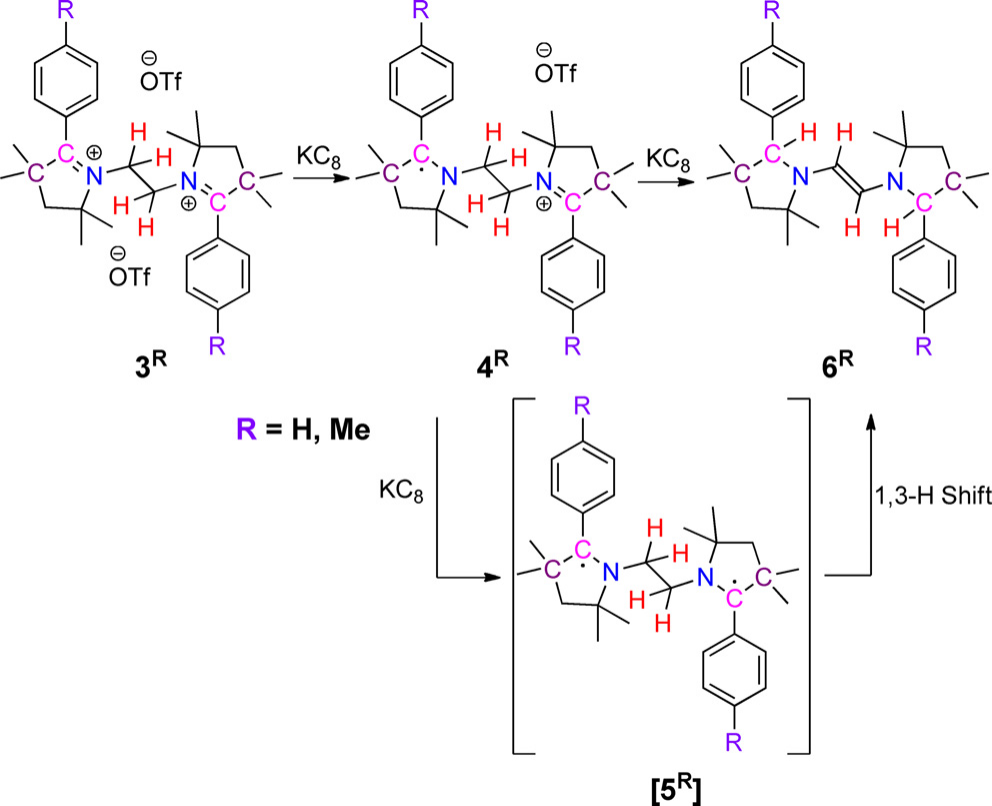
Synthesis of *E*‐diaminoalkenes **6^R^** by sequential reductions and intramolecular H‐abstraction of **3^R^**.

Compound **3^H^** displays absorption bands at 215 and 267 nm while those of **3^Me^** appear at 217 and 283 nm in their respective UV/Vis spectra. These wavelengths are in accordance with the compounds’ colorless nature. TD‐DFT calculations on **3^H^** nicely reproduce the experimental spectrum (Figure S36, Table S4 in Supporting Information). Upon one‐electron reductions to compounds **4^H^** and **4^Me^** new bands appear in the visible region at 346 and 478 nm and at 347 and 492 nm, respectively (Figures S12 and S13 in Supporting Information). TD‐DFT calculations confirm the origin of these bands from radical‐containing species (Figure S36, S40–42 and Table S4 in Supporting Information). No other bands were observed beyond 500 nm for the one‐electron reduced forms of either compound. This fact likely points to a class I nature of these formally mixed‐valent compounds according to the Robin–Day classification,^14^ as would be expected for two redox‐active units connected via a non‐conjugated ethylene bridge moiety.^15^ Corroborating the radical nature of these species, **4^H^** and **4^Me^** display featureless isotropic electron paramagnetic resonance, EPR spectra at 295 K in fluid solution with *g*‐values of 2.0028 and 2.0027 (Figure 3). At 223 K, however, the spectra started to show partial resolution of hyperfine splittings. These features could be simulated by considering a hyperfine coupling of the electron spin with the adjacent ^14^N nucleus (A_N_=11.95 MHz) and six ^1^H nuclei of the CMe_2_ moiety with splittings ranging from 5 to 10 MHz for **4^H^**, and similar values for **4^Me^**. Spin density calculations at the PBE0/def2‐TZVP level of theory showed that the spin is largely located on one side of the molecule (Figure S31 in Supporting Information), thus also indirectly corroborating the class I nature of these mixed‐valent compounds. In the absence of a crystal structure, UV/Vis/EPR spectroelectrochemistry provides unequivocal evidence for the proposed chemical and electronic structures of the one‐electron reduced species **4^H^** and **4^Me^**, and proves that no H‐atom abstraction takes place in the one‐electron reduced species. The second reduction step turned out to be irreversible on the spectroelectrochemical timescale for both compounds and we were not able to generate spectra that can be readily assigned to any particular species. Due to singlet–triplet population equilibria and an increased number of resonances. A spin‐coupled dinuclear EPR species has an intrinsically weaker signal than a mononuclear species at equal concentration. Furthermore, if the dinuclear species takes on different conformations in solution, the spectra could be further broadened, as both exchange and magnetic dipolar interactions heavily depend on interspin distance. Thus, it is much harder to detect a spin‐coupled species than it is to observe an *S=*1/2 species during an EPR SEC experiment, particularly if the spin‐coupled species is unstable. This fact supports a double H‐atom transfer reaction from the initially formed unstable diradical, that is, [**5^H^**] and [**5^Me^**] species (Scheme 3). However, the H‐atom shifts most probably occurs at a timescale that is much longer than the spectroelectrochemical timescales.

In order to isolate the one‐electron as well as the two‐electron reduced products, compounds **3^H^** and **3^Me^** were reacted with 1 and 2 equiv of KC_8_ in THF (Scheme 3).^13^ From the 1:1 reaction of **3^H^**/**3^Me^** and KC_8_ it was not possible to isolate the corresponding radical cation, **4^R^**. However, the formation of the radicals in solution was unequivocally confirmed by solution state EPR spectroscopy (vide supra).

The 1:2 reactions of **3^H^**/**3^Me^** and KC_8_ led to the formation of *E*‐diaminoalkenes **6^H^** and **6^Me^**, in 65 and 73 % yields, respectively (Scheme 3). During the reaction, apart from the one‐electron reduction at each pyrrolinium moiety, two H‐atom shifts from the *N*,*N′*‐ethylene‐bridge take place. Therefore, the isolation of **6^H^**/**6^Me^** after two‐electron reduction of **3^H^**/**3^Me^**, indicates that the reaction proceeds through the formation of a diradical, [**5^H^**]/[**5^Me^**], followed by a double H‐atom shift from the ethylene moiety to the C‐atom of the heterocycle. Such kind of intramolecular hydrogen atom migration has precedence in the case of alkyl radicals.^16^ This procedure for the synthesis of *E*‐diaminoalkenes is new and bears much potential for enriching the respective compounds’ library.^17^ Two C−H bonds are cleaved during this process, and two new C−H bonds are formed. The formation of a new C=C double bond likely provides the thermodynamic driving force for this rearrangement. In the absence of direct experimental evidence for the transient two‐electron reduced species [**5^H^**] and [**5^Me^**], these were eventually investigated computationally. For [**5^H^**], the singlet state was found to be slightly more stable than the triplet state with a singlet–triplet gap of 26 cm^−1^.

**Figure 4:**
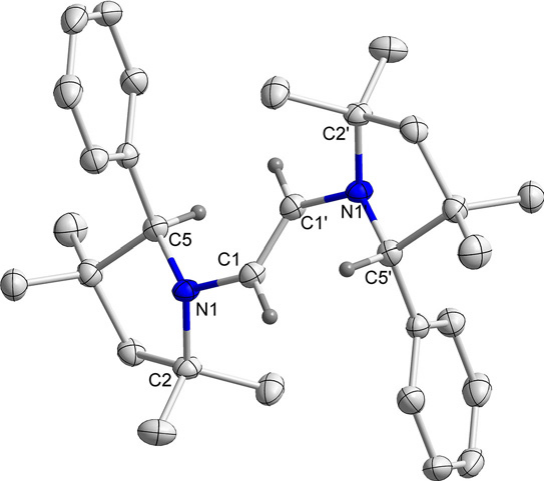
Molecular structure of **6^H^** with thermal ellipsoids at the 50 % probability level. All hydrogen atoms except at C1 and C1′ have been omitted for clarity. Selected bond lengths (Å) and bond angles (°): C1−C1′ 1.332(2), N1−C1 1.407(2), N1−C2 1.499(1), N1−C5 1.456(2); C1‐N1‐C2 115.82(9), C1‐N1‐C5 119.40(9), C5‐N1‐C2 109.66(9), N1‐C1‐C1′ 127.1(1).

The ^1^H NMR spectra of **6^H^** and **6^Me^** exhibit singlets at *δ*=4.84 and 4.89 ppm, respectively, which arise from the vinylic C−H moieties as confirmed by 2D ^13^C{^1^H}‐^1^H correlation NMR spectroscopy. Analyses of the molecular structures of **6^H^** and **6^Me^** show the central C−C bonds to be 1.332(2) and 1.338(3) Å long, respectively, which falls in the range of a typical C=C double bond distance (Figure 4 and Figure S4 in Supporting Information).^18^ Two‐electron reductions of compounds **3^H^** and **3^Me^**, thus, led to intramolecular double H‐atom shift generating vinylic‐bridge bearing compounds, **6^H^** and **6^Me^** from ethylene‐bridged compounds.

**Scheme 4:**
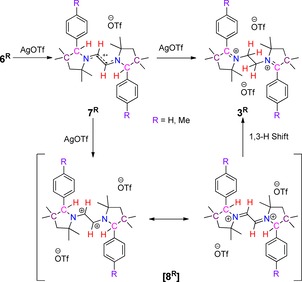
Stepwise reversible oxidations and hydride shift of diamino alkenes **6^R^** to **3^R^**.

After preparing the *E*‐diaminoalkenes **6^H^** and **6^Me^**, it was tested whether these compounds could be oxidized back to **3^H^** and **3^Me^**. To this end, another set of cyclic voltammograms were recorded (Figure 2). Both compounds displayed a one‐electron reversible oxidation step at *E*_1/2_=−0.58 V for **6^H^** and *E*_1/2_=−0.59 for **6^Me^**. Additionally, a second irreversible oxidation step was observed at *E*_1/2_=0.09 V for **6^H^** and at *E*_1/2_=0.06 V for **6^Me^**. Similar to **3^H^** and **3^Me^** (see above), the influence of the substituents on the redox potentials of **6^H^** and **6^Me^** are also marginal. The difference in the redox potentials for **3^H^**/**3^Me^** (Δ*E*_1/2_=0.21 V for **3^H^** and Δ*E*_1/2_=0.19 V for **3^Me^**) is lower compared to the difference for **6^H^**/**6^Me^** (Δ*E*_1/2_=0.67 V for **6^H^** and Δ*E*_1/2_=0.65 V for **6^Me^**) pointing to a larger thermodynamic stability for the one‐electron oxidized forms of **6^H^/6^Me^** as compared to the one‐electron reduced forms of **3^H^/3^Me^**. The difference is likely related to the presence of a conjugated bridge in **6^H^/6^Me^**. The redox potentials of **6^H^**/**6^Me^** clearly point towards an exceptionally electron‐rich nature of these *E*‐diaminoolefins. Chemical oxidations were carried out with AgOTf in a 1:1 reaction affording the corresponding radical cations **7^H^** and **7^Me^**, respectively, in 72 and 79 % yields (Scheme 4).

**Figure 5:**
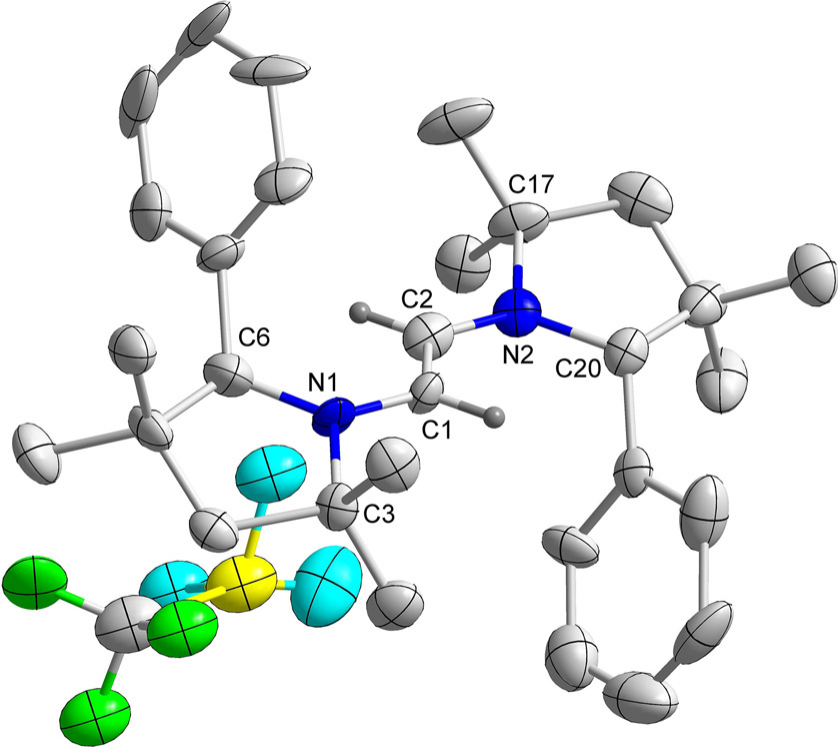
Molecular structure of **7^H^** with thermal ellipsoids at the 50 % probability level. All hydrogen atoms except at C1 and C2 have been omitted for clarity. Selected bond lengths (Å) and bond angles (°): N1−C1 1.370(9), C1−C2 1.358(5), N1−C6 1.40(1), N1−C3 1.488(9); C5‐N1‐C1 125.7(6), C2‐C1‐N1 125.3(4), C6‐N1‐C3 114.5(6).

The molecular structures of **7^H^** and **7^Me^** exhibit central C−C bond distances of 1.358(6) and 1.380(3) Å, which are lengthened compared to the parent *E*‐diaminoalkenes **6^H^** (1.332(2) Å) and **6^Me^** (1.338(3) Å) (Figure 5 and Figure S5 in Supporting Information). Exocyclic N−C bond distances are 1.370(9) and 1.364(5) Å for **7^H^** and **7^Me^**, respectively, indicating partial double bond character there as well.^19^ These unique, structurally characterized radical cations underscore the propensity of the electron‐rich diazaolefinic^20^ compounds **6^H^** and **6^Me^** to undergo clean and facile one‐electron oxidations with the appropriate oxidizing agent.

The reaction of two equivalents of AgOTf with *E*‐diaminoalkenes **6^H^** and **6^Me^** led to the formation of bis‐2‐aryl pyrrolinium cations, **3^H^** and **3^Me^**, respectively, in good yields (Scheme 4).^13^ The oxidation process is accompanied by a double hydride shift,^21^ presumably due to the greater stability of the bis‐1,6‐tertiary carbocations **3^H^** and **3^Me^** in comparison to the initially formed bis‐1,2‐secondary carbocation,^22^ [**8^R^**], which is likely to experience unfavorable coulombic repulsions owing to the presence of two positive charges at very close vicinity. The aforementioned facts provide the thermodynamic driving force for this rearrangement. The rearrangement is accompanied by the cleavage of two C−H bonds and the formation of two new C−H bonds. To the best of our knowledge, such examples of the reversible cleavage and formation of two different C−H bonds both for the forward and reverse reactions in response to two‐electron transfers is unprecedented in transition metal‐free systems. The examples known for organic systems are the cleavage and the formation of carbon–carbon *σ*‐bond in response to an electron transfer step.^23^

As expected, the compounds **6^H^** and **6^Me^** do not display any absorption bands in the visible region. On one‐electron oxidation to **7^H^** and **7^Me^**, bands in the near UV and visible regions appear at 319 and 424 nm, and at 321 and 420 nm, respectively. Such bands are typical for organic radicals. The first electrochemical oxidation step for both compounds turned out to be fully reversible, and these radicals are also very stable. Both **7^H^** and **7^Me^** display well‐resolved, isotropic EPR spectra in fluid solutions at 295 K with *g*‐values of 2.0037 and 2.0033 respectively (Figure 4). The spectra were simulated with high accuracy by considering hyperfine couplings to two equivalent ^14^ N nuclei (A_N_=20.73 MHz), and two sets of two equivalent ^1^H nuclei (A_H1_=32.3 MHz and A_H2_=10.76 MHz). Spin‐density calculations (see Figure S32 in Supporting Information) show the spin‐density to be predominantly located on the vinylic part of the bridge and the two connected nitrogen atoms, with a smaller but significant contribution from the H atoms at the C5 carbons. As mentioned above, the second oxidation steps for both **6^H^** and **6^Me^** appear irreversible in their cyclic voltammograms. This transformation was studied in more detail for **6^Me^**. Following the second electrochemical oxidation, two new reduction signals are observed in the voltammograms at dramatically shifted negative potentials, which appear to match the redox potentials of **3^Me^** (see Figures S20–S26 in Supporting Information). These observations point to the operation of either an EC (electrochemical‐chemical) or a more complex mechanism. The peaks at the large negative potentials are semi‐reversible and account for the significantly decreased cathodic current in the redox transition at 0.06 V (for a fully reversible redox process the anodic and cathodic peak currents have the same height). These new redox responses arise from reductions of the dicationic species formed from [**8^Me^**] (see Figure S20–S26 in Supporting Information). Notably, two distinct sets of spectroscopic changes were observed on monitoring the second oxidation step of this compound via UV/Vis spectroelectrochemistry. The final spectrum generated after the complete second oxidation looks somewhat similar to **3^Me^**. Upon moving the applied potential back to the starting potential, the initial spectrum of **6^Me^** was not regenerated. However, applying more negative potentials which cover also the newly generated reduction steps, and then moving the potential back to the starting point recovered a spectrum corresponding to **6^Me^**. These experiments, thus, point to the operation of rather fast chemical reactions during the redox‐induced conversion of **6^Me^** to **3^Me^** via **7^Me^** on timescales which can be monitored via UV/Vis spectroelectrochemistry. The aforementioned signals provide the first evidence for the operation of bistability in these systems. Without any direct experimental evidence for the presence of [**8^H^**] and [**8^Me^**], the former was investigated computationally. The calculations for [**8^H^**] show that the bridge's C−C bond length increased from 1.388 in **7^H^** to 1.466 Å, while the bridge's C−N bond lengths shortened from 1.330 in **7^H^** to 1.270 Å in [**8^H^**] (see Figure S35 in Supporting Information), which supports the bis‐iminium resonance structure of [**8^H^**] (Scheme 4).

Thus, the conversion of **3^H^**/**3^Me^** to **6^H^**/**6^Me^** occurs by a two‐electron reduction induced two‐H‐atom shift, while the re‐conversion of **6^H^**/**6^Me^** to **3^H^**/**3^Me^** takes place through a two‐electron oxidation induced two‐hydride shift. A total of four C−H bonds are cleaved and four C−H bonds are formed during the combined forward and reverse reactions. The experimental observations are supported by Gibbs free enthalpy calculations on optimized structures of species **3^R^** to [**8** ^R^] (see Table S3 in Supporting Information). The calculations show that **3^H^**/**3^Me^** are slightly more stable than [**8** ^H^]/[**8** ^Me^] species, while **6^H^**/**6^Me^** are slightly more stable than [**5^H^**]/[**5^Me^**]. When **3^H^**/**3^Me^** and **6^H^**/**6^Me^** were mixed in a 1:1 ratio in THF no reaction took place, which is in contrast to NHC‐CAAC derived triazaalkenes, which undergo comproportionation reactions.^24^ Even though the interconversion between the reduced and oxidized species is reversible, the forward and the backward reaction pathways are completely distinct. Still, the interconversion of **3^H^**/**3^Me^** and **6^H^**/**6^Me^** can be considered globally reversible even though the second redox events in each direction are individually electrochemically irreversible.

## Conclusions

In conclusion, we have synthesized bis‐2‐aryl‐pyrrolinium salts, where the *N*‐centers are bridged by a ‐CH_2_CH_2_‐ linker. This novel synthetic route provides a very convenient access to multi‐CAAC‐scaffold systems without the need of using free CAACs as starting materials. Notably, with this synthetic route an extensive substrate scope can be realized with substitution derivatives of both the aryl moieties and the diamine bridge. The reported procedure, thus, holds exceptional promise for synthesizing next generation multi‐CAAC scaffold containing compounds. *N*,*N′*‐Ethylene‐bridged bis‐2‐aryl‐pyrrolinium cations undergo a stepwise two‐electron reduction, yielding the *E*‐diaminoalkenes. The first one‐electron reduction affords the radical cation, which undergoes a second one‐electron reduction to a diradical. The latter, subsequently, and immediately abstracts two hydrogen atoms from the ethylene bridge forming the electron‐rich *E*‐diaminoalkenes. Upon a two‐electron oxidation, the starting bis‐2‐aryl‐pyrrolinium salts can be recovered. This process is also sequential: the first oxidation leads to a crystalline radical cation and subsequently the corresponding dication. The latter undergoes a 1,3‐hydrogen shift yielding the bis‐2‐aryl‐pyrrolinium salts. Overall, a total of four C−H bonds are cleaved, and four C−H bonds are formed reversibly in response to redox processes. All intermediates in the forward and the reverse cycle have been thoroughly studied by experiment and theory. Unlike many reversible electron transfer‐induced bond activation processes, which occur through common chemical intermediates, the events described in this study do not follow this standard paradigm. Instead, the chemical pathways in the reduction and oxidation events are completely non‐identical. The results unequivocally show that such multi‐CAAC‐scaffold systems are capable of multi‐electron transfer involving chemical reactivity that is traditionally the realm of transition metal complexes. To the best of our knowledge, this is an unprecedented observation in electron transfer induced bond activation reactions. The novel systems presented here can therefore not only mimic transition metal complexes in undergoing reversible multi‐electron transfer‐induced chemical reactions (EEC mechanisms), but also do so in a completely unique way.

## Conflict of interest

The authors declare no conflict of interest.

## Supporting information

As a service to our authors and readers, this journal provides supporting information supplied by the authors. Such materials are peer reviewed and may be re‐organized for online delivery, but are not copy‐edited or typeset. Technical support issues arising from supporting information (other than missing files) should be addressed to the authors.

Supplementary
