# Supplementary material for: N,N′‐Ethylene‐Bridged Bis‐2‐Aryl‐Pyrrolinium Cations to E‐Diaminoalkenes: Non‐Identical Stepwise Reversible Double‐Redox Coupled Bond Activation Reactions
Source: Chemistry. 2020 Mar 9;26(19):4425–31. doi: 10.1002/chem.202000255 (PMC7187269; doi:10.1002/chem.202000255)
Supplement: Supplementary file 1 — Supplementary [file CHEM-26-4425-s001.pdf]

CHEMISTRY
A European Journal

Supporting Information

N,N′-Ethylene-Bridged Bis-2-Aryl-Pyrrolinium Cations to *E*-Diaminoalkenes: Non-Identical Stepwise Reversible Double-Redox Coupled Bond Activation Reactions

Mithilesh Kumar Nayak,[a] Jessica Stubbe,[b] Nicolás I. Neuman,[b, c] Ramakirushnan Suriya Narayanan,[a] Sandipan Maji,[a] Carola Schulzke,*[d] Vadapalli Chandrasekhar,*[a, e] Biprajit Sarkar,*[b, f] and Anukul Jana*[a]

chem_202000255_sm_miscellaneous_information.pdf

1.	Content	S1
2.	General Considerations	S2
3.	Experimental Details and Analytical Data	S4
4.	Molecular Structures of **2^H^**, **2^Me^**, **3^Me^**, **6^Me^**, and **7^Me^**	S10
5.	Cyclic Voltammetry	S13
6.	UV/vis Spectroelectrochemistry	S17
7.	Electrochemical Study of Bistability	S19
8.	EPR Spectroelectrochemistry	S22
9.	DFT Calculations	S25
10.	NMR Spectra	S49
11.	UV/vis Spectra	S56
12.	Crystallographic Details	S59
13.	References	S69

General Considerations

All experiments were carried out under an argon atmosphere using standard Schlenk techniques or in a PL-HE-2GB Innovative Technology GloveBox. *n*-Hexane, diethyl ether, THF, and toluene were dried by PS-MD-5 Innovative Technology solvent purification system. $\mathbf{1^{Me}}$ was prepared according to literature procedure.[S1] All other chemicals were purchased commercially (ethylenediamine - Alfa Aesar, isopropyl phenyl ketone, $\mathbf{1^{H}}$ - TCI Chemicals, potassium - Sigma Aldrich, graphite -Sigma Aldrich, isobutylene oxide - TCI Chemicals, triflic anhydride - TCI Chemicals, *n*BuLi - Hychem Laboratories, diisopropylamine - Avra Chemicals, AgOTf - Sigma Aldrich, pTsOH - Sigma Aldrich, isobutyryl chloride - TCI Chemicals, aluminium chloride - Sigma Aldrich) and used as received, except ethylenediamine and diisopropylamine which were distilled over KOH before use. Benzene-d6 was dried and distilled over potassium under argon. Chloroform-d1 and acetonitrile-d3 were dried and distilled over CaH_2 under argon. NMR spectra were recorded on a BrukerNanoBay 300 MHz NMR spectrometer. ^{1}H and $^{13}C\{^{1}H\}$ NMR spectra were referenced to the peaks of residual protons of the deuterated solvent (^{1}H) or the deuterated solvent itself (^{13}C). $^{19}F\{^{1}H\}$ NMR spectra were referenced to external tol-CF_3. UV/vis spectra were acquired with a Jasco V-670 spectrometer using quartz cells with a path length of 0.1 cm.

EPR spectra at X-band frequency (ca. 9.5 GHz) were obtained with a Magnettech MS-5000 benchtop EPR spectrometer equipped with a rectangular TE 102 cavity and an evaporated liquid N_2 cooling system with a TC HO4 temperature controller. The measurements were carried out in synthetic quartz glass tubes. For EPR spectroelectrochemistry a three-electrode setup was employed using two teflon-coated platinum wires (0.005” bare, 0.008” coated) as working and counter electrode and a Teflon-coated silver wire (0.005” bare, 0.007” coated) as pseudoreference electrode. Spectral simulations were performed with EasySpin 5.1.4[S2] and MatLab R2012a.

Cyclic voltammograms were recorded with a PAR VersaStat 4 potentiostat (Ametek) by working in anhydrous and degassed acetonitrile with 0.1 M NBu_4PF_6 (dried, > 99.0%, electrochemical grade, Fluka) as supporting electrolyte. Concentrations of the compounds were about $1 \cdot 10^{-4}$ M. A three-electrode setup was used with a glassy working electrode, a coiled platinum wire as counter electrode, and a coiled silver wire as a pseudoreference electrode. The decamethylferrocene/decamethylferrocenium couple was used as internal reference.

UV/vis spectra were recorded with an Avantes spectrometer consisting of a light source (AvaLight-DH-S-Bal), a UV/vis detector (AcaSpec-ULS2048), and a NIR detector (AvaSpec-NIR256-TEC). Spectroelectrochemical measurements were carried out in an optically transparent thin-layer electrochemical (OTTLE) cell (CaF_2 windows) with a gold working electrode, a platinum mesh counter electrode, and a silver-foil pseudoreference electrode. Anhydrous and degassed acetonitrile with 0.1 M NBu_4PF_6 as supporting electrolyte was used.

Elemental analyses were performed on a Perkin Elmer Analyser 240. Mass spectrometry measurements were performed on an Agilent 6210 ESI-TOF. Melting points were determined in closed NMR tubes under argon atmosphere and are uncorrected.

For density functional theory calculations, the ORCA 4.0.1 program package was used for all DFT calculations.[S3] Geometry optimizations and single point calculations were performed using the PBE0[S4] functional with a def2-TZVP basis set on all atoms,[S5] using default convergence settings. All calculations were run including empirical Van der Waals corrections (D3).[S6-S9] The resolution-of-the-identity (RI) approximation[S10-S116] was employed to reduce calculation times, together with the auxiliary Def2/J basis set[S17-S18] and the RIJCOSX (combination of the resolution of identity and chain of spheres algorithms) approximation.[S10-S16] Solvation effects were accounted for by using the conductor-like polarizable continuum model (CPCM), using acetonitrile as implicit solvent.[S19] Numerical frequency calculations were performed to confirm the presence of structural minima. Spin densities were visualized using the modified Avogadro 1.2.0 program with extended ORCA support.[S20-S21]

Experimental Details and Analytical Data

Synthesis of 2^H. Method I (without catalyst). 80.0 mL of benzene was added to a 250 mL Schlenk flask containing $\mathbf{1^H}$ (15.0 mL, 100.0 mmol) and ethylenediamine (3.4 mL, 50.0 mmol). The resulting mixture was refluxed using a Dean-Stark apparatus for 10 h. Subsequently benzene and all the volatilities were removed by vacuum distillation. The title compound was separated at 180 °C and 0.2 torr. Then, it was crystallized out from hot *n*-hexane and the resulting crystals were washed with small amounts of *n*-hexane to give the pure desired compound $\mathbf{2^H}$. Yield: 8.0 g (50 %). **Method II (using *p*TsOH as a catalyst).** $\mathbf{1^H}$ (63.0 ml, 420.0 mmol) and catalytic amounts of *p*-toluenesulfonic acid monohydrate (200.0 mg, 1.05 mmol) were added to a 250 mL Schlenk flask and stirred for half an hour at room temperature. Then, benzene (100.0 mL) and ethylenediamine (33.4 mL, 200.0 mmol) were added. The resulting mixture was refluxed using a Dean-Stark apparatus for 10 h at 100 °C and six hours at 120 °C. Subsequently benzene and all the volatilities were removed by vacuum distillation. The title compound was separated at 180 °C and 0.2 torr. Then, it was crystallized out from hot *n*-hexane and the resulting crystals were washed with small amounts of *n*-hexane to give the pure desired compound $\mathbf{2^H}$. The resulting crystals were suitable for single crystal X-ray diffraction analysis. Yield: 57.0 g (89%). **^{1}H** NMR ($CDCl_3$, 25 °C, 300 MHz): δ = 7.3−7.4 (m, 6H, Ar-*H*), 6.7−7.07 (m, 4H, Ar-*H*), 3.36 (s, 4H, CH_2CH_2), 2.67-2.84 (sept, 2H, *J* = 6.91 Hz, C*H*), 1.05-1.07 (d, 12H, *J* = 6.93 Hz, CH_3) ppm. **^{13}C{^{1}H}** NMR ($CDCl_3$, 25 °C, 75.4 MHz): δ = 138.4, 128.5, 128.0, 127.3, 54.3, 39.5, 20.5 ppm. **ESI-MS**: [M] = $C_{22}H_{28}N_2$; calcd. $([M]+(H^+))^+$: 321.2325; found: 321.2080, Δ = 76.3 ppm. **Elemental analysis** calcd. (%) for $C_{22}H_{28}N_2$: C 82.45, H 8.81, N 8.74; found: C 82.74, H 9.26, N 8.79.

Synthesis of 2^{Me}. 1^{Me} (18.0 g, 110.0 mmol) and catalytic amounts of *p*-toluenesulfonic acid (100.0 mg, 0.526 mmol) were added to a 250 mL Schlenk flask and stirred for half an hour at room temperature. Then, benzene (80.0 mL) and ethylenediamine (3.4 mL, 50.0 mmol) were added. The resulting mixture was refluxed using a Dean-Stark apparatus for four hours at 100 °C and six hours at 120 °C. Subsequently benzene and all the volatilities were removed by vacuum

distillation. The title compound was separated at 210−240 °C and 0.18-0.15 torr. Then, it was crystallized out from hot *n*-hexane and washed with small amounts of *n*-hexane to give the desired compound **2^{Me}**. Yield: 15.6 g (90%). **^{1}H** NMR ($CDCl_3$, 25 °C, 300 MHz): δ = 7.15-7.18 (d, 4H, J = 7.8 Hz, Ar-H), 6.7-6.93 (d, 4H, , J = 8.0 Hz, Ar-H), 3.36 (s, 4H, CH_2CH_2), 2.65-2.78 (sept, 2H, J = 6.93 Hz, CH), 2.37 (s, 6H, Ar-CH_3), 1.03-1.06 (d, 12H, J = 6.93 Hz, CH_3) ppm. **^{13}C{^{1}H}** NMR ($CDCl_3$, 25 °C, 75.4 MHz): δ = 177.3, 137.6, 135.4, 129.1, 127.2, 54.3, 39.6, 21.6, 20.5 ppm. **ESI-MS**: [M] = $C_{24}H_{32}N_2$; calcd. ([M]+(H^+))$^+$: 349.2638; found: 349.2698, Δ = 17.2 ppm. **Elemental analysis** calcd. (%) for $C_{24}H_{32}N_2$: C 82.71, H 9.25, N 8.04; found: C 82.86, H 10.56, N 8.05.

Synthesis of 3^{H}. Freshly prepared 30.0 mmol of LDA solution in THF (concentration is about 0.645 M) was added to a Et_2O solution of **2^{H}** (4.8 g, 15.0 mmol in 80.0 mL of Et_2O) at −78 °C and the reaction mixture was slowly allowed to warm up to room temperature. Then, the resulting reaction mixture was stirred for 12 hrs at room temperature and afterwards all solvents and volatilities were removed under vacuum. Then, 250 mL of Et_2O was added, followed by the dropwise addition of isobutylene oxide (2.7 mL, 30.4 mmol) at 0 °C. The resulting reaction mixture was allowed to warm up to room temperature within 2 h. After that, the reaction mixture was stirred at room temperature for another 12 h. Subsequently trifluoromethane sulfonic anhydride (5.1 mL, 30.3 mmol) was added dropwise at −78 °C. The reaction mixture was allowed to slowly reach room temperature and stirred for another 4 h. Then, the resulting solution was filtered and the residue was washed 10 times with 5 mL of DCM. The resulting residue was dissolved in warm CH_3CN and slow diffusion of Et_2O led to the desired compound **3^{H}** as colorless crystals, suitable for single crystals X-ray diffraction analysis. The crystals were filtered and washed with diethylether. The filtrate was concentrated and kept for further crystallization by layering with DCM. Total yield: 2.63 g (24%). **M.P.** > 200 °C. **^{1}H** NMR (CD_3CN, 25 °C, 300 MHz): δ = 7.69−7.81 (m, 6H, Ar-H), 7.51−7.53 (m, 4H, Ar-H), 3.71 (s, 4H, CH_2CH_2), 2.22 (s, 4H, CH_2), 1.31 (s, 12H, CH_3), 1.20 ppm (s, 12H, CH_3); **^{13}C{^{1}H}** NMR (CD_3CN, 25 °C, 75.4 MHz): δ = 198.5 ($C_{iminium}$), 133.6 (Ar-C), 130.8 (Ar-C), 127.0 (Ar-C), 78.5 (C(CH_3)$_2$), 51.6 (C(CH_3)$_2$), 48.07 (CH$_2$), 44.7 (CH$_2$-CH$_2$), 26.9 (CH$_3$), 26.6 (CH_3) ppm. **^{19}F{^{1}H}** NMR (CD_3CN, 25 °C, 282 MHz): δ = −79.3 ppm. **ESI-MS**: [M] = $C_{32}H_{42}F_6N_2O_6S_2$; calcd. ([M]−($^{-}$OTf))$^+$: 579.2863; found: 579.2846, Δ

= 2.9 ppm; calcd. ([M]−($^{-}$OTf)$_2$−H$^+$)$^+$: 429.3264; found: 429.3253; Δ = 2.6 ppm. **Elemental analysis** calcd. (%) for $C_{32}H_{42}F_6N_2O_6S_2$: C 52.74, H 5.81, N 3.84, S 8.80; found: C 52.77, H 5.88, N 3.87, S 8.82.

Synthesis of 3^{Me}. Freshly prepared 51.6 mmol of LDA solution in THF (concentration is about 0.645 M) was added to the Et_2O solution of **2^H** (9g , 25.82 mmol in 100 mL of Et_2O) at −78 °C and the reaction mixture was allowed to warm up to room temperature slowly. Then the resulting reaction mixture was stirred for 12 hrs at room temperature and after that all the solvents and volatiles were removed under vacuum. After that 250 mL of Et_2O was added and then isobutylene oxide (4.6 mL, 51.7 mmol) was added dropwise at 0 °C and then the resulting reaction mixture was allowed to come to room temperature slowly in 2 hrs. After that the reaction mixture was stirred at room temperature for another 12 hrs. Subsequently trifluoromethane sulfonic anhydride (8.7 mL, 51.7 mmol) was added dropwise at −78 °C. The reaction mixture was allowed to reach room temperature slowly and stirred for another 4 hrs at room temperature. After that the resulting solution was filtered and the residue was washed with diethyl ether (20 mL). Then the resulting residue was dissolved in warm CH_3CN and slow diffusion of Et_2O into it leads to title compound **3^{Me}** as colorless crystals after 1 day and which were also suitable for single crystals X-ray diffraction analysis. Crystals were filtered, and washed with ether. The filtrate was concentrated and kept for further crystallization by layering with DCM. Total yield: 5 g (26%). **M.P.** > 200 °C. **^{1}H** NMR (CD_3CN, 25 °C, 300 MHz): δ = 7.50−7.53 (d, 4H, J = 8.12 Hz, Ar-H), 7.36−7.38 (d, 4H, J = 8.28 Hz, Ar-H), 3.67 (s, 4H, CH_2CH_2), 2.50 (s, 6H, Ar-CH_3), 2.20 (s, 4H, CH_2), 1.30 (s, 12H, CH_3), 1.21 ppm (s, 12H, CH_3); **^{13}C{^{1}H}** NMR (CD_3CN, 25 °C, 75.4 MHz): δ = 198.7 ($C_{iminium}$), 144.6 (Ar-C), 131.2 (Ar-C), 126.9 (Ar-C), 123.6 (Ar-C), 78.2 (C(CH$_3$)$_2$), 51.4 (C(CH$_3$)$_2$), 48.1 (CH$_2$), 44.4 (CH$_2$-CH$_2$), 26.82 (CH$_3$), 26.6 (CH$_3$), 21.5 (Ar-CH$_3$) ppm. **^{19}F{^{1}H}** NMR (CD_3CN, 25 °C, 282 MHz): δ = −79.3 ppm. **ESI-MS**: [M] = $C_{34}H_{46}F_6N_2O_6S_2$; calcd. ([M]−($^{-}$OTf))$^+$: 607.3176; found 607.3173; Δ = 0.5 ppm; calcd. ([M]−($^{-}$OTf)$_2$−H$^+$)$^+$: 457.3577; found 457.3577; Δ = 0 ppm. **Elemental analysis** calcd. (%) for $C_{34}H_{46}F_6N_2O_6S_2$: C 53.96, H 6.13, N 3.70, S 8.47; found: C 53.96, H 6.15, N 3.78, S 8.51.

Synthesis of 6^H. 3^H (1.44 g, 1.98 mmol) and potassium graphite (697.00 mg, 5.15 mmol) were added to a 50 ml Schlenk flask inside the GloveBox and 80 mL of THF was added using Schlenk technique at room temperature outside the GloveBox. The reaction solution became initially red and then colorless. After 2 h of stirring, all the volatiles were removed under vacuum and the resulting residue was extracted two times with 40 mL of *n*-hexane. Evaporation of the solvent under reduced pressure led to the isolation of the desired product $\mathbf{6^H}$ as white solid. Yield: 555.0 mg (65 %). A concentrated *n*-hexane solution of the compound at −30 °C led to the formation of single crystals suitable for single crystal X-ray diffraction analysis. **M.P.**: 173°C. **^{1}H** NMR (C_6D_6, 25 °C, 300 MHz): δ = 7.26−7.09 (m, 10H, Ar-*H*), 4.86 (s, 2H, $CHCH_{bridging}$), 3.73 (s, 2H, Ar-C*H*), 1.52−1.49 (m, 4H, CH_2), 1.09 (s, 6H, CH_3), 0.98 (s, 6H, CH_3), 0.64 (s, 6H, CH_3), 0.53 ppm (s, 6H, CH_3); **^{13}C{^{1}H}** NMR (C_6D_6, 25 °C, 75.4 MHz): δ = 141.7, 127.3, 119.0, 73.7, 60.7, 55.8, 40.6, 29.7, 29.3, 28.1, 27.2 ppm. **ESI-MS**: [M] = $C_{30}H_{42}N_2$; calcd. $([M])^+$: 430.3348; found 430.3336; Δ = 2.8 ppm. **Elemental analysis** calcd. (%) for $C_{30}H_{42}N_2$: C 83.67, H 9.83, N 6.50; found: C 83.96, H 11.87, N 6.54.

Synthesis of 6^{Me}. 3^{Me} (1.5 g, 1.97 mmol) and potassium graphite (695 mg, 5.13 mmol) were taken into a 50 ml Schlenk flask inside the GloveBox and about 80 ml of THF was added using Schlenk technique at room temperature outside the GloveBox. The reaction solution became initially red and then colorless. After 2 hrs of stirring at room temperature all the volatiles were removed under vacuum and the resulting residue was extracted with 40 mL of *n*-hexane (2 times). After evaporating the resulting filtrate we obtained titled compound $\mathbf{6^{Me}}$ as a white solid. Yield: 660 mg (73 %). Suitable single crystals of $\mathbf{6^{Me}}$ for X-ray diffraction were obtained after keeping a concentrated *n*-hexane solution at −30 °C for 6 hrs. **M.P.**: 169 °C. **^{1}H** NMR (C_6D_6, 25 °C, 300 MHz): δ = 7.21-7.19 (d, 4H, *J* = 7.33 Hz, Ar-*H*), 7.06-7.03 (d, 4H, *J* = 8.01 Hz, Ar-*H*), 4.90 (s, 2H, $CHCH_{bridging}$), 3.74 (s, 2H, Ar-C*H*), 2.20 (s, 6H, Ar-CH_3), 1.59−1.47 (m, 4H, CH_2), 1.11 (s, 6H, CH_3), 1.0 (s, 6H, CH_3), 0.7 (s, 6H, CH_3), 0.58 ppm (s, 6H, CH_3); **^{13}C{^{1}H}** NMR (C_6D_6, 25 °C, 75.4 MHz): δ = 138.7, 136.4, 129.1, 119.3, 73.6, 60.6, 55.8, 40.6, 29.6, 29.4, 28.0, 27.1, 21.7 ppm. **ESI-MS**: [M] = $C_{32}H_{46}N_2$; calcd. $([M])^+$: 458.3656; found 458.3664; Δ = 1.7 ppm. **Elemental analysis** calcd. (%) for $C_{32}H_{46}N_2$: C 83.79, H 10.11, N 6.11; found: C 83.79, H 11.36, N 6.16.

Synthesis of 7^{H}. THF (4 mL) was added to a 25 ml Schlenk flask containing **6^{H}** (110.0 mg, 0.3 mmol) and silver triflate (57.0 mg, 0.2 mmol) at room temperature and stirred inside the GloveBox. Immediately the formation of metallic silver was observed as evidenced by its black colour. After 30 seconds of stirring, the reaction mixture was allowed to stand for 10 minutes to sediment metallic silver. The orange-red supernatant solution was decanted and kept at −30 °C with *n*-pentane diffusion for crystallization. Yellow crystals of **7^{H}** were obtained after 12 h and suitable for single crystal X-ray diffraction analysis. The crystals were washed with 5 mL of *n*-hexane and the mother liquor was kept again at −30 °C for a second crop of crystals. Total yield: 92.6 m g (72%). **M.P.**: 189 °C. **UV/vis** (THF): $\lambda_{max}(\varepsilon)$ = 389 (5647), 321 (7697), 256 (5096) nm (L·mol^{-1}cm^{-1}). **ESI-MS**: [M] = $C_{31}H_{42}F_3N_2O_3S$; calcd. ([M]–($^{-}$OTf)–H)$^{+}$: 429.3264; found 429.3393; Δ = 30.0 ppm. **Elemental analysis** calcd. (%) for $C_{31}H_{42}F_3N_2O_3S$: C 64.23, H 7.30, N 4.83, S 5.53; found: C 63.65, H 7.30, N 4.59, S 5.95.

Synthesis of 7^{Me}. THF (4 mL) was added to 25 ml Schlenk flask containing **6^{Me}** (110 mg, 0.24 mmol) and silver triflate (54 mg, 0.21 mmol) at room temperature with stirring inside the GloveBox. Immediately the formation of metallic silver was observed as evidenced by its black colour. After 30 seconds of stirring, the reaction mixture was allowed to stand for 10 minutes to sediment metallic silver. The orange-red supernatant solution was taken out using a glass-pipette and kept at −30 °C with *n*-pentane diffusion for crystallization. Intense reddish orange colored crystals of **7^{Me}** were obtained after 12 hrs and after collecting resulting crystals (which were also suitable for single crystal X-ray diffraction analysis) they were washed with 5 mL of *n*-hexane. After concentrating the mother liquor it was kept again at −30 °C for a second crop of crystals. Total yield: 100.8 m g (79 %). **M.P.**: 178 °C. **UV/vis** (THF): $\lambda_{max}(\varepsilon)$ = 411 (2810), 321 (8153), nm (Lmol^{-1}cm^{-1}). **ESI-MS**: [M] = $C_{33}H_{46}F_3N_2O_3S$; calcd. ([M]–($^{-}$OTf))$^{+}$: 458.3656; found 458.3802; Δ = 31.9 ppm. **Elemental analysis** calcd. (%) for $C_{33}H_{46}F_3N_2O_3S$: C 65.21, H 7.63, N 61, S 5.27; found: C 64.33, H 7.65, N 4.66, S 5.37.

Synthesis of 3^{H} via Oxidation of 6^{H}. A 5 mL THF solution of **6^{H}** (50.0 mg, 0.1) was added dropwise to a 5 mL THF solution of silver triflate (70.0 mg, 0.3 mmol) at room temperature under stirring. Immediately the formation of metallic silver was observed as evidenced by its black colour. After 30 minutes, the stirring was interrupted to allow the silver to settle and the supernatant solution was decanted, concentrated and kept at −30 °C with *n*-pentane diffusion for crystallization. Colorless crystals of **3^{H}** were obtained after 12 h. Yield: 38.0 mg (45 %).

Synthesis of 3^{Me} via Oxidation of 6^{Me}. A THF solution of **6^{Me}** (50 mg, 0.109 mmol in 5 mL of THF) was added dropwise to a THF solution of silver triflate (70 mg, 0.272 mmol in 5 mL of THF) at room temperature under stirring. Immediately the formation of metallic silver was observed as evidenced by its black colour. After stirring the reaction mixture for 30 minutes, it was allowed to stand for 10 minutes to sediment metallic silver. Then the supernatant solution was taken out and concentrating to about 4 mL and kept at −30 °C with *n*-pentane diffusion for crystallization. Colorless crystals of **3^{Me}** were obtained after 12 hrs. Yield: 48 mg (58 %).

Molecular Structures of 2^{H}, 2^{Me}, 3^{Me}, 6^{Me}, and 7^{Me}

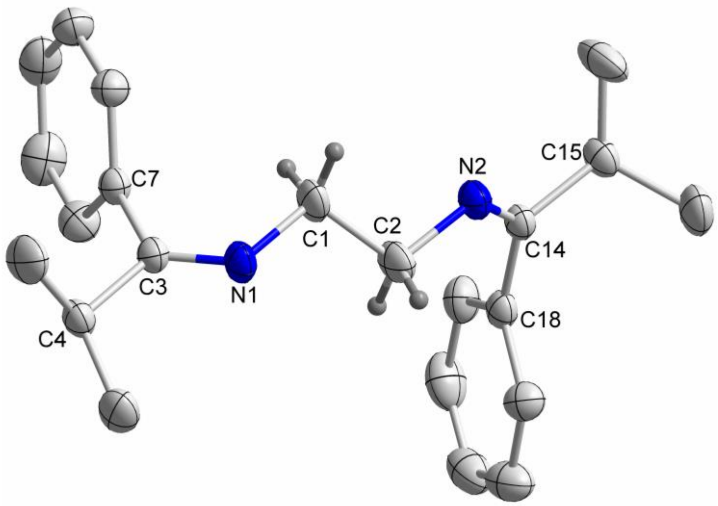

Figure S1. Molecular structure of 2^{H} with thermal ellipsoids at the 50 % probability level. Selected bond lengths (Å) and bond angles (°): C1–C2 1.494(5), N1–C1 1.462(4), C2–N2 1.468(5), N2–C14 1.275(5), C14–C18 1.497(5), C14–C15 1.521(4), N1–C3 1.269(4), C3–C7 1.506(5), C3–C4 1.513(4); C2–N2–C14 119.8(3), N2–C14–C15 119.0(3), N2–C14–C18 125.7(3), N2–C2–C1 109.5(3), C2–C1–N1 108.9(3), C1–N1–C3 119.9(3), N1–C3–C4 118.9(3), N1–C3–C7 125.6(3).

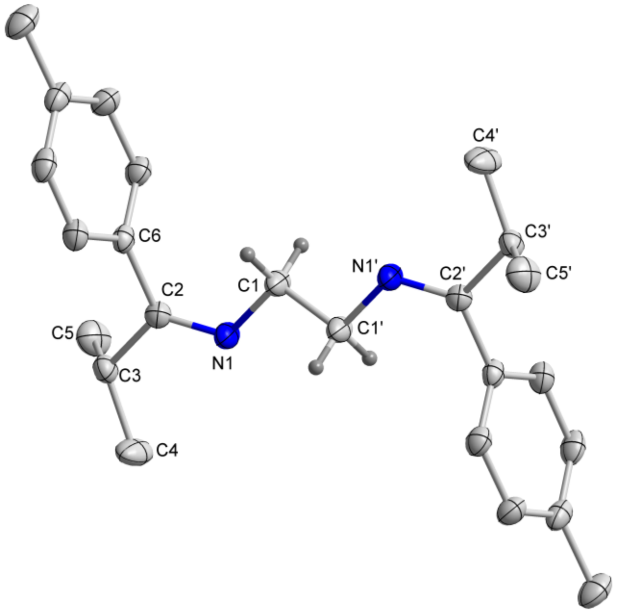

Figure S2. Molecular structure of 2^{Me} with thermal ellipsoids at the 50 % probability level. Selected bond lengths (Å) and bond angles (°):C1–C1' 1.501(4), N1–C1 1.468(3), N1–C2 1.276(3), C2–C3 1.519(3), C3–C4 1.522(3), C3–C5 1.522(3), C2–C6 1.503(3); C1–N1–C2 119.4(2), N1–C2–C3 119.4(2), N1–C2–C6 124.6(2), C2–C3–C4 113.3(2), C2–C3–C5 109.8(2), N1–C1–C1’ 109.4(2), C3–C2–C6 116.0(2).

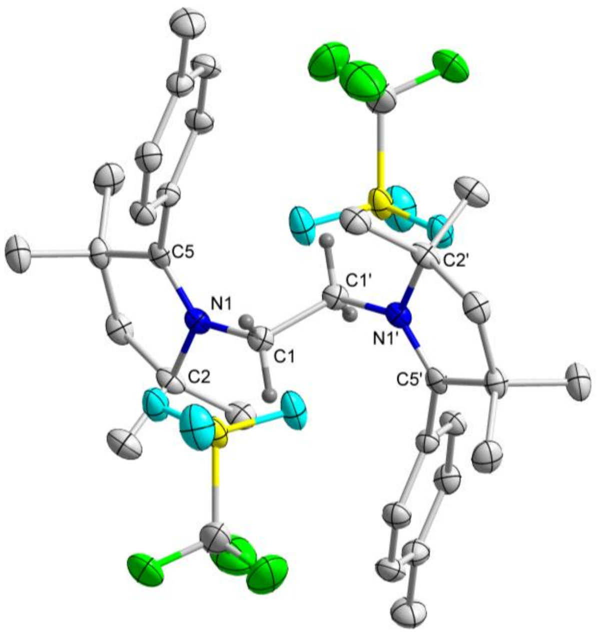

Figure S3. Molecular structure of $\mathbf{3^{Me}}$ with thermal ellipsoids at 50 % probability level. All hydrogen atoms except at C1 and C1' have been omitted for clarity. Selected bond lengths (Å) and bond angles (°): C1–C1' 1.533(3), N1–C1 1.475(2), N1–C2 1.516(2), N1–C5 1.291(2); C1–N1–C2 121.3(2), C1–N1–C5 124.5(1), C5–N1–C2 114.3(1), N1–C1–C1' 109.1(1).

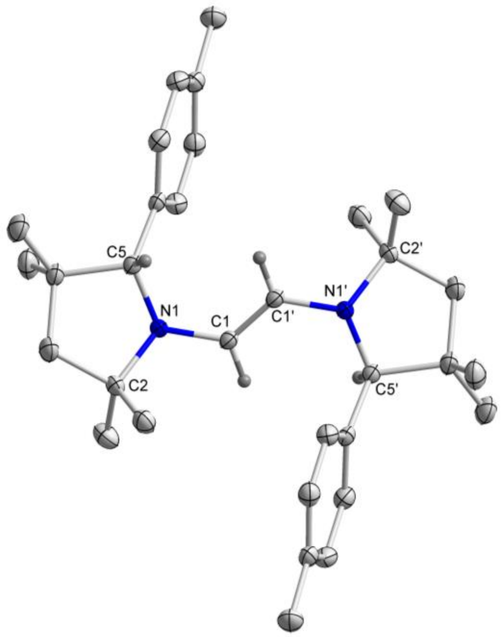

Figure S4. Molecular structure of $\mathbf{6^{Me}}$ with thermal ellipsoids at the 50% probability level. Selected bond lengths (Å) and bond angles (°): C1–C1' 1.338(3), N1–C1 1.411(2), N1–C2 1.497(2), N1–C5 1.458(2); C1–N1–C2 115.6(1), C1–N1–C5 118.6(1), C5–N1–C2 109.2(1), N1–C1–C1' 126.6(2).

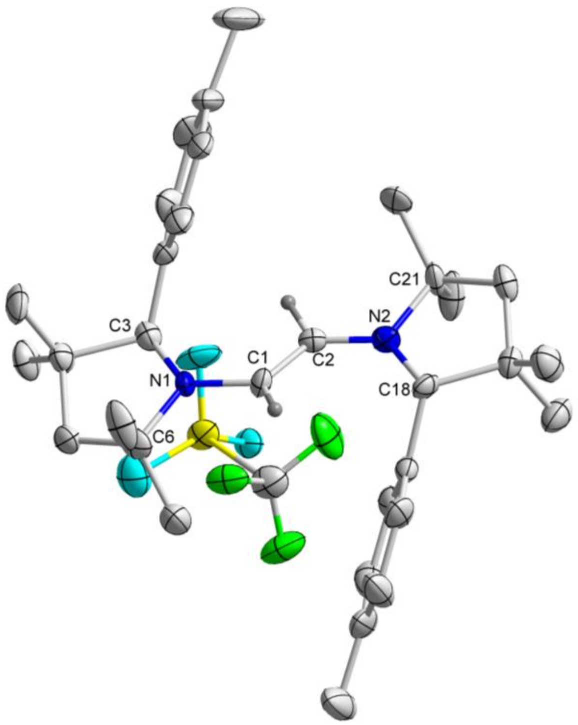

Figure S5. Molecular structure of $\mathbf{7}^{\mathbf{Me}}$ with thermal ellipsoids at the 50 % probability level. Selected bond lengths (Å) and bond angles (°): N1–C1 1.364(5), N1–C6 1.434(6), N1–C3 1.489(6), C1–C2 1.380(3), C2–N2 1.311(6), N2–C21 1.496(6), C18–N2 1.498(6); C1–N1–C6 124.1(4),, C3–N1–C6 115.1(4), N1–C1–C2 123.9(3), C1–C2–N2 125.3(3), C2–N2–C21 121.4(4), C2–N2–C18 123.9(4), C21–N2–C18 113.4(4).

Cyclic Voltammetry

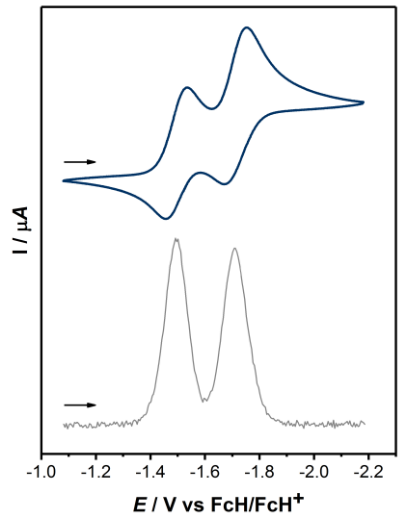

Potential	Peak-to-peak separation (ΔE)
$E_{1/2}$(1.Red) = −1.50 V	80.05 V
$E_{1/2}$(2.Red) = −1.71 V	82.19 V

Figure S6. Cyclic voltammogram (top) and differential potential voltammogram (bottom) of $\mathbf{3^H}$ in MeCN at 100 mV·s^{-1} (CV) and 20 mV·s-1 (dpv) with 0.1 M Bu_4NPF_6.

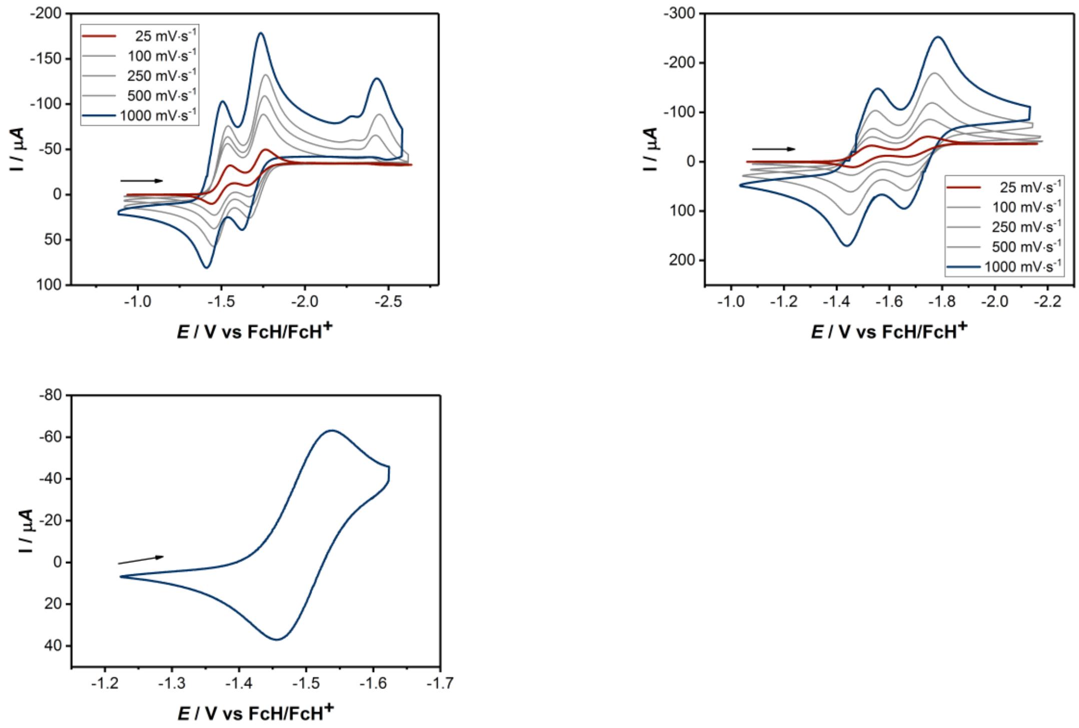

Figure S7. Cyclic voltammograms of $\mathbf{3^H}$ in MeCN at different scan rates with 0.1 M Bu_4NPF_6.

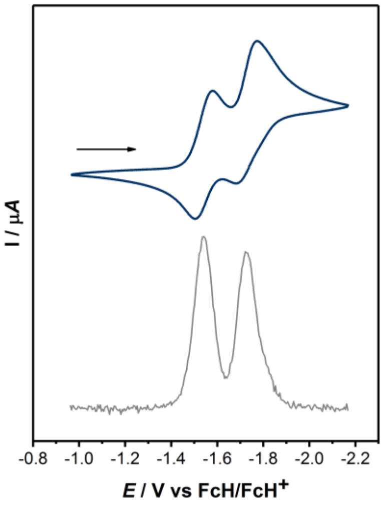

Potential	Peak-to-peak separation (ΔE)
$E_{1/2}$(1.Red) = −1.54 V	75.15 V
$E_{1/2}$(2.Red) = −1.73 V	91.09 V

Figure S8. Cyclic voltammogram (top) and differential potential voltammogram (bottom) of **3^Me^** in MeCN at 100 mV·s^{-1} (CV) and 20 mV·s-1 (dpv) with 0.1 M Bu_4NPF_6.

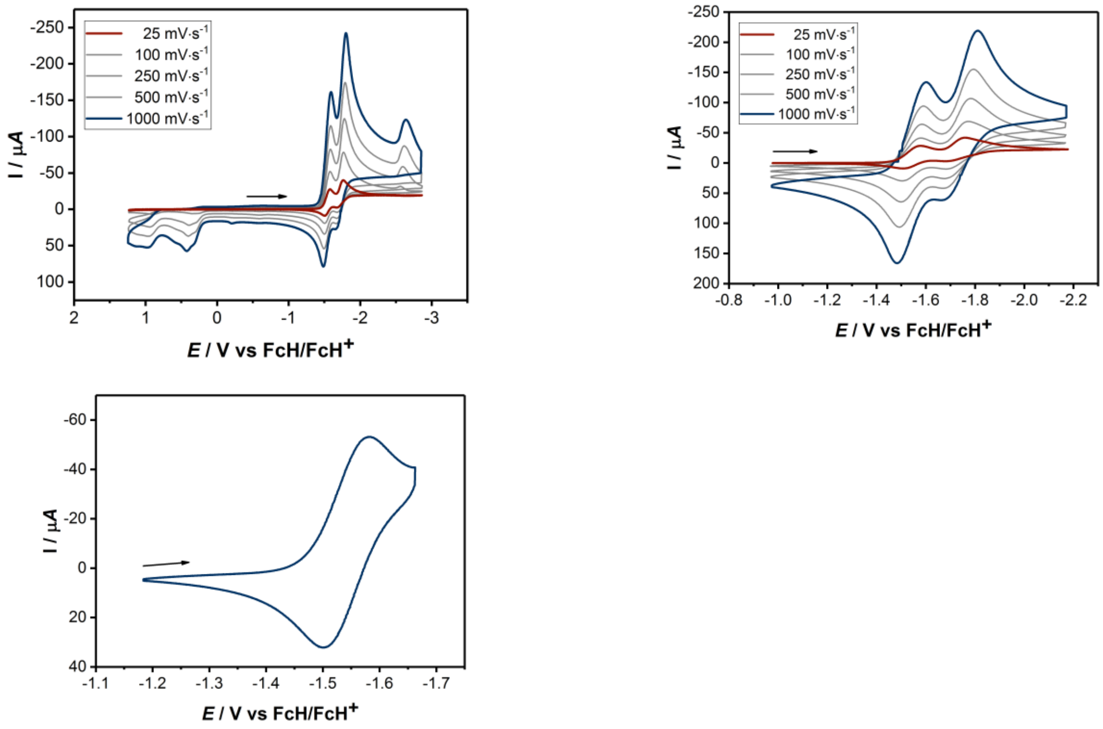

Figure S9. Cyclic voltammograms of **3^Me^** in MeCN at different scan rates with 0.1 M Bu_4NPF_6.

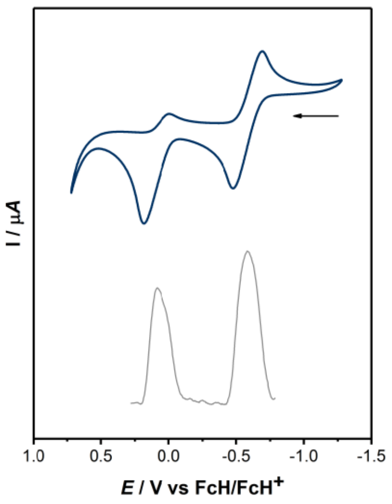

Potential	Peak-to-peak separation (ΔE)
$E_{1/2}$(1.Ox) = −0.58 V	213.16 V
$E_{1/2}$(2.Ox) = 0.09 V	187.39 V

Figure S10. Cyclic voltammogram (top) and differential potential voltammogram (bottom) of **6^H^** in THF at 100 mV·s^{-1} (CV) and 20 mV·s-1 (dpv) with 0.1 M Bu_4NPF_6.

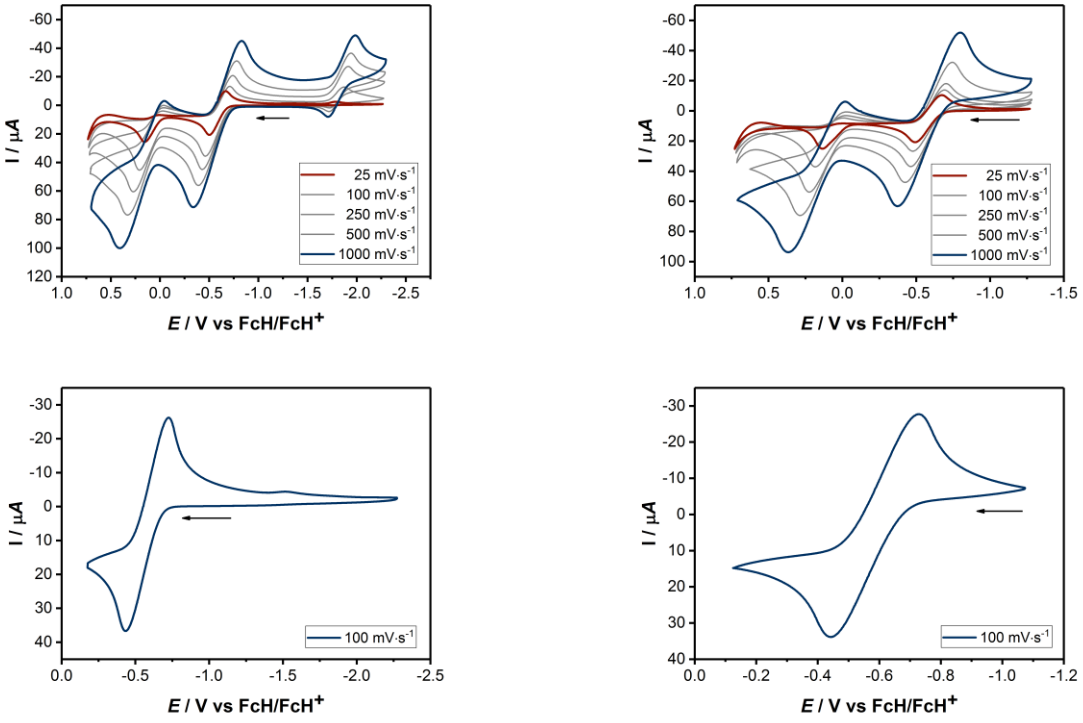

Figure S11. Cyclic voltammograms of **6^H^** in THF at different scan rates with 0.1 M Bu_4NPF_6.

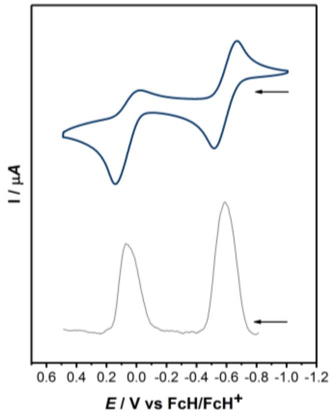

Potential	Peak-to-peak separation (ΔE)
$E_{1/2}$(1.Ox) = −0.59 V	151.82 V
$E_{1/2}$(2.Ox) = 0.06 V	167.16 V

Figure S12. Cyclic voltammogram (top) and differential potential voltammogram (bottom) of **6^{Me}** in THF at 100 mV·s^{-1} (CV) and 20 mV·s-1 (dpv) with 0.1 M Bu_4NPF_6.

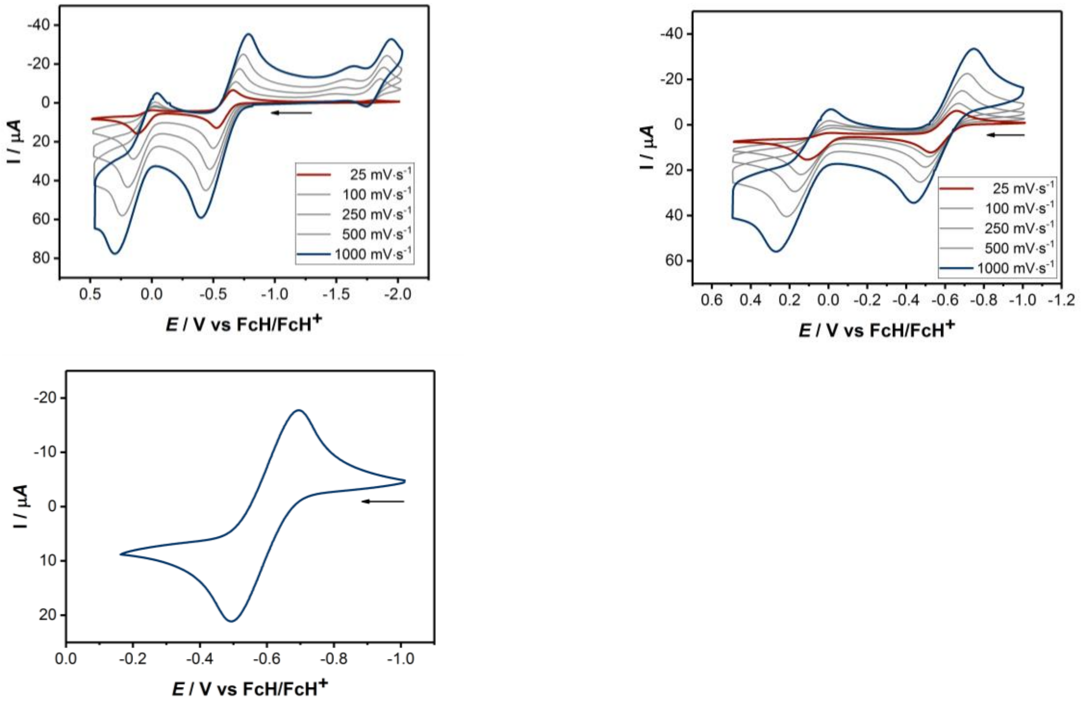

Figure S13. Cyclic voltammograms of **6^{Me}** in THF at different scan rates with 0.1 M Bu_4NPF_6.

Table S1. Redoxpotentials and peak-to-peak-separation of compounds 3^{H}, 3^{Me}, 6^{H} and 6^{Me}.

	Potential	Peak-to-peak separation (ΔE)	Solvent
**3^{H}**	$E_{1/2}$(1.Red) = −1.50 V	80.05 V	CH_3CN
	$E_{1/2}$(2.Red) = −1.71 V	82.19 V	
**3^{Me}**	$E_{1/2}$(1.Red) = −1.54 V	75.15 V	CH_3CN
	$E_{1/2}$(2.Red) = −1.73 V	91.09 V	
**6^{H}**	$E_{1/2}$(1.Ox) = −0.58 V	213.16 V	THF
	$E_{1/2}$(2.Ox) = 0.09 V	187.39 V	
**6^{Me}**	$E_{1/2}$(1.Ox) = −0.59 V	151.82 V	THF
	$E_{1/2}$(2.Ox) = 0.06 V	167.16 V	

UV/vis Spectroelectrochemistry

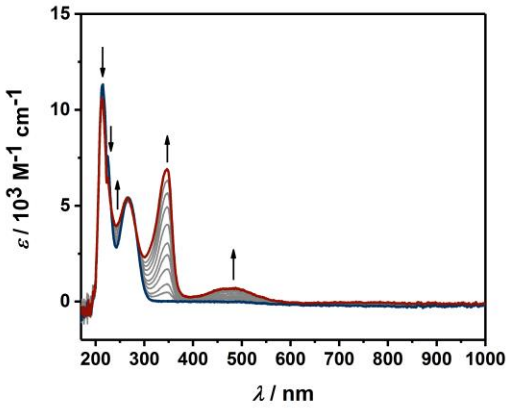

Native

Wavelength [nm]	Extinction coefficient [$M^{-1}\cdot cm^{-1}$]
214	11328
267	5395

Reduced

Wavelength [nm]	Extinction coefficient [$M^{-1}\cdot cm^{-1}$]
346	6846
478	711

Figure S14. Changes in UV/vis spectrum of $\mathbf{3^H}$ in MeCN with 0.1 M Bu_4NPF_6 during the first reduction.

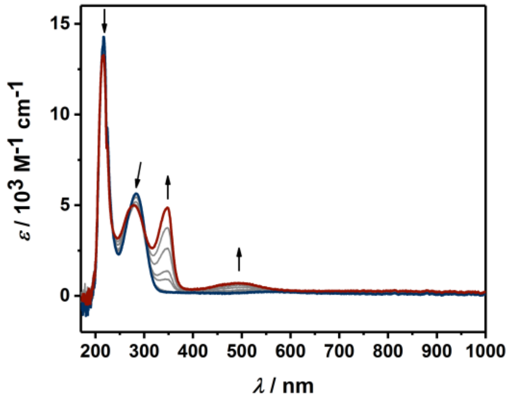

Native

Wavelength [nm]	Extinction coefficient [$M^{-1}\cdot cm^{-1}$]
217	14087
283	5645

Reduced

Wavelength [nm]	Extinction coefficient [$M^{-1}\cdot cm^{-1}$]
347	4866
492	705

Figure S15. Changes in UV/vis spectrum of $\mathbf{3^{Me}}$ in MeCN with 0.1 M Bu_4NPF_6 during the first reduction.

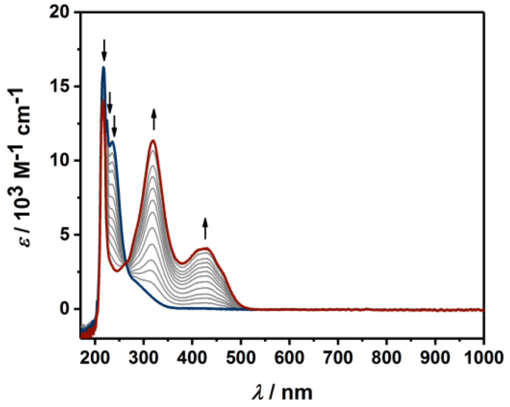

Native

Wavelength [nm]	Extinction coefficient [$M^{-1}\cdot cm^{-1}$]
217	16299
225	12721
235	11286

Oxidized

Wavelength [nm]	Extinction coefficient [$M^{-1}\cdot cm^{-1}$]
216	14050
320	11325
424	4039

Figure S16. Changes in UV/vis spectrum of $\mathbf{6^H}$ in THF with 0.1 M Bu_4NPF_6 during the first oxidation.

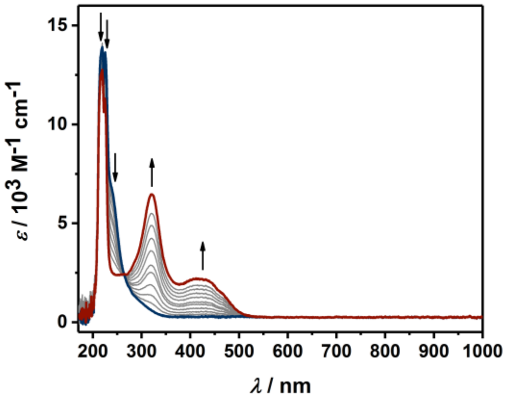

Native

Wavelength [nm]	Extinction coefficient [$M^{-1}\cdot cm^{-1}$]
219	13910
225	13645

Oxidized

Wavelength [nm]	Extinction coefficient [$M^{-1}\cdot cm^{-1}$]
321	6451
420	2176

Figure S17. Changes in UV/vis spectrum of **6**$^{\mathbf{Me}}$ in THF with 0.1 M Bu_4NPF_6 during the first oxidation.

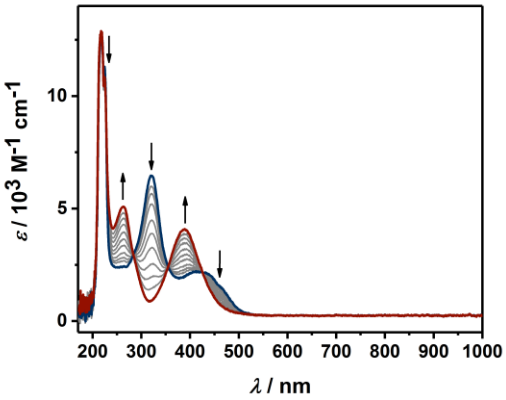

Wavelength [nm]	Extinction coefficient [$M^{-1}\cdot cm^{-1}$]
262	5086
389	4082

Figure S18. Changes in UV/vis spectrum of **6**$^{\mathbf{Me}}$ in THF with 0.1 M Bu_4NPF_6 during the second oxidation.

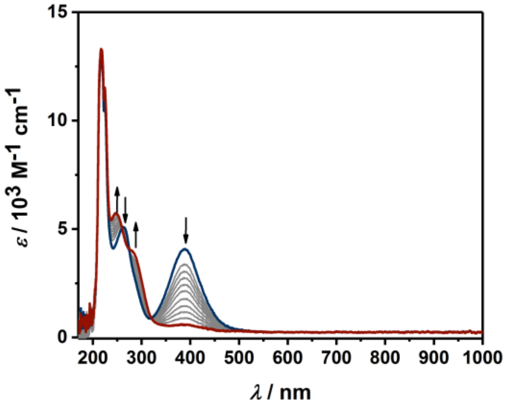

Wavelength [nm]	Extinction coefficient [$M^{-1}\cdot cm^{-1}$]
247	5737

Figure S19. Further changes in UV/vis spectrum of **6**$^{\mathbf{Me}}$ in THF with 0.1 M Bu_4NPF_6 after longer electrolysis at the second oxidation potential.

Electrochemical Study of Bistability

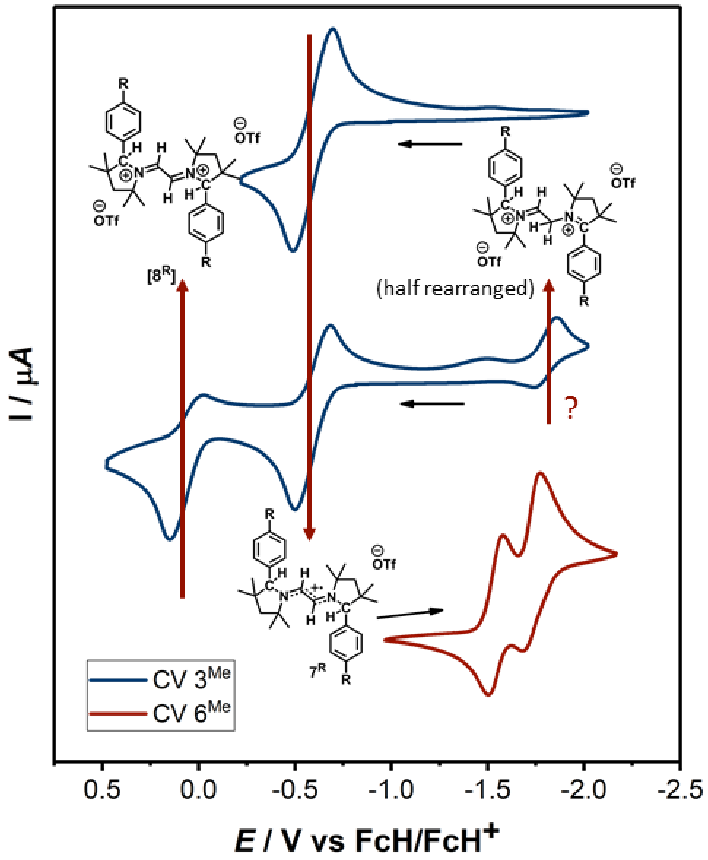

Figure S20. Cyclic voltammogram scheme for clarification of the bistability of $\mathbf{3^{Me}}$ and $\mathbf{6^{Me}}$.

In order to study the rearrangement reactions of $\mathbf{3^{Me}}$ and $\mathbf{6^{Me}}$, we performed cyclic voltammetric studies at different scan rates and combined cyclic voltammetry and chronoamperometry experiments.

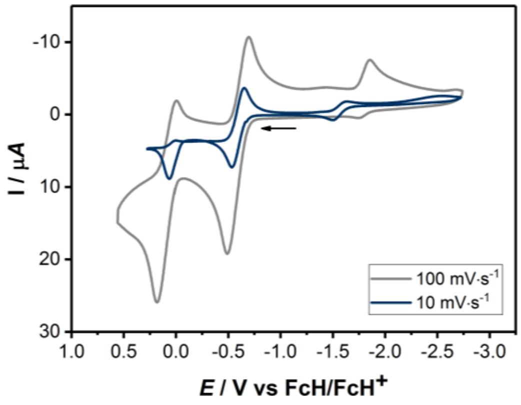

	100 m·Vs^{-1}	10 m·Vs^{-1}
$E_{1/2}$(1.Ox)	–0.58 V	–0.59 V
$E_{1/2}$(1.Red)	–1.80 V	–1.57 V

Figure S21. Changes of the half-wave potential of the first oxidation in the cyclic voltammograms of $\mathbf{6^{Me}}$ in THF at 100 mV·s^{-1} and 10 mV·s^{-1} with 0.1 M Bu_4NPF_6.

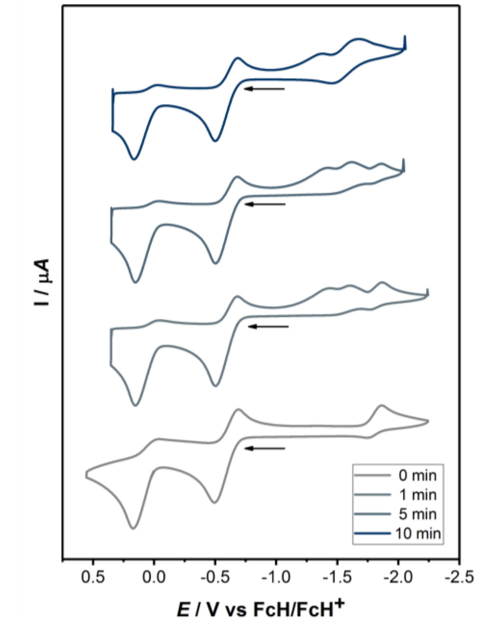

Figure S22. Combined cyclic voltammograms and chronoamperometry of **6^{Me}** in THF at 100 mV·s^{-1} with 0.1 M Bu_4NPF_6. The Chronoamperometry was performed at 0.35 V for the times listed in the legend.

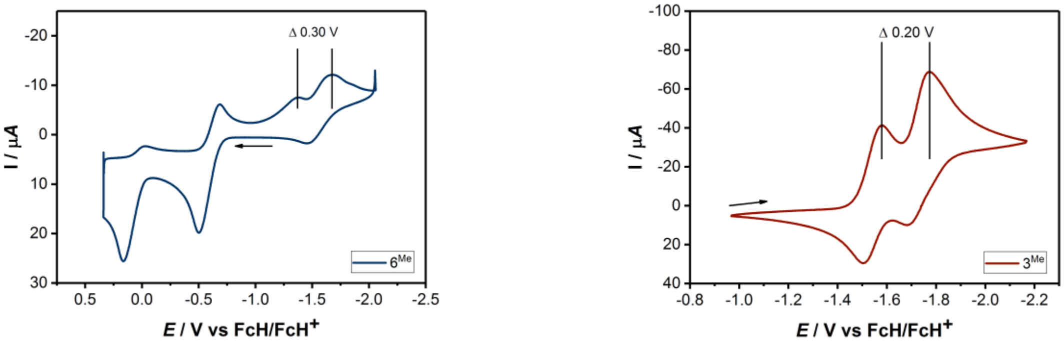

Figure S23. Cyclic voltammograms of **6^{Me}** (right) in THF and **3^{Me}** (left) in CH_3CN at 100 mV·s^{-1} with listed peak-forward potential separations of the first and second oxidation.

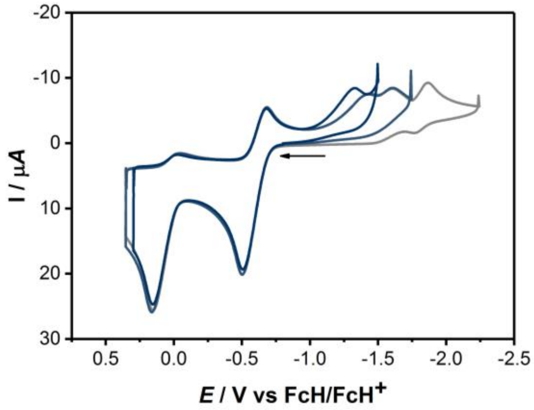

Figure S24. Combined cyclic voltammograms and chronoamperometry of $\mathbf{6^{Me}}$ in THF at 100 mV·s^{-1} with 0.1 M Bu_4NPF_6. The Chronoamperometry was performed at 0.35 V for 1 min.

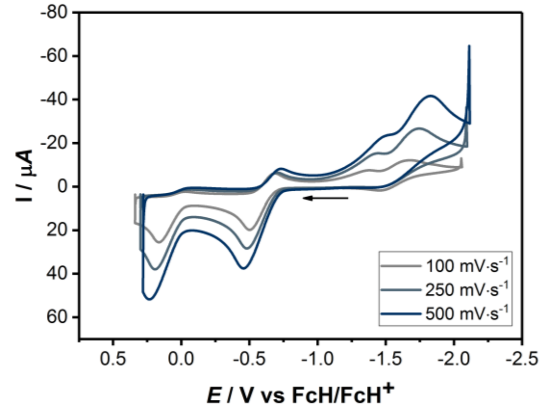

Figure S25. Combined cyclic voltammograms and chronoamperometry of $\mathbf{6^{Me}}$ in THF at different scan rates with 0.1 M Bu_4NPF_6. The Chronoamperometry was performed at 0.35 V for 10 min.

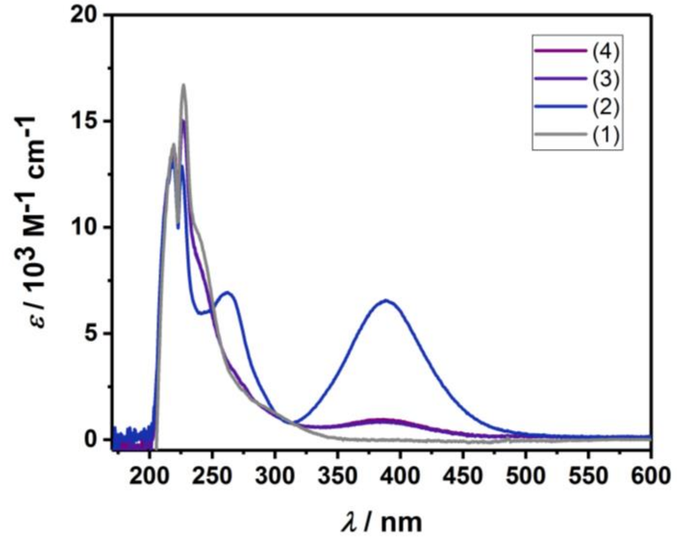

Figure S26. Changes in the UV/vis spectra of $\mathbf{6^{Me}}$ with (1) the native spectrum, (2) the spectrum after 10 min of chronoamperometry at the second oxidation potential, (3) spectrum at the potential corresponding to the second reduction which appeared after the chronoamperommetry, and (4) final spectrum at 0 V versus the silver reference electrode. Spectrum 2 corresponds to the final spectrum in Figure S18.

EPR Spectroelectrochemistry

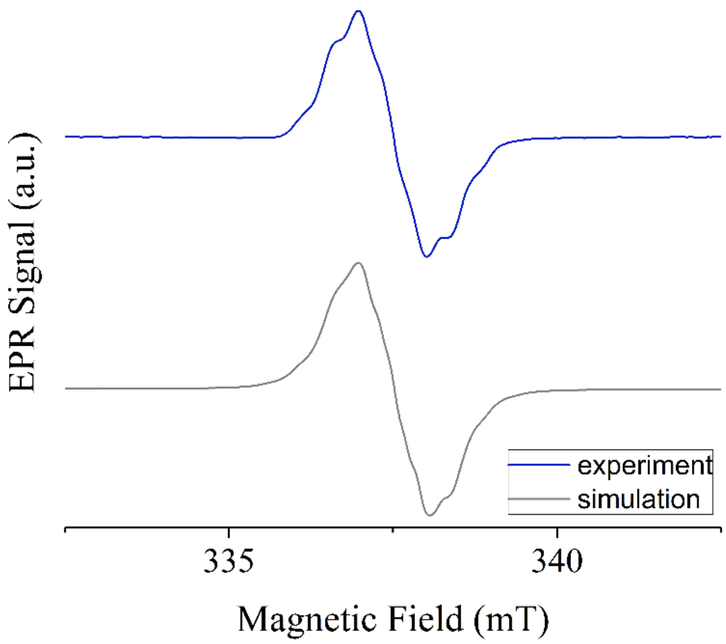

Figure S27. EPR spectrum of electrochemically generated **4^H** by one-electron reduction at −50 °C, together with simulation.

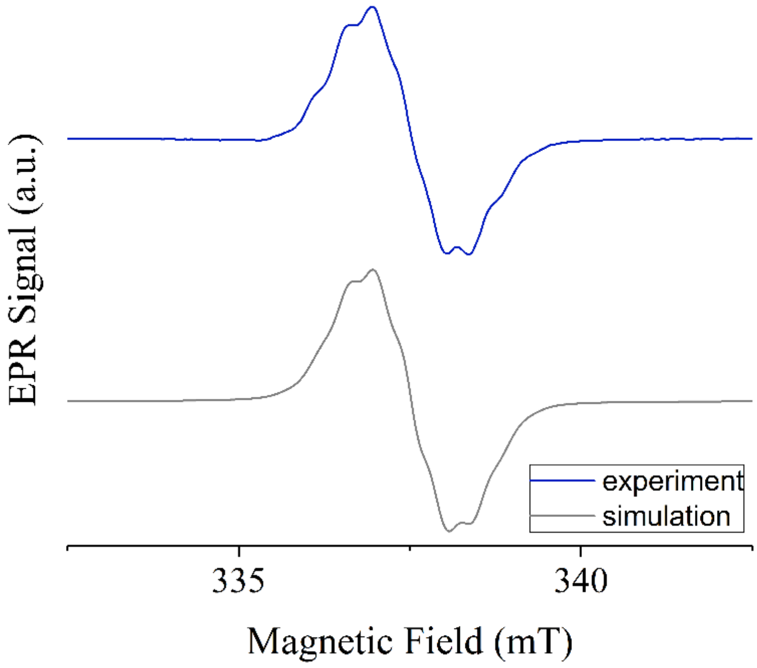

Figure S28. EPR spectrum of electrochemically generated **4^Me** by one-electron reduction at −50 °C, together with simulation.

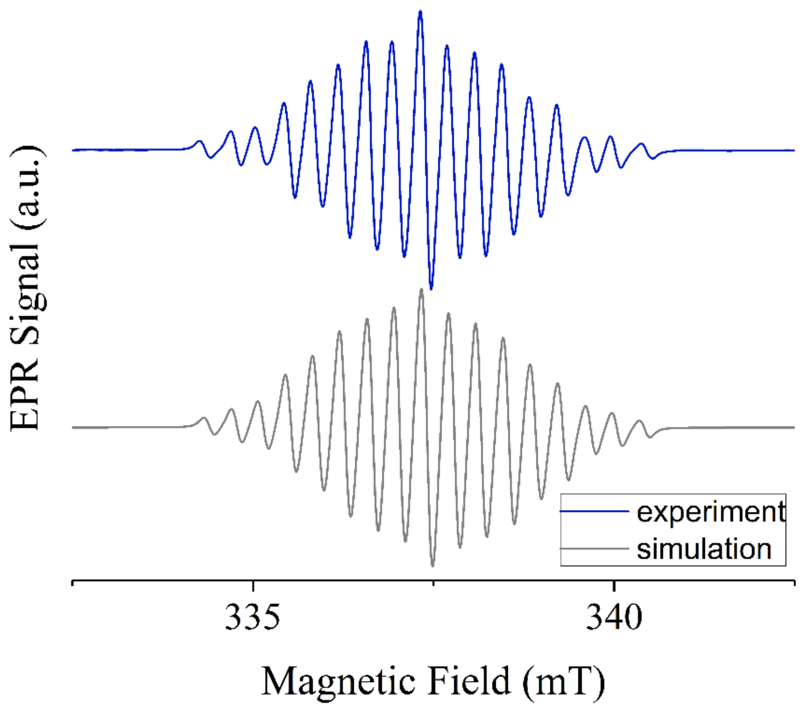

Figure S29. EPR spectrum of electrochemically generated **7^H^** by one-electron oxidation at −5 °C, together with simulation.

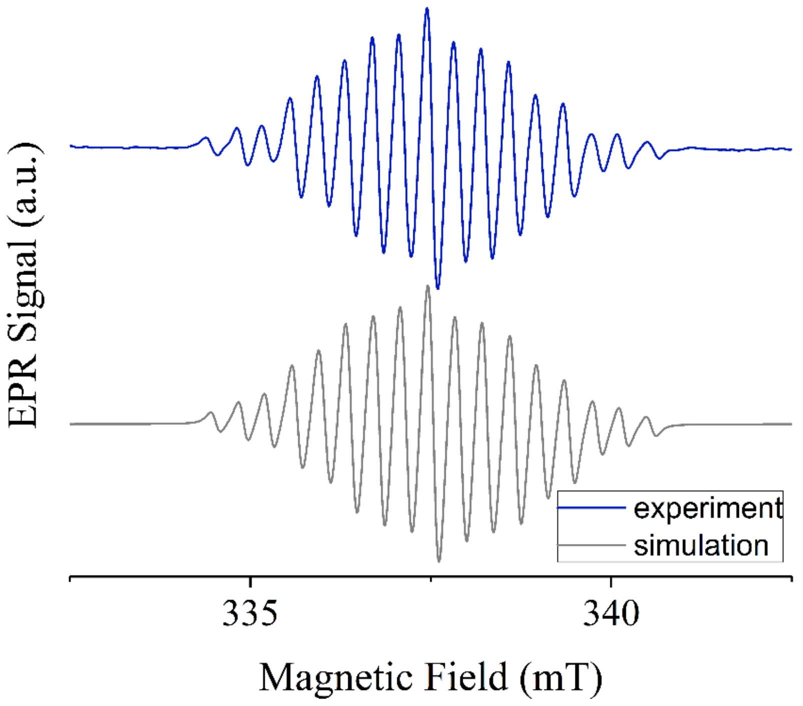

Figure S30. EPR spectrum of electrochemically generated $\mathbf{7^{Me}}$ by one-electron oxidation at −5 °C, together with simulation.

Table S2. EPR parameters obtained by least-squares fitting of simulations to the experimentally obtained EPR spectra shown in Figures 27-30, together with parameters calculated by DFT methods.

	**7^{H}**	Calculated		**7^{Me}**	Calculated	
Temperature	−5 °C			−5 °C		
g-value	2.0037	2.00328		2.0035	2.00327	
A(^{14}N) (2) (MHz)	20.7347	0N: 17.02	36N: 16.89	20.5546	0N: 15.6	40N: 18.24
A(^{1}H) (2) (MHz)	32.2998	4H: 40.80	40H: 39.33	32.7305	4H: 35	44H: 48.4
A(^{1}H) (2) (MHz)	10.7586	2H: −10.83	38H: −11.82	10.7204	2H: −9.35	42H: −6.11
Linewidth (Gaussian)	0.1042 mT			0.0689 mT		
Linewidth (Lorentzian)	0.0842 mT			0.1108 mT		
	**4^{H}**			**4^{Me}**		
	−50 °C			−50 °C		
g-values	g⊥ = 2.0027			g⊥ = 2.0026		
	g|| = 2.0031			g|| = 2.0028		
A-values	A_{iso} (MHz)	Axial Distortion (AD, MHz)[1]		A_{iso} (MHz)	Axial Distortion (AD, MHz)[1]	
^{14}N	11.9511	1.9330		11.7488	1.1859	
^{1}H	10.5648	2.0013		13.7131	0.6593	
^{1}H	6.0679	3.3648		9.1945	0.3839	
^{1}H	6.0872	3.5785		9.5955	1.0154	
^{1}H	5.4997	0.9273		7.0222	3.8265	
^{1}H	5.2569	3.4587		7.0000	0	
^{1}H	10.6312	1.3058		10.9273	1.6801	
Linewidth (Gaussian)	0.1286 mT			0.2043 mT		
Linewidth (Lorentzian)	0.1619 mT			0.1543 mT		

[1] $A_{\perp} = A_{iso} - AD$, $A_{||} = A_{iso} + 2 \times AD$

DFT Calculations

Table S3. Gibbs Free Enthalpies of formation, charge and total spin of species studied in this work.

			R = H		R = Me	
	Charge	Spin	Total Thermal Energy[a] (Ha)	Gibbs Enthalpy (Ha)	Total Thermal Energy[a] (Ha)	Gibbs Enthalpy (Ha)
**3^R**	+2	0	-1276.01316775	-1276.09520782	-1354.52365072	-1354.61099294
**4^R**	+1	1/2	-1276.13763639	-1276.22211089	-1354.65796354	-1354.74829535
**5^R**	0	1	-1276.25535927	-1276.33992775	-1354.75598473	-1354.84302825
**6^R**	0	0	-1276.30543079	-1276.38445572	-1354.81063459	-1354.89690572
**7^R**	+1	1/2	-1276.17067596	-1276.25629867	-1354.68069469	-1354.76971789
**8^R**	+2	0	-1275.97868929	-1276.05999785	-1354.47793558	-1354.56660942

[a]Electronic Energy plus Zero-Point Energy and Thermal Vibrational, Rotational and Translational Corrections.

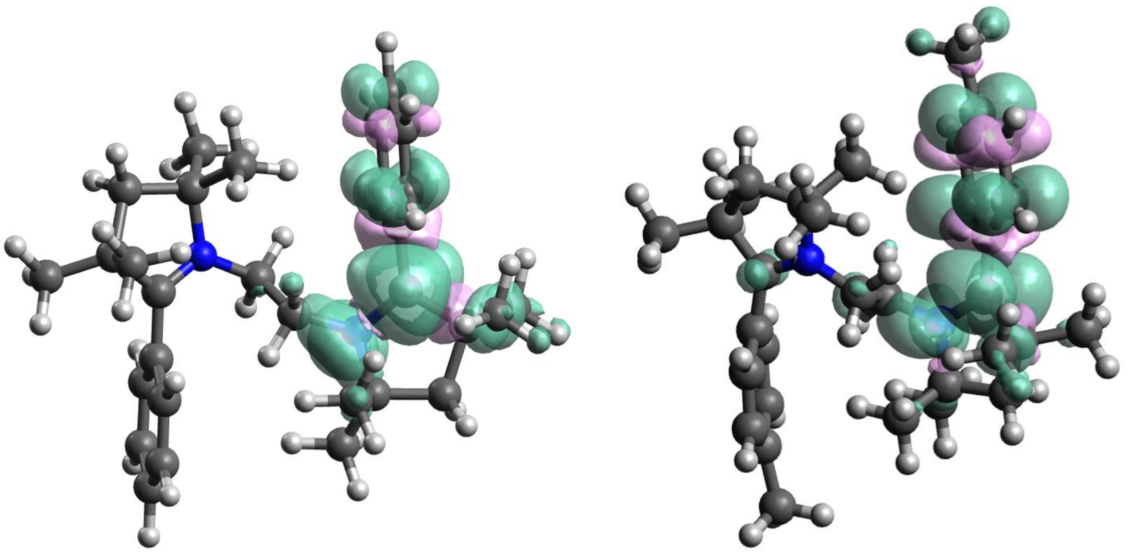

Figure S31. Spin density of **4^H** (left) and **4^{Me}** (right).

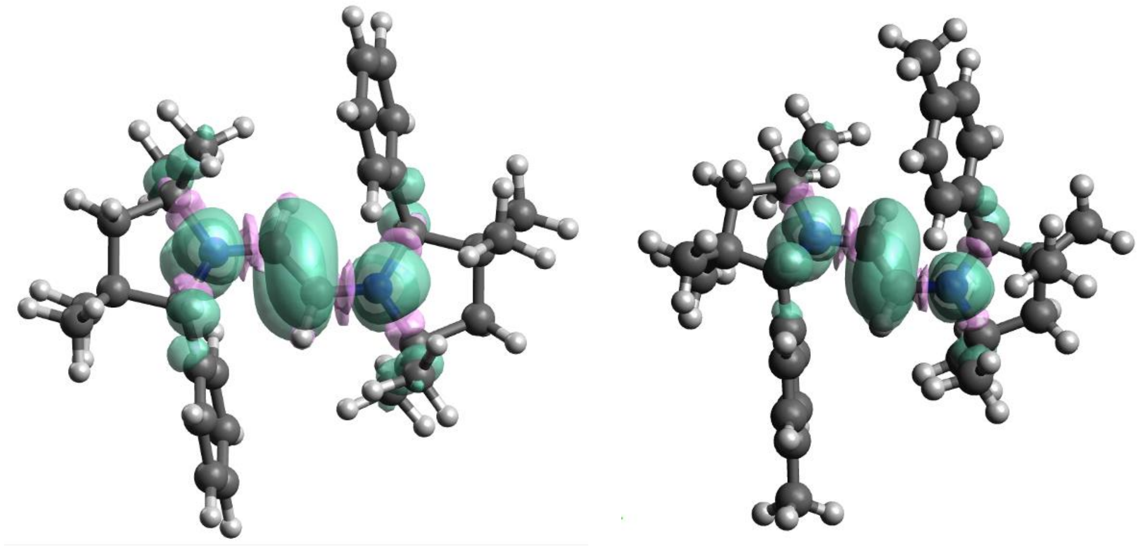

Figure S32. Spin density of 7^{H} (left) and 7^{Me} (right).

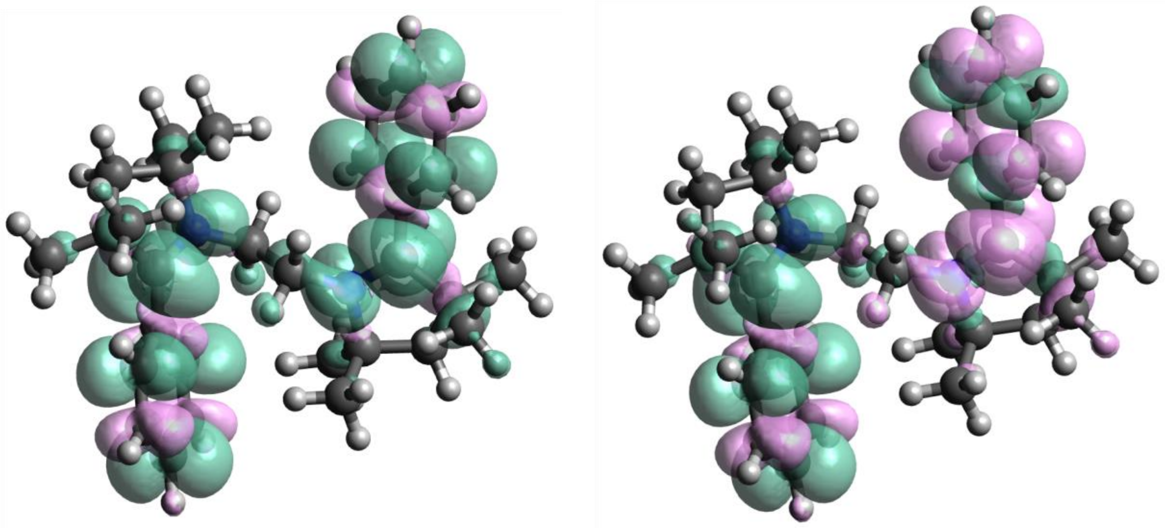

Figure S33. Spin-density of species 5^{H}, corresponding to the two electron reduced form of 3^{H} in the triplet (left) and singlet (right) energy states. Singlet-triplet gap ≈ 26 cm^{-1} (antiferromagnetic).

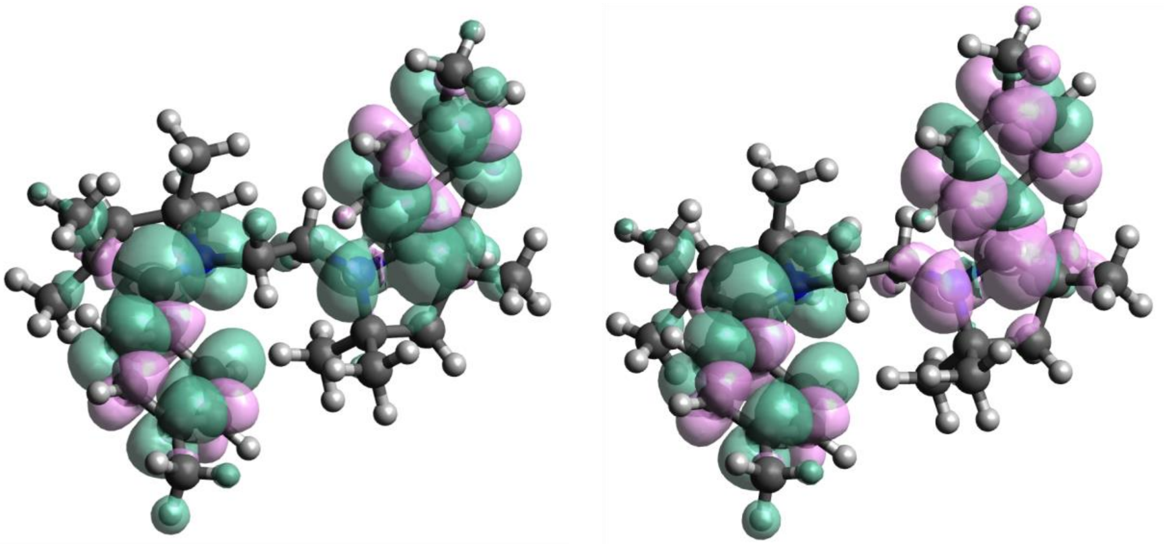

Figure S34. Spin-density of species **5^{Me}**, corresponding to the two electron reduced form of **3^{Me}** in the triplet (left) and singlet (right) energy states. Singlet-triplet energy is J = +0.39 cm^{-1} (ferromagnetic).

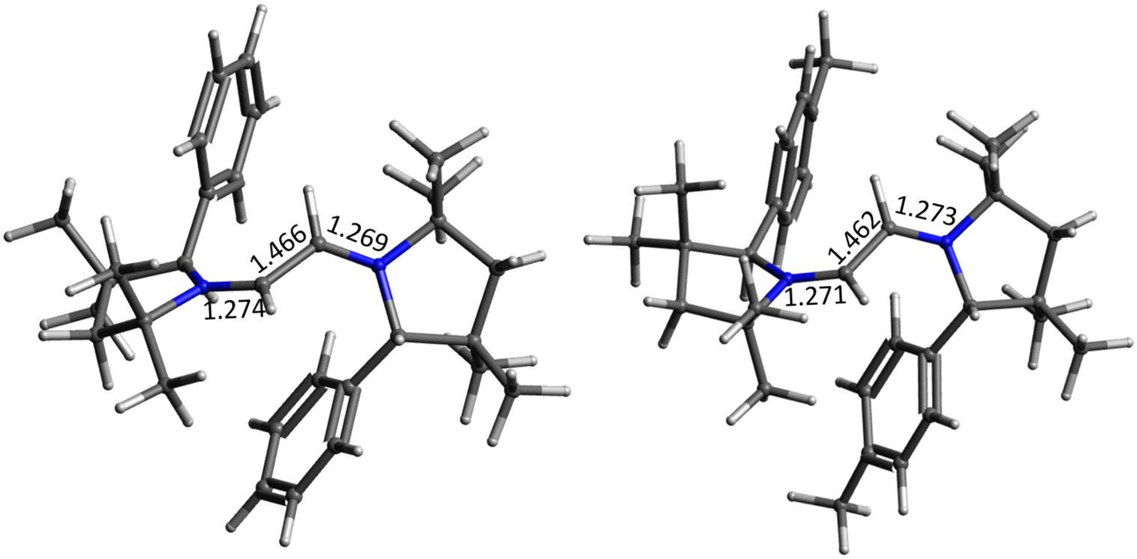

Figure S35. Optimized structures of **8^{H}** (left) and **8^{Me}** (right), showing the bond distances for the N=C(H)-C(H)=N region.

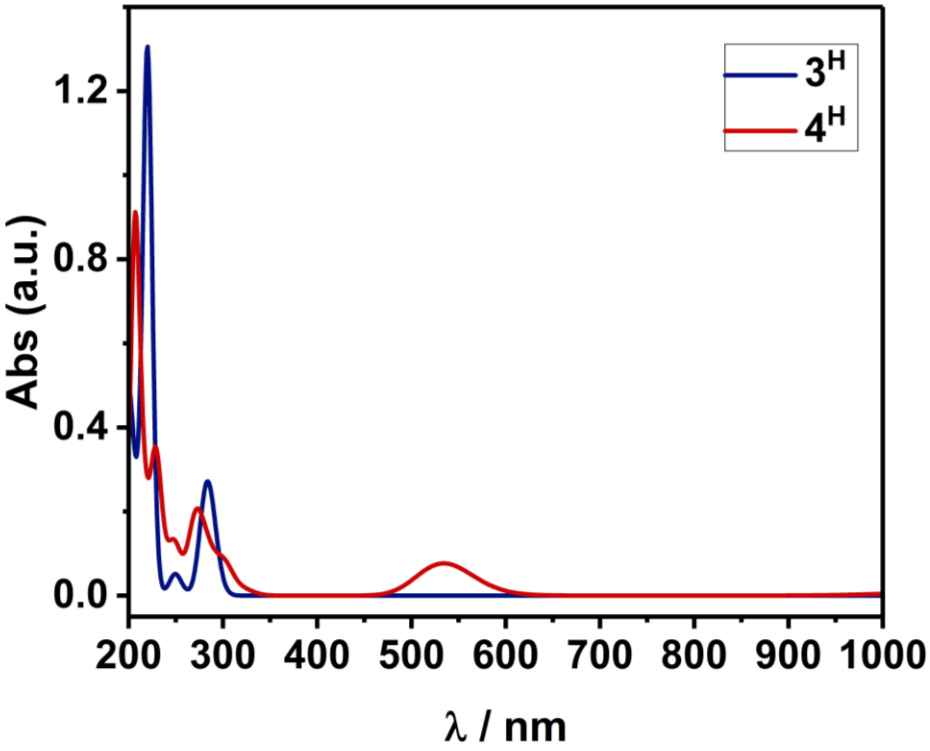

Figure S36. Calculated UV-vis-NIR spectra for $\mathbf{3^H}$ and $\mathbf{4^H}$ (one electron reduced form of $\mathbf{3^H}$), using time-dependent DFT (TDDFT), with the PBE0 functional and def2-TZVP basis sets.

Table S4. Experimental absorption positions and extinction coefficients, and strongest TDDFT calculated transitions. Selected density differences (difference between squared orbitals) for representative transitions have been plot in Figures S37-S42.

$\mathbf{3^H}$			$\mathbf{4^H}$		
l (nm), e (M^{-1} cm^{-1}) (experimental)	l (nm), oscillator strength (calculated)	Orbitals involved in transitions	l (nm), e (M^{-1} cm^{-1}) (experimental)	l (nm), oscillator strength (calculated)	Orbitals involved in transitions
214 (11328)	220.1 (0.293)	0.46 (112b-> 117b)<br>0.46 (112a-> 117a)<br>0.01 (110b-> 117b)	346 (6846)	270.2 (0.012)	0.43 (113a-> 118a)<br>0.42 (112b-> 118b)<br>0.11 (113b-> 118b)
				271.5 (0.021)	0.46 (113b-> 117b)<br>0.36 (114a-> 118a)<br>0.13 (112b-> 118b)
				281.1 (0.012)	0.85 (117a-> 126a)<br>0.05 (117a-> 125a)<br>0.03 (117a-> 128a)
				301.1 (0.015)	0.91 (116b-> 117b)<br>0.06 (114b-> 117b)<br>0.03 (115b-> 117b)
267 (5395)	283.0 (0.044)	0.25 (113a-> 117a)<br>0.25 (113b-> 117b)<br>0.11 (115b-> 117b)	478 (711)	531.3 (0.009)	0.80 (117a-> 120a)<br>0.17 (117a-> 121a)<br>0.04 (117a-> 122a)
	285.6 (0.020)	0.21 (115b-> 117b)<br>0.21 (115a-> 117a)		536.4 (0.010)	0.81 (117a-> 121a)<br>0.18 (117a-> 120a)

		0.21 (116a-> 117a)			0.01 (117a-> 119a)

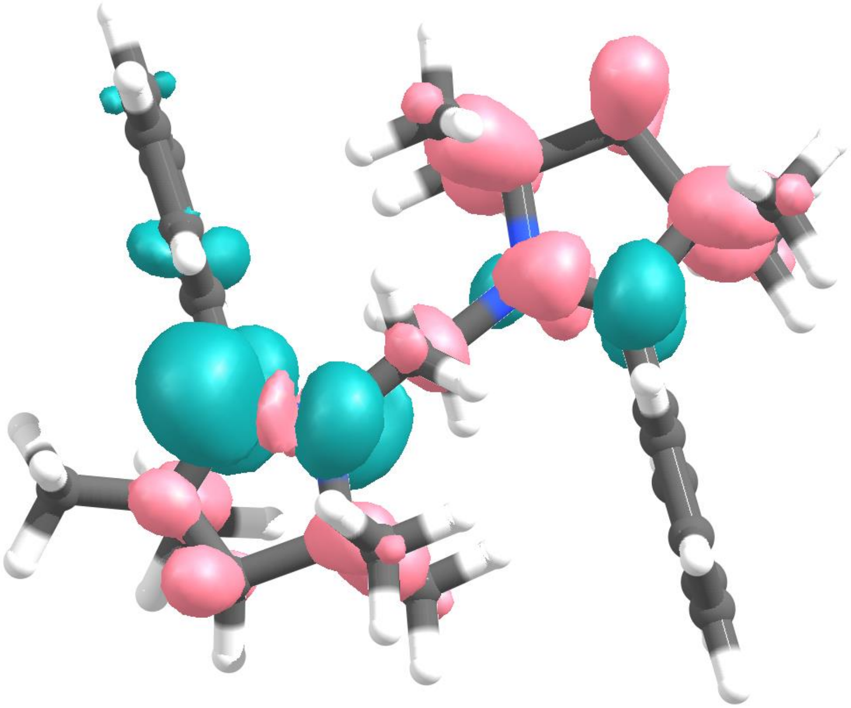

Figure S37. Density difference between the 112a (HOMO–5a) and 117a (LUMOa) orbitals for **3^{H}**, corresponding to the 220.1 nm transition. Pink and cyan colors indicate regions of negative and positive density, respectively.

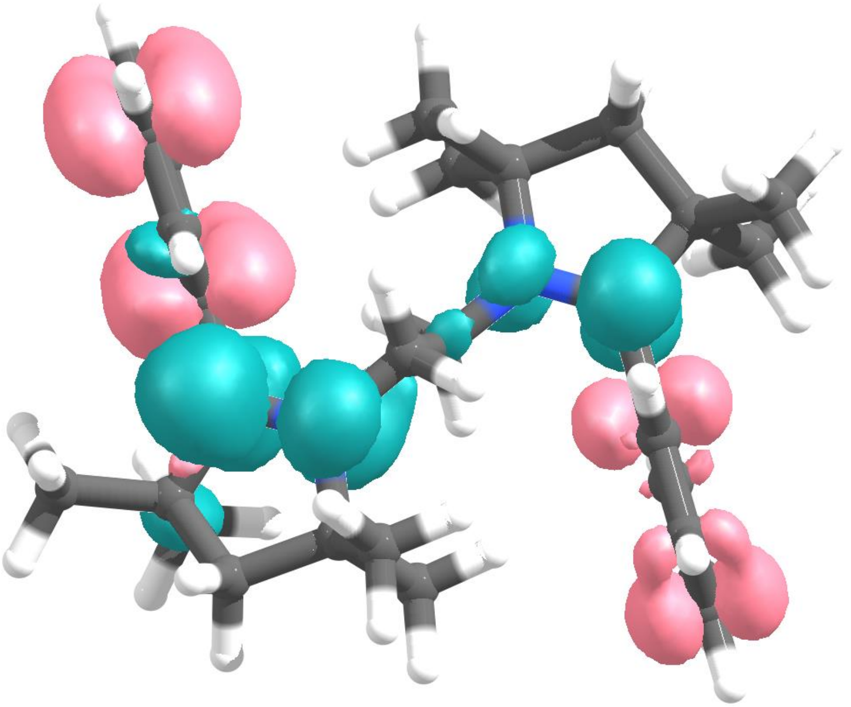

Figure S38. Density difference between the 113a (HOMO–4a) and 117a (LUMOa) orbitals for **3^{H}**, corresponding to the 283.0 nm transition.

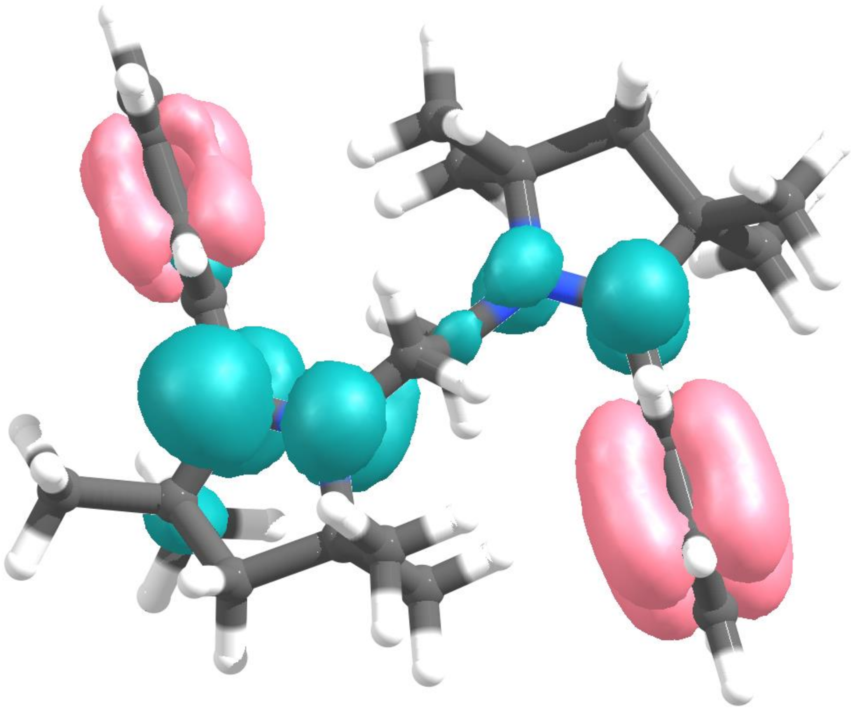

Figure S39. Density difference between the 116a (HOMOa) and 117a (LUMOa) orbitals for **3^H**, corresponding to the 285.6 nm transition.

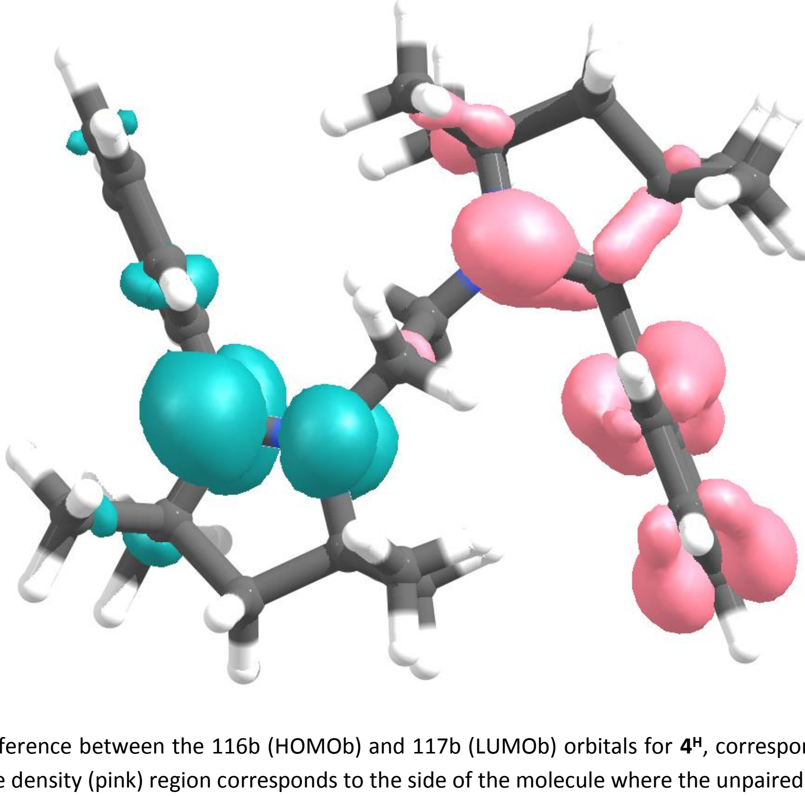

Figure S40. Density difference between the 116b (HOMOb) and 117b (LUMOb) orbitals for **4^H**, corresponding to the 301.1 nm transition. The negative density (pink) region corresponds to the side of the molecule where the unpaired spin is located.

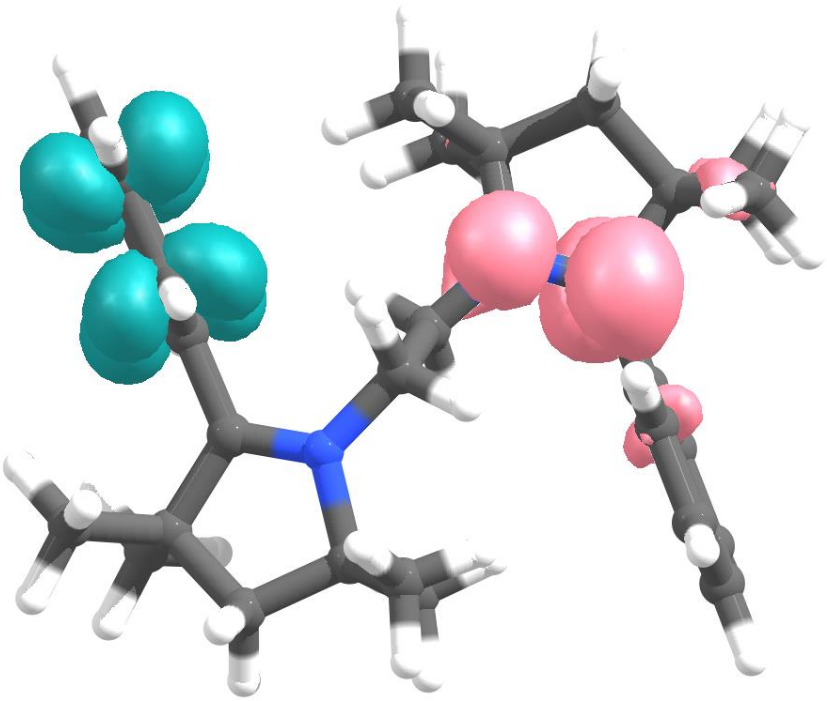

Figure S41. Density difference between the 117a (HOMOa) and 120a (LUMO+2a) orbitals for **4^{H}**, corresponding to the 531.3 nm transition. The transitions depicted in this Figure as well as Figure S42 are originated in the magnetic orbital.

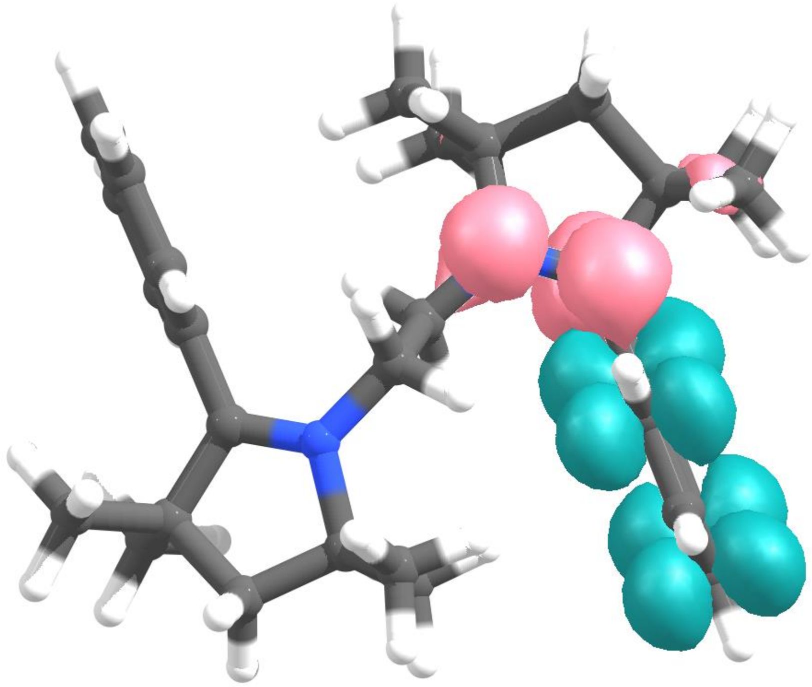

Figure S42. Density difference between the 117a (HOMOa) and 121a (LUMO+3a) orbitals for **4^{H}**, corresponding to the 536.4 nm transition.

Coordinates of optimized geometries of compounds from **Table S3**

3^H^

C	-1.947946	0.301505	-0.999606
C	-0.412562	0.246200	-1.096682
H	-2.333097	-0.706505	-0.843502
H	-2.378884	0.682409	-1.923480
C	-2.310161	1.196048	0.208831
C	0.168005	-1.160491	-1.125594
N	-0.022642	0.892985	0.186097
C	0.147363	1.057107	-2.254052
C	-0.986462	1.371417	0.880599
C	-3.347142	0.573917	1.141378
C	1.369328	0.975068	0.553675
H	-3.073628	-0.444727	1.423281
H	-4.303393	0.537543	0.617072
H	-3.470874	1.173612	2.045287
C	-2.802535	2.593526	-0.185737
C	-0.758101	2.055272	2.164821
H	-2.102650	3.105834	-0.848534
H	-2.960309	3.205923	0.703776
H	-3.757591	2.487785	-0.702531
C	-0.576712	3.438109	2.182785
C	-0.374050	4.080981	3.396796
C	-0.334413	3.344909	4.571719
C	-0.494496	1.964974	4.547390
C	-0.704444	1.311161	3.341993
H	-0.586815	4.000084	1.258381
H	-0.231137	5.154554	3.414841
H	-0.174114	3.845003	5.519113
H	-0.450172	1.397049	5.468262
H	-0.843880	0.236258	3.322105
H	-0.108634	-1.732287	-0.239116
H	1.254180	-1.134042	-1.219977
H	-0.221227	-1.672420	-2.006340
H	-0.192038	0.593480	-3.180842
H	1.238207	1.065277	-2.270182
H	-0.214065	2.086440	-2.228293
C	1.786141	-0.150641	1.499688
H	1.547645	1.945695	1.006467
H	1.953297	0.923011	-0.360465
H	1.228959	-0.119879	2.430796
N	3.184755	0.076427	1.784602
H	1.651920	-1.134814	1.060632
C	4.125098	-0.127750	0.928901
C	5.372632	0.603614	1.319555
C	3.921278	-0.910743	-0.297700
C	5.131773	0.799329	2.824921
C	5.335069	1.931757	0.532958
C	6.649414	-0.141063	0.983310
C	3.608587	0.789663	3.028204
H	5.581232	1.717221	3.199074
H	5.567300	-0.047764	3.358635
C	2.967272	2.172166	3.101511
C	3.160645	-0.023381	4.229381
H	3.212571	2.806582	2.251504
H	3.334072	2.656396	4.007253
H	1.883959	2.097385	3.199382

H	3.525410	-1.050497	4.172583
H	2.073401	-0.037597	4.325906
H	3.565824	0.440364	5.129431
H	6.674383	-1.121495	1.463163
H	7.510043	0.435734	1.324713
H	6.740592	-0.272493	-0.097162
H	4.409283	2.484928	0.679438
H	5.466337	1.760442	-0.535422
H	6.170680	2.543983	0.877378
C	3.853959	-0.277335	-1.537578
C	3.799153	-2.301399	-0.207860
H	3.843805	-2.784595	0.761018
C	3.633432	-3.053920	-1.363289
C	3.562940	-2.419471	-2.596613
H	3.552206	-4.132538	-1.298966
H	3.421820	-3.002617	-3.498444
C	3.659467	-1.036516	-2.680133
H	3.925262	0.799767	-1.611458
H	3.584890	-0.545616	-3.642277

4^{H}

C	-2.107217	0.197183	-0.907376
C	-0.588167	0.173371	-1.104274
H	-2.435765	-0.789812	-0.573202
H	-2.624619	0.415815	-1.842921
C	-2.415029	1.240549	0.188744
C	-0.083786	-1.223590	-1.433188
N	-0.123479	0.613783	0.225301
C	-0.157054	1.158304	-2.190849
C	-1.042438	1.514951	0.757333
C	-3.348466	0.634100	1.239729
C	1.281417	0.832760	0.434023
H	-2.894022	-0.247528	1.699372
H	-4.292045	0.328605	0.776839
H	-3.571751	1.354772	2.030517
C	-3.058455	2.509214	-0.369014
C	-0.816347	2.121032	2.078755
H	-2.440524	2.961532	-1.146625
H	-3.196072	3.246091	0.427296
H	-4.040019	2.283336	-0.797986
C	-0.701197	3.507185	2.212839
C	-0.508468	4.094395	3.456376
C	-0.426687	3.305886	4.596025
C	-0.543203	1.925833	4.484483
C	-0.737001	1.342439	3.240904
H	-0.747903	4.124784	1.322857
H	-0.410258	5.171562	3.533346
H	-0.271574	3.763707	5.566330
H	-0.489994	1.301761	5.369765
H	-0.849775	0.265967	3.166554
H	-0.306838	-1.914732	-0.617757
H	0.993514	-1.239006	-1.624761
H	-0.575714	-1.586952	-2.338465
H	-0.639169	0.901868	-3.136633
H	0.923834	1.130087	-2.352675
H	-0.440278	2.177915	-1.921441
C	1.838576	-0.128503	1.479238
H	1.469840	1.863781	0.747066

H	1.817091	0.672579	-0.501155
H	1.283802	-0.038176	2.407803
N	3.239959	0.159809	1.774257
H	1.756839	-1.155605	1.125413
C	4.211941	-0.048372	0.960015
C	5.527685	0.434556	1.493792
C	4.044266	-0.721104	-0.338084
C	5.174352	0.748453	2.955968
C	5.971160	1.678805	0.716330
C	6.583581	-0.660934	1.377728
C	3.646785	0.843364	3.047903
H	5.648002	1.666003	3.301399
H	5.521318	-0.067192	3.592041
C	3.126874	2.274295	3.049637
C	3.111120	0.089507	4.250537
H	3.443005	2.823436	2.161539
H	3.534707	2.778325	3.926756
H	2.037800	2.311804	3.125396
H	3.353219	-0.973201	4.190089
H	2.033644	0.215517	4.370022
H	3.591518	0.500667	5.139878
H	6.256386	-1.576303	1.875117
H	7.500958	-0.317548	1.859279
H	6.806581	-0.889722	0.333771
H	5.220124	2.469734	0.751864
H	6.186686	1.440490	-0.326227
H	6.886404	2.057459	1.175640
C	4.092178	0.000607	-1.529804
C	3.874339	-2.103904	-0.362857
H	3.848606	-2.665384	0.564084
C	3.743407	-2.758407	-1.578487
C	3.776330	-2.039325	-2.765141
H	3.608566	-3.833124	-1.595436
H	3.665162	-2.552527	-3.712988
C	3.945803	-0.661722	-2.738864
H	4.218885	1.076000	-1.515013
H	3.966467	-0.095012	-3.661957

5^H

C	-2.198683	0.102395	-0.609043
C	-0.714812	-0.267246	-0.644570
H	-2.721603	-0.600718	0.045769
H	-2.652908	0.038231	-1.599472
C	-2.308447	1.504672	0.006317
C	-0.533003	-1.759158	-0.403838
N	-0.183946	0.534412	0.482247
C	-0.093739	0.077865	-1.998922
C	-1.092969	1.487453	0.903428
C	-3.628573	1.632649	0.758919
C	1.246086	0.735011	0.660694
H	-3.701788	0.884283	1.551095
H	-4.459809	1.479805	0.064271
H	-3.744361	2.620132	1.209778
C	-2.249977	2.618660	-1.049569
C	-0.970315	2.181976	2.143354
H	-1.278537	2.673950	-1.540291
H	-2.449170	3.589783	-0.590587
H	-3.011920	2.452194	-1.817089

C	-1.422161	3.514044	2.297091
C	-1.306878	4.177238	3.503391
C	-0.750973	3.549935	4.617359
C	-0.337502	2.225259	4.500196
C	-0.444110	1.556493	3.296574
H	-1.830418	4.037958	1.441561
H	-1.649250	5.204330	3.578428
H	-0.656621	4.078123	5.558704
H	0.053187	1.701102	5.366316
H	-0.188862	0.506221	3.246108
H	-0.889121	-2.039573	0.589445
H	0.511358	-2.056569	-0.509013
H	-1.107966	-2.317022	-1.147609
H	-0.617787	-0.487247	-2.772227
H	0.958888	-0.208963	-2.048398
H	-0.188036	1.137131	-2.236289
C	1.913488	-0.242469	1.630016
H	1.405853	1.751572	1.027956
H	1.738872	0.657785	-0.310710
H	1.482937	-0.096706	2.616145
N	3.339858	-0.044980	1.780718
H	1.669278	-1.266205	1.340242
C	4.276365	0.008939	0.777938
C	5.546312	0.651833	1.308657
C	4.090396	-0.532347	-0.521578
C	5.368738	0.428659	2.817980
C	5.647607	2.146841	0.974907
C	6.834196	-0.034954	0.861774
C	3.869516	0.355256	3.097300
H	5.855564	1.204372	3.410745
H	5.823553	-0.529076	3.085003
C	3.312663	1.698740	3.566416
C	3.569338	-0.712274	4.142765
H	3.516409	2.490229	2.845521
H	3.784920	1.971262	4.513170
H	2.234922	1.662194	3.735544
H	3.911972	-1.690000	3.797089
H	2.501864	-0.773448	4.370158
H	4.092911	-0.469572	5.070840
H	6.781279	-1.113378	1.029113
H	7.670939	0.356488	1.447418
H	7.055270	0.143385	-0.191484
H	4.717478	2.675908	1.183375
H	5.896249	2.303719	-0.076738
H	6.441899	2.604389	1.571800
C	4.769676	0.002860	-1.649033
C	3.217089	-1.621410	-0.781563
H	2.744938	-2.134444	0.043766
C	2.991625	-2.082186	-2.062750
C	3.627164	-1.500923	-3.157749
H	2.309314	-2.913119	-2.211278
H	3.430594	-1.857391	-4.162115
C	4.537468	-0.467308	-2.925107
H	5.453873	0.829825	-1.515301
H	5.061766	-0.010553	-3.758327

6^{H}

N	3.238201	11.602929	4.887306

C	2.970352	10.234795	5.010675
H	3.167983	9.677245	4.104705
C	3.049493	12.448940	6.045983
H	2.002129	12.377951	6.392602
C	3.897211	12.091089	7.240701
C	5.274810	11.871693	7.172624
H	5.769729	11.896309	6.209795
C	2.505197	12.231739	3.757511
C	5.366094	11.550755	9.561212
C	6.003180	11.613052	8.328256
H	7.074511	11.452085	8.266589
C	3.269369	11.992105	8.479794
H	2.192225	12.115631	8.533480
C	3.990387	11.725255	9.634192
H	3.484581	11.652650	10.589836
C	3.206036	13.887387	5.477892
C	1.053938	11.745413	3.721839
H	0.997822	10.699842	3.417281
H	0.473067	12.350495	3.022134
H	0.590968	11.822745	4.708666
C	2.463453	14.898484	6.342106
H	1.400246	14.654816	6.413542
H	2.542529	15.902504	5.918138
H	2.876377	14.928001	7.355181
C	2.555563	13.737426	4.090281
H	3.111615	14.300675	3.338085
H	1.539022	14.135572	4.111148
C	4.664554	14.310374	5.346395
H	5.144549	14.383352	6.324094
H	4.719300	15.290823	4.865802
H	5.230679	13.602752	4.736736
C	3.185029	11.883779	2.447926
H	4.215591	12.245130	2.444473
H	2.647100	12.334295	1.610191
H	3.191557	10.802229	2.291731
N	2.185814	8.249055	6.200753
C	2.452853	9.617214	6.077133
H	2.255464	10.174901	6.983091
C	2.373407	7.402453	5.042444
H	3.420725	7.472146	4.695339
C	1.525620	7.760207	3.847789
C	0.148400	7.981896	3.915904
H	-0.346213	7.960315	4.878916
C	2.920037	7.620751	7.329886
C	0.057071	8.299361	1.526723
C	-0.579773	8.239610	2.759923
H	-1.650886	8.402114	2.821547
C	2.153220	7.856847	2.608384
H	3.230137	7.731324	2.554543
C	1.432544	8.123289	1.453704
H	1.938408	8.194331	0.497996
C	2.215853	5.964588	5.611720
C	4.371593	8.106296	7.363290
H	4.428710	9.152076	7.666960
H	4.953067	7.501349	8.062608
H	4.833346	8.028227	6.375960
C	2.955625	4.951608	4.747316
H	4.019062	5.193730	4.673960

H	2.875872	3.948120	5.172401
H	2.540901	4.921614	3.734991
C	2.868542	6.114877	6.998219
H	2.313182	5.552475	7.751573
H	3.884871	5.716256	6.976233
C	0.756939	5.543756	5.745628
H	0.275403	5.470590	4.768713
H	0.701533	4.563708	6.226977
H	0.192783	6.252507	6.355826
C	2.241938	7.969832	8.640131
H	1.211092	7.609306	8.644939
H	2.780518	7.519464	9.477534
H	2.236314	9.051502	8.795592
H	-0.515088	8.495696	0.627292
H	5.938253	11.353782	10.460516

7[H]

N	2.700309	11.675819	4.986969
C	2.672831	10.345896	5.002978
H	2.660525	9.882281	4.026561
C	2.783219	12.557564	6.149994
H	1.783732	12.602729	6.602130
C	3.757565	12.139769	7.214301
C	4.996278	11.573120	6.923837
H	5.263511	11.341475	5.899226
C	2.361721	12.414514	3.737452
C	5.568112	11.575977	9.261979
C	5.894097	11.292479	7.941569
H	6.852451	10.846597	7.702225
C	3.428947	12.398436	8.541160
H	2.456534	12.816612	8.778087
C	4.330564	12.126843	9.560640
H	4.058538	12.343896	10.587403
C	3.074807	13.954798	5.535381
C	0.995917	11.971446	3.224720
H	1.003485	10.947626	2.844318
H	0.695659	12.630995	2.408483
H	0.247706	12.044467	4.016444
C	2.546308	15.063179	6.430111
H	1.473121	14.957893	6.608387
H	2.725769	16.032535	5.958745
H	3.059092	15.064240	7.395300
C	2.299697	13.867749	4.220559
H	2.696349	14.550801	3.468722
H	1.256296	14.141715	4.396930
C	4.559678	14.197481	5.289915
H	5.092189	14.340580	6.231891
H	4.681775	15.102520	4.690396
H	5.040913	13.377599	4.757493
C	3.427586	12.183399	2.674996
H	4.410647	12.510269	3.011267
H	3.161820	12.747491	1.779590
H	3.488572	11.127965	2.398101
N	2.636135	8.216324	6.145031
C	2.635385	9.546907	6.136989
H	2.615104	10.009428	7.114039
C	2.669026	7.355575	4.963612
H	3.685241	7.405282	4.551516

C	1.704575	7.719312	3.870454
C	0.421121	8.191716	4.131233
H	0.112028	8.393685	5.150391
C	2.910848	7.472327	7.406689
C	-0.088572	8.164419	1.779256
C	-0.468621	8.413755	3.092435
H	-1.463575	8.785415	3.308828
C	2.089013	7.503238	2.550541
H	3.097391	7.165355	2.336789
C	1.195314	7.713915	1.509660
H	1.508942	7.528120	0.488711
C	2.463445	5.920675	5.527802
C	4.193757	7.982663	8.051061
H	4.086139	8.989012	8.460640
H	4.462939	7.313704	8.870462
H	5.010951	7.985931	7.327090
C	3.179834	4.899697	4.659465
H	4.246576	5.123492	4.580584
H	3.068013	3.902877	5.092757
H	2.755326	4.874764	3.652521
C	3.106319	6.035450	6.910419
H	2.682586	5.316401	7.612617
H	4.176666	5.827994	6.831759
C	0.994265	5.523133	5.633504
H	0.556834	5.372692	4.644926
H	0.916276	4.583822	6.185811
H	0.391331	6.266866	6.154074
C	1.744245	7.618381	8.373103
H	0.816368	7.234151	7.950344
H	1.968197	7.059401	9.283145
H	1.591445	8.664152	8.650483
H	-0.787252	8.335739	0.968751
H	6.271653	11.352969	10.055604

8^H

N	2.108001	11.789678	5.099917
C	1.677937	10.600752	5.214342
H	1.055581	10.190030	4.424903
C	2.914403	12.479980	6.130802
H	2.188610	12.758475	6.904077
C	3.962567	11.593364	6.737150
C	4.728622	10.714436	5.968071
H	4.557444	10.621573	4.900887
C	1.739247	12.687398	3.947126
C	6.006639	10.104405	7.911131
C	5.733119	9.962638	6.557446
H	6.317717	9.280341	5.951610
C	4.234231	11.718255	8.096579
H	3.633056	12.387191	8.702397
C	5.260896	10.990730	8.677635
H	5.472628	11.109767	9.733488
C	3.386112	13.786574	5.445800
C	0.232102	12.661881	3.770493
H	-0.120519	11.705876	3.377315
H	-0.035604	13.433433	3.047854
H	-0.275221	12.878539	4.711580
C	3.469389	14.911392	6.465071
H	2.509155	15.076648	6.958623

H	3.768259	15.833072	5.960478
H	4.220803	14.691567	7.227310
C	2.245868	14.037247	4.450216
H	2.558688	14.664632	3.616411
H	1.428928	14.547507	4.965879
C	4.752003	13.649081	4.785355
H	5.529184	13.559029	5.547162
H	4.956899	14.547191	4.200309
H	4.840356	12.790969	4.120720
C	2.434981	12.211843	2.679527
H	3.517464	12.296713	2.739315
H	2.092585	12.843377	1.859790
H	2.165922	11.179444	2.442803
N	2.152762	8.582231	6.485254
C	1.829341	9.813917	6.441810
H	1.498319	10.289480	7.361583
C	2.776111	7.839577	5.365738
H	3.741850	8.339786	5.236053
C	2.011485	7.947858	4.080894
C	0.621365	7.842809	4.041642
H	0.052601	7.722400	4.956468
C	2.072144	7.763064	7.747824
C	0.661813	8.083605	1.650513
C	-0.050524	7.920396	2.833767
H	-1.131136	7.846229	2.815057
C	2.718495	8.125387	2.895238
H	3.798729	8.219404	2.919664
C	2.045519	8.178750	1.682541
H	2.604889	8.308347	0.764103
C	3.070994	6.417285	5.911159
C	2.492891	8.561739	8.962887
H	1.811435	9.386712	9.178068
H	2.456828	7.888594	9.820397
H	3.509274	8.941676	8.859747
C	4.462338	5.998922	5.445192
H	5.220908	6.711205	5.778945
H	4.713501	5.017652	5.852641
H	4.508056	5.932119	4.354981
C	3.049904	6.635016	7.430109
H	2.777356	5.726760	7.966441
H	4.045811	6.932360	7.768595
C	2.068224	5.364957	5.452891
H	2.141325	5.210810	4.374557
H	2.304574	4.420920	5.947449
H	1.032810	5.611427	5.684077
C	0.617809	7.327780	7.906085
H	0.219589	6.843632	7.017253
H	0.562137	6.619625	8.733321
H	-0.007149	8.189914	8.145177
H	0.134291	8.138783	0.705788
H	6.796871	9.521337	8.367875

3^{Me}

C	-1.826636	0.304642	-1.311480
C	-0.288005	0.271746	-1.337775
H	-2.223938	-0.708006	-1.370002
H	-2.202209	0.860469	-2.169741
C	-2.259299	0.971822	0.011885

C	0.309008	-1.124117	-1.320034
N	0.047164	0.952458	-0.051258
C	0.301188	1.065565	-2.491476
C	-0.960055	1.350863	0.635069
C	-2.923751	-0.025661	0.962955
C	1.422404	1.079925	0.376558
H	-2.321098	-0.931145	1.063944
H	-3.899028	-0.293505	0.552459
H	-3.076606	0.406843	1.952214
C	-3.121407	2.211735	-0.187857
C	-0.785597	2.048434	1.921200
H	-2.626966	2.926500	-0.848320
H	-3.328121	2.695886	0.767778
H	-4.072800	1.923045	-0.637110
C	-0.528161	3.419058	1.936061
C	-0.206978	4.032302	3.137346
C	-0.122855	3.308401	4.324979
C	-0.471826	1.958427	4.307841
C	-0.784249	1.327101	3.112447
H	-0.545200	3.987121	1.014037
H	0.034434	5.089651	3.139892
C	0.431724	3.941725	5.559779
H	-0.440378	1.381042	5.224247
H	-0.992817	0.263816	3.108619
H	-0.023963	-1.704016	-0.457641
H	1.395392	-1.077162	-1.333724
H	-0.007251	-1.640750	-2.226486
H	-0.012529	0.586962	-3.420326
H	1.391915	1.077804	-2.481110
H	-0.070025	2.091704	-2.487953
C	1.788321	-0.039154	1.355604
H	1.555342	2.056083	0.834810
H	2.052982	1.035515	-0.506111
H	1.132875	-0.021300	2.221615
N	3.144720	0.196946	1.809852
H	1.711651	-1.021366	0.900287
C	4.189759	0.060062	1.065527
C	5.361230	0.783346	1.663037
C	4.077336	-0.650515	-0.218151
C	4.915906	0.891918	3.131750
C	5.419623	2.154747	0.961351
C	6.692802	0.076085	1.499291
C	3.388061	0.847007	3.134543
H	5.288965	1.799495	3.602787
H	5.299011	0.027352	3.676150
C	2.721637	2.212026	3.177888
C	2.811871	-0.029566	4.236038
H	3.062214	2.879701	2.387970
H	2.952498	2.670999	4.138608
H	1.642530	2.108462	3.124013
H	3.186014	-1.052007	4.164333
H	1.720748	-0.047142	4.229538
H	3.123872	0.385032	5.195455
H	6.656389	-0.941372	1.892429
H	7.458022	0.633180	2.042445
H	6.983085	0.038700	0.447289
H	4.468427	2.683547	0.985593
H	5.725082	2.050329	-0.080238

H	6.168577	2.760607	1.473640
C	4.070362	0.039590	-1.427612
C	3.795602	-2.020990	-0.207611
H	3.815801	-2.572382	0.724829
C	3.441761	-2.653210	-1.388097
C	3.315176	-1.947174	-2.584490
H	3.195316	-3.709448	-1.366686
C	2.707900	-2.597317	-3.785172
C	3.687305	-0.603029	-2.597309
H	4.292049	1.099259	-1.455654
H	3.634186	-0.039284	-3.521204
H	0.084363	4.972323	5.655665
H	1.524443	3.973838	5.507688
H	0.155306	3.381467	6.453206
H	2.959625	-2.062306	-4.700760
H	1.616952	-2.613653	-3.696996
H	3.035229	-3.635654	-3.868819

4^{Me}

C	-1.550249	1.227870	-1.585161
C	-0.026969	1.143590	-1.468770
H	-1.938368	0.453367	-2.245219
H	-1.828339	2.196957	-2.003177
C	-2.116780	1.105315	-0.163535
C	0.512517	-0.251261	-1.736023
N	0.176266	1.453140	-0.013758
C	0.665117	2.183441	-2.327717
C	-0.908639	1.410701	0.676673
C	-2.638532	-0.302883	0.142964
C	1.522584	1.679306	0.507759
H	-1.870725	-1.065083	0.005305
H	-3.459439	-0.515287	-0.544304
H	-3.026204	-0.374824	1.159432
C	-3.211242	2.136059	0.098044
C	-0.964024	1.692402	2.113717
H	-2.841939	3.150238	-0.069419
H	-3.589070	2.062775	1.119573
H	-4.037604	1.954022	-0.591798
C	-0.734436	2.990313	2.562734
C	-0.820585	3.278912	3.914820
C	-1.126039	2.287686	4.844898
C	-1.345911	0.992713	4.379082
C	-1.279375	0.692462	3.031263
H	-0.508289	3.778766	1.853972
H	-0.651626	4.295981	4.250502
C	-1.205197	2.584262	6.308462
H	-1.581187	0.204614	5.085994
H	-1.437980	-0.325490	2.700255
H	0.015729	-0.996606	-1.114780
H	1.585828	-0.312954	-1.550808
H	0.333339	-0.501769	-2.782615
H	0.351010	2.028391	-3.361411
H	1.752437	2.096220	-2.293572
H	0.373289	3.192264	-2.029421
C	1.913955	0.716871	1.638807
H	1.600811	2.720724	0.814684
H	2.209525	1.521443	-0.321348
H	1.459599	1.020269	2.579310

N	3.336491	0.642206	1.832305
H	1.505926	-0.263371	1.387146
C	4.191544	0.173797	0.857106
C	5.552673	0.813448	1.006154
C	3.820625	-0.878886	-0.032960
C	5.474222	1.325032	2.456101
C	5.772626	1.964732	0.015449
C	6.699093	-0.184512	0.876024
C	3.997282	1.549685	2.789946
H	6.062442	2.231328	2.607390
H	5.874882	0.555431	3.120584
C	3.580270	3.006681	2.600485
C	3.692660	1.140748	4.225093
H	3.756623	3.358289	1.583785
H	4.159878	3.635526	3.278505
H	2.524994	3.155231	2.845897
H	3.921107	0.085053	4.383122
H	2.645671	1.325117	4.483384
H	4.306706	1.733355	4.907610
H	6.550201	-1.032109	1.548372
H	7.642899	0.301368	1.138745
H	6.789785	-0.570560	-0.141640
H	4.943201	2.673516	0.025964
H	5.882882	1.590157	-1.004393
H	6.687191	2.508720	0.269470
C	4.316806	-0.950929	-1.353939
C	2.922418	-1.903242	0.354277
H	2.583106	-1.952154	1.382348
C	2.500599	-2.869923	-0.535949
C	2.950356	-2.894750	-1.857475
H	1.802757	-3.629882	-0.196852
C	2.473214	-3.933177	-2.824312
C	3.882263	-1.924223	-2.232981
H	5.010210	-0.199001	-1.708965
H	4.261762	-1.922091	-3.250681
H	-1.419525	3.638108	6.492397
H	-0.256535	2.339305	6.796315
H	-1.976659	1.980422	6.788120
H	2.714166	-3.650456	-3.850776
H	1.391862	-4.077453	-2.753650
H	2.940466	-4.904028	-2.627592

5^{Me}

C	-2.503736	-0.946990	0.839532
C	-1.027692	-1.069937	0.472872
H	-2.668391	-1.458360	1.791832
H	-3.144050	-1.421406	0.094157
C	-2.811969	0.540004	1.016391
C	-0.514662	-2.400408	1.018966
N	-0.449236	0.123022	1.148934
C	-0.789551	-1.027994	-1.035742
C	-1.440246	1.072598	1.387437
C	-3.837413	0.709193	2.137073
C	0.867670	0.586720	0.718116
H	-3.438089	0.325052	3.078788
H	-4.742299	0.145732	1.891339
H	-4.122895	1.751036	2.289949
C	-3.387685	1.116467	-0.286080

C	-1.214347	2.321818	2.034987
H	-2.691998	1.016079	-1.118466
H	-3.649278	2.171130	-0.193880
H	-4.301932	0.573934	-0.544848
C	-2.115465	3.400063	1.859473
C	-1.881012	4.644765	2.403517
C	-0.752323	4.913241	3.179173
C	0.105057	3.840319	3.434738
C	-0.119355	2.586681	2.897097
H	-2.992985	3.273301	1.243585
H	-2.584932	5.446739	2.201286
C	-0.494988	6.292016	3.703629
H	0.967231	3.985008	4.079890
H	0.541768	1.779263	3.180121
H	-0.491545	-2.387665	2.110427
H	0.471093	-2.667465	0.639161
H	-1.206495	-3.186425	0.703868
H	-1.399923	-1.791526	-1.521363
H	0.253152	-1.238953	-1.282023
H	-1.048482	-0.059889	-1.464925
C	1.972127	-0.471045	0.826227
H	1.147673	1.434613	1.335204
H	0.808162	0.969375	-0.309791
H	1.644155	-1.232704	1.532842
N	3.267483	0.019787	1.256241
H	2.118826	-0.971055	-0.131427
C	4.424490	-0.233866	0.550243
C	5.469578	-0.849153	1.460758
C	4.573152	0.126656	-0.810901
C	4.914047	-0.532041	2.865724
C	6.885442	-0.295248	1.325806
C	5.485987	-2.371448	1.263145
C	3.577361	0.208992	2.685845
H	5.614219	0.079847	3.436418
H	4.761177	-1.458033	3.423959
C	3.771213	1.705609	2.925393
C	2.521645	-0.362143	3.616876
H	4.537769	2.088411	2.247245
H	4.091721	1.898641	3.952932
H	2.848631	2.258810	2.733801
H	2.442948	-1.446627	3.512925
H	1.532222	0.067156	3.450575
H	2.813289	-0.149417	4.647060
H	4.483092	-2.784730	1.396773
H	6.144410	-2.840017	2.001046
H	5.843176	-2.649829	0.269156
H	6.891659	0.792760	1.429200
H	7.351889	-0.554700	0.375914
H	7.508883	-0.712956	2.120549
C	5.625312	-0.370284	-1.622703
C	3.704448	1.064417	-1.427196
H	2.888132	1.480567	-0.851350
C	3.922270	1.515774	-2.714966
C	5.001410	1.065157	-3.481426
H	3.247618	2.258096	-3.131717
C	5.288861	1.616772	-4.845415
C	5.826822	0.091837	-2.906302
H	6.285207	-1.135654	-1.238497

H	6.651658	-0.311674	-3.486311
H	-1.104581	7.026419	3.174620
H	0.554598	6.573125	3.580849
H	-0.737607	6.373398	4.769565
H	6.059316	2.394925	-4.805209
H	4.398638	2.070076	-5.286700
H	5.658922	0.842412	-5.521515

6^{Me}

N	3.226673	11.593101	4.861486
C	2.997453	10.224345	5.018591
H	3.254437	9.643908	4.142753
C	3.005560	12.461252	5.998674
H	1.957543	12.374996	6.339470
C	3.848265	12.081305	7.191313
C	5.201053	11.753712	7.098701
H	5.702044	11.836170	6.141820
C	2.485951	12.168863	3.712368
C	5.248992	11.037818	9.418878
C	5.884196	11.252027	8.195753
H	6.930360	10.979205	8.093207
C	3.234531	11.933957	8.430448
H	2.176894	12.161806	8.521878
C	3.914475	11.417962	9.523812
H	3.391233	11.275837	10.463933
C	3.132215	13.884025	5.392963
C	1.047345	11.652246	3.710492
H	1.029059	10.576997	3.556965
H	0.474027	12.136422	2.916000
H	0.553027	11.850081	4.664872
C	5.963889	10.343387	10.534408
H	7.005823	10.669022	10.587630
H	5.975006	9.259869	10.377180
H	5.486999	10.533070	11.496645
C	2.322828	14.888337	6.201085
H	1.268948	14.600913	6.242070
H	2.375227	15.881672	5.747679
H	2.693122	14.962353	7.228001
C	2.523902	13.683434	3.990436
H	3.103987	14.219105	3.235918
H	1.506826	14.079896	3.962356
C	4.582025	14.339951	5.287418
H	5.043114	14.418465	6.273953
H	4.626864	15.320856	4.806201
H	5.170293	13.641865	4.687671
C	3.161361	11.775843	2.413285
H	4.191281	12.139079	2.394669
H	2.620020	12.199039	1.563333
H	3.166642	10.691199	2.290030
N	2.204376	8.257987	6.227989
C	2.435339	9.626603	6.072034
H	2.178576	10.206808	6.948108
C	2.417362	7.390426	5.089153
H	3.464261	7.472199	4.743554
C	1.571085	7.775204	3.900475
C	0.218008	8.100950	3.995959
H	-0.281753	8.016438	4.953358
C	2.947377	7.678732	7.373497

C	0.164575	8.820080	1.676771
C	-0.468160	8.602600	2.900681
H	-1.514642	8.873209	3.005695
C	2.182322	7.926670	2.660531
H	3.240237	7.701233	2.566592
C	1.499622	8.443190	1.569424
H	2.021391	8.588541	0.628974
C	2.287256	5.967387	5.694257
C	4.388009	8.189662	7.369837
H	4.412256	9.263797	7.530877
H	4.965935	7.696982	8.155668
H	4.874452	7.998071	6.410174
C	-0.553675	9.514578	0.563328
H	-1.595502	9.188369	0.511977
H	-0.565019	10.598032	0.721278
H	-0.079224	9.326041	-0.400317
C	3.087768	4.959350	4.881397
H	4.143266	5.240237	4.836475
H	3.031561	3.965773	5.333772
H	2.712544	4.888615	3.856037
C	2.902907	6.164447	7.094281
H	2.325019	5.629802	7.851185
H	3.918928	5.764932	7.116624
C	0.835787	5.518413	5.805231
H	0.371193	5.440933	4.820258
H	0.787754	4.538240	6.287636
H	0.252690	6.219870	6.406088
C	2.277695	8.073556	8.674912
H	1.247558	7.711230	8.697837
H	2.821907	7.651058	9.523383
H	2.273982	9.158372	8.797135

7^{Me}

N	2.316327	11.827267	5.120146
C	2.175665	10.502837	5.104802
H	1.988434	10.064037	4.135092
C	2.778427	12.625609	6.255244
H	1.927460	12.784934	6.931604
C	3.899868	12.022170	7.051179
C	4.940926	11.302805	6.473231
H	4.935804	11.094733	5.409678
C	1.720184	12.670739	4.047512
C	6.037557	11.066277	8.619298
C	5.990232	10.832634	7.247394
H	6.785287	10.263922	6.776185
C	3.937904	12.250994	8.423850
H	3.126804	12.794542	8.897514
C	4.993514	11.788500	9.192945
H	4.998599	11.984099	10.260621
C	3.109164	13.995370	5.604128
C	0.220540	12.404708	3.978175
H	0.008620	11.405169	3.589869
H	-0.241402	13.131381	3.307520
H	-0.236232	12.508336	4.964243
C	3.034580	15.128026	6.612355
H	2.043662	15.197743	7.068640
H	3.256342	16.074848	6.112858
H	3.771726	14.990538	7.407557

C	1.996697	14.090492	4.558949
H	2.257205	14.764740	3.742486
H	1.092690	14.479001	5.035535
C	4.492583	14.019253	4.965955
H	5.269109	14.011697	5.733598
H	4.602048	14.936320	4.382734
H	4.673087	13.174052	4.301369
C	2.352470	12.390442	2.691254
H	3.418206	12.618008	2.682751
H	1.863097	13.016602	1.943956
H	2.208146	11.349446	2.389693
N	2.274373	8.388485	6.327728
C	2.204099	9.713392	6.246866
H	2.147757	10.215788	7.203312
C	2.643261	7.462169	5.252786
H	3.729626	7.561843	5.123643
C	2.004306	7.694671	3.916009
C	0.645702	7.969263	3.772916
H	0.026523	8.104573	4.652273
C	2.388415	7.745412	7.671585
C	0.844884	7.925891	1.359381
C	0.079178	8.084307	2.516377
H	-0.981519	8.295331	2.429368
C	2.777253	7.566366	2.768366
H	3.841272	7.372847	2.856819
C	2.203509	7.673741	1.508637
H	2.825625	7.555849	0.627413
C	2.388381	6.057603	5.859812
C	3.518733	8.382439	8.469349
H	3.280795	9.393017	8.805645
H	3.706062	7.771357	9.354512
H	4.434475	8.421588	7.877411
C	3.312602	5.027736	5.233410
H	4.359414	5.322565	5.342085
H	3.177677	4.060985	5.724155
H	3.096031	4.898814	4.169445
C	2.745434	6.295612	7.327547
H	2.229630	5.597072	7.987601
H	3.819440	6.148296	7.468050
C	0.944901	5.587361	5.709489
H	0.702398	5.382175	4.665030
H	0.811729	4.663098	6.276162
H	0.223597	6.314635	6.082930
C	1.070007	7.872960	8.421304
H	0.256021	7.381040	7.889071
H	1.172432	7.403971	9.401497
H	0.804440	8.921709	8.573385
C	0.217369	8.025020	0.003513
C	7.155022	10.529141	9.458026
H	7.929895	10.073960	8.839758
H	6.783064	9.768179	10.150091
H	7.611615	11.316472	10.063301
H	0.926362	7.747577	-0.777852
H	-0.134189	9.041258	-0.197423
H	-0.650717	7.366294	-0.073416

8^{Me}

N	1.721854	11.837257	5.241700

C	1.026642	10.773416	5.235835
H	0.305721	10.597667	4.440561
C	2.695082	12.135567	6.316007
H	2.177264	11.924049	7.258015
C	3.942867	11.307713	6.223587
C	4.499650	10.944477	5.001469
H	3.937808	11.048482	4.083106
C	1.499495	12.998777	4.307386
C	6.589361	10.383003	6.077571
C	5.801199	10.485284	4.933095
H	6.227662	10.243462	3.965309
C	4.678263	11.091817	7.383412
H	4.249248	11.335424	8.349598
C	5.988446	10.640758	7.307135
H	6.562454	10.521586	8.219356
C	2.942472	13.676007	6.235103
C	0.043000	13.044840	3.890577
H	-0.213037	12.243045	3.194730
H	-0.129224	13.987817	3.370861
H	-0.617362	13.003810	4.758512
C	8.042513	10.046195	5.963367
H	8.516200	10.676889	5.208081
H	8.191132	9.008874	5.649170
H	8.558082	10.205458	6.910838
C	2.720814	14.260061	7.625936
H	1.698884	14.084307	7.970338
H	2.911799	15.335254	7.619318
H	3.410342	13.805855	8.341742
C	1.869489	14.154609	5.241389
H	2.204826	15.010157	4.654838
H	0.973828	14.454562	5.789402
C	4.356638	14.049338	5.796705
H	5.094780	13.656166	6.496993
H	4.441335	15.137774	5.797864
H	4.619247	13.689298	4.803109
C	2.398341	12.888691	3.084451
H	3.453907	12.988085	3.324108
H	2.133837	13.702418	2.409047
H	2.230680	11.945915	2.559159
N	1.452249	8.830859	6.717874
C	0.914094	9.940061	6.439367
H	0.206681	10.358320	7.158647
C	2.499370	8.142122	5.932415
H	3.395171	8.748939	6.106088
C	2.236992	8.072139	4.461525
C	0.972010	7.841828	3.927195
H	0.090649	7.850141	4.559954
C	1.117037	8.104516	8.003793
C	1.934230	7.443758	1.741081
C	0.827800	7.542658	2.580247
H	-0.162388	7.347384	2.184025
C	3.344890	8.024378	3.615220
H	4.335991	8.199770	4.016788
C	3.194943	7.701499	2.280738
H	4.072415	7.627780	1.647176
C	2.721379	6.782782	6.650580
C	1.124999	9.056070	9.183035
H	0.330332	9.801933	9.122936

H	0.940547	8.461991	10.079448
H	2.089298	9.556340	9.289591
C	1.799871	7.044670	0.305164
H	0.773851	6.766559	0.061746
H	2.107707	7.857237	-0.358398
H	2.448612	6.193630	0.083846
C	4.206996	6.444343	6.624545
H	4.810575	7.268776	7.013518
H	4.396635	5.563877	7.241594
H	4.539132	6.217028	5.609250
C	2.256216	7.092568	8.076599
H	1.939494	6.194623	8.606128
H	3.081122	7.535648	8.639692
C	1.958269	5.619809	6.028202
H	2.311584	5.424907	5.014290
H	2.140095	4.726688	6.628897
H	0.882118	5.775559	5.979536
C	-0.275435	7.510922	7.835201
H	-0.366556	6.875631	6.957826
H	-0.499075	6.910356	8.716821
H	-1.016927	8.309743	7.765514

NMR Spectra

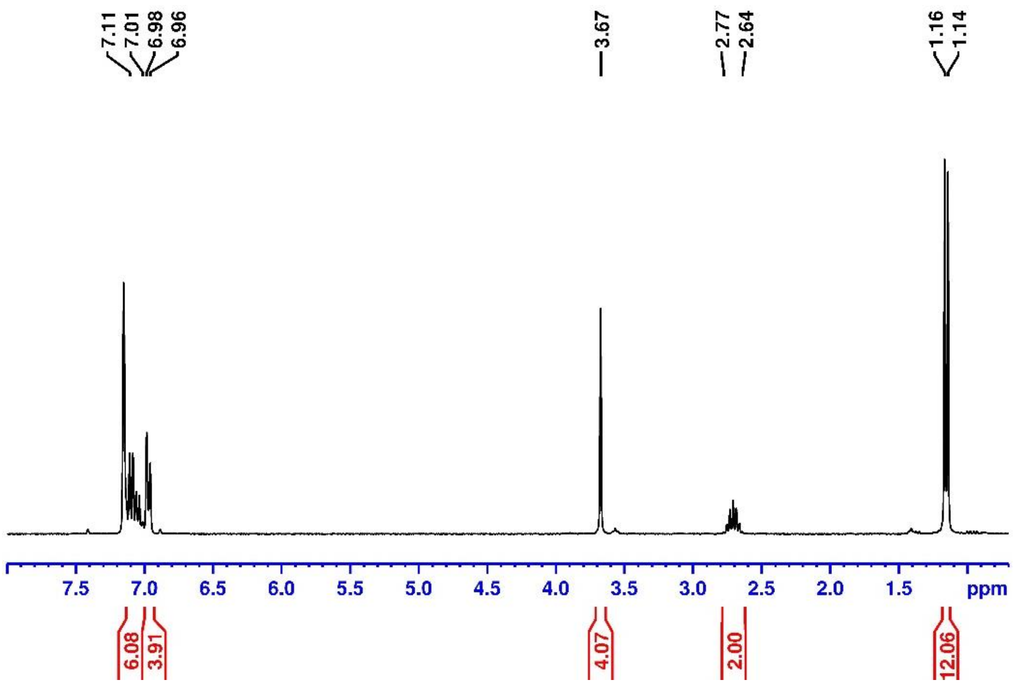

Figure S43. ^{1}H NMR spectrum of compound $\mathbf{2^{H}}$ in C_6D_6 at RT.

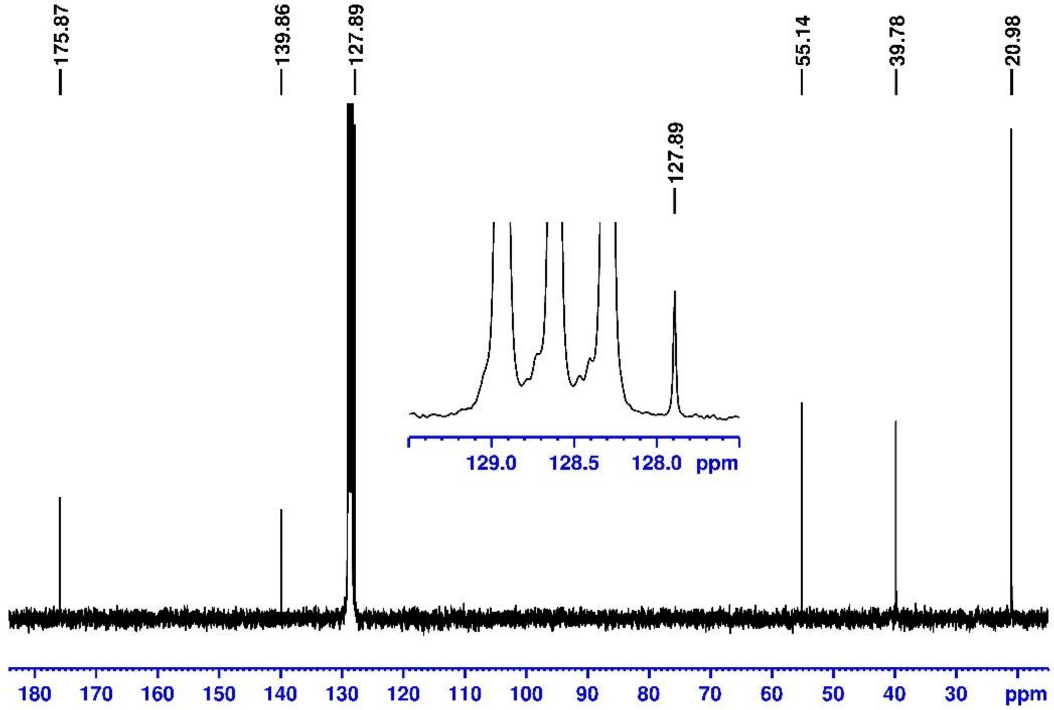

Figure S44. $^{13}C\{^{1}H\}$ NMR spectrum of compound $\mathbf{2^{H}}$ in C_6D_6 at RT.

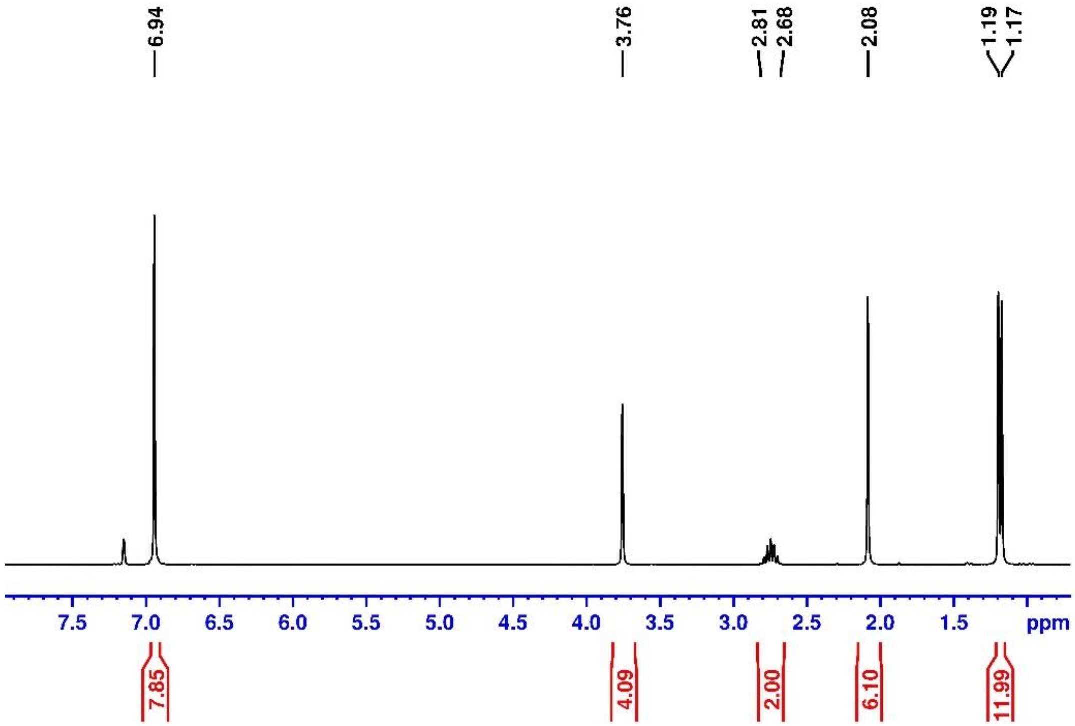

Figure S45. ^{1}H NMR spectrum of $\mathbf{2^{Me}}$ in C_6D_6 at room temperature.

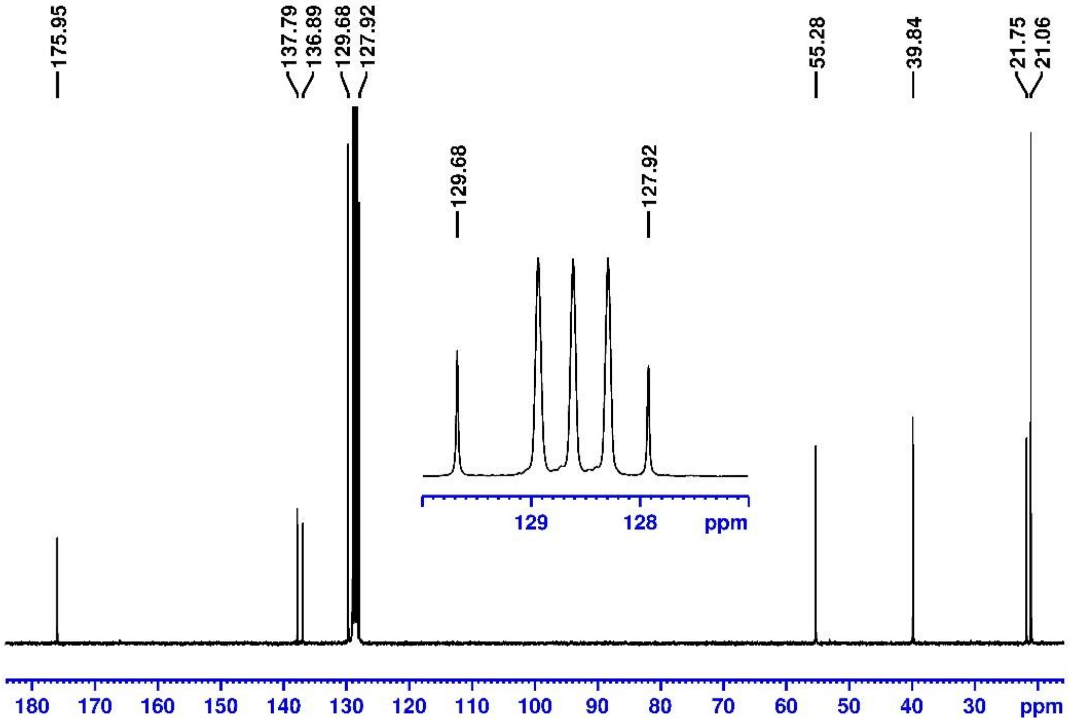

Figure S46. $^{13}C\{^{1}H\}$ NMR spectrum of $\mathbf{2^{Me}}$ in C_6D_6 at room temperature.

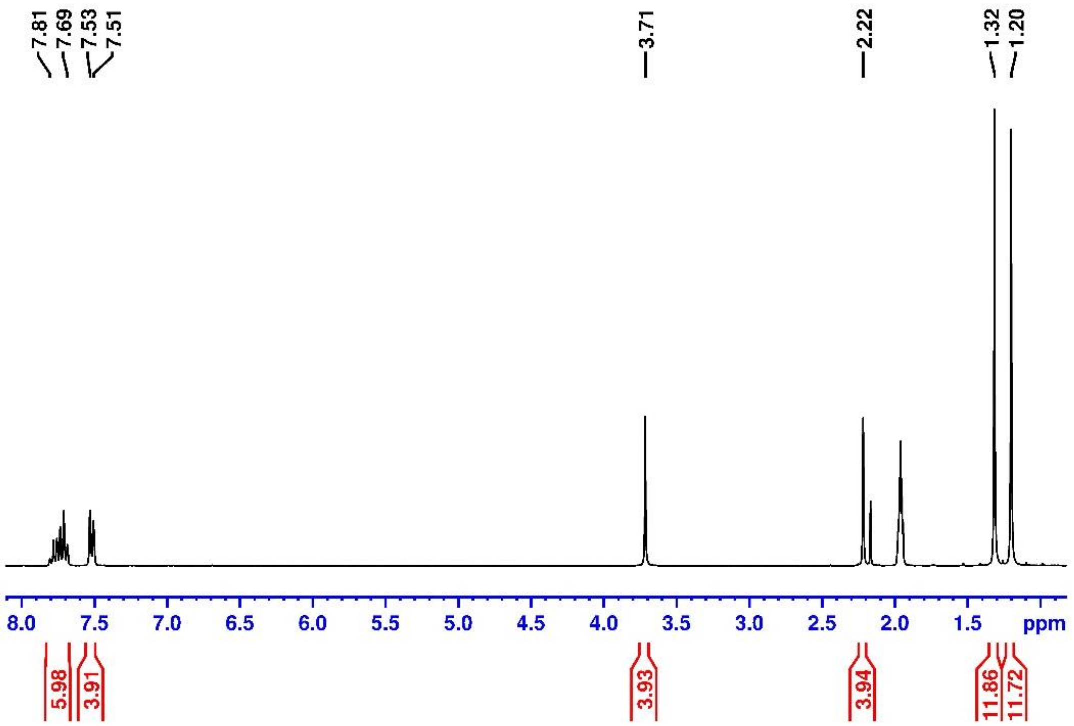

Figure S47. ^1H NMR spectrum of compound $\mathbf{3^H}$ in CD_3CN at RT.

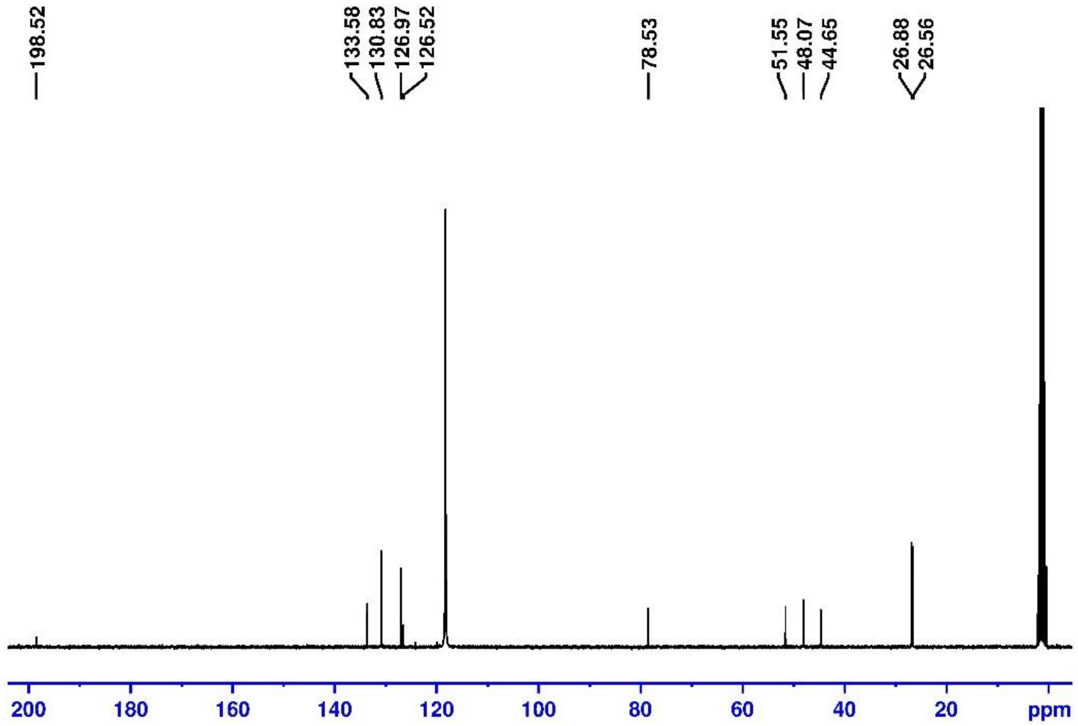

Figure S48. $^{13}C\{^1H\}$ NMR spectrum of compound $\mathbf{3^H}$ in CD_3CN at RT.

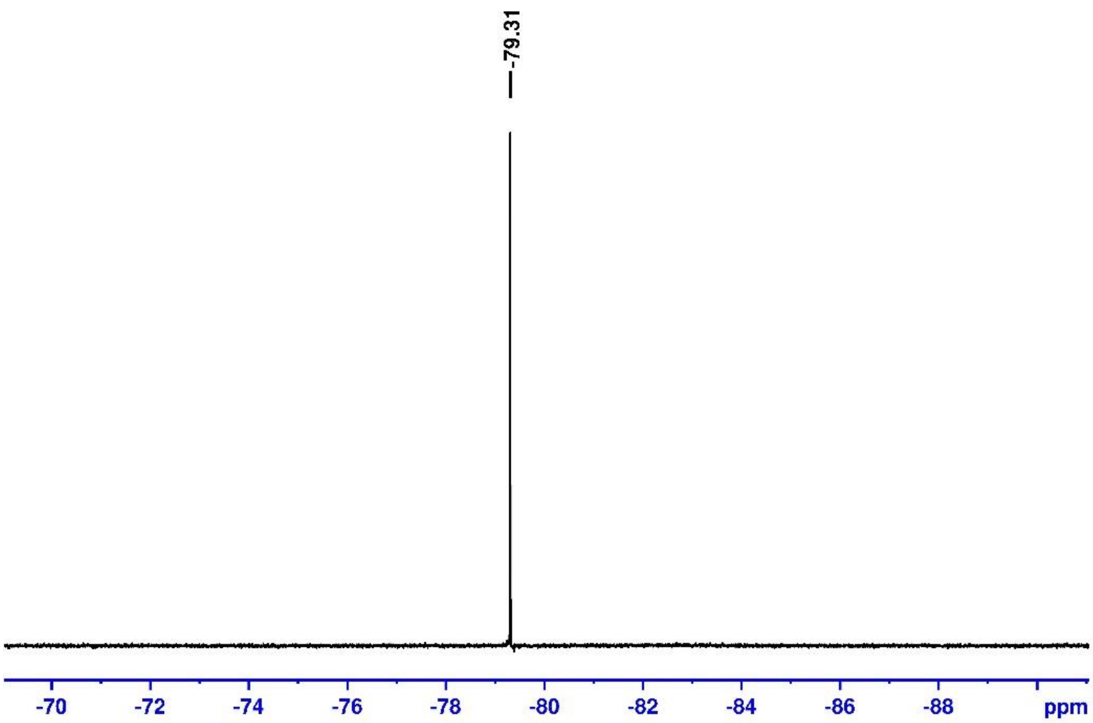

Figure S49. $^{19}F\{^{1}H\}$ NMR spectrum of compound $\mathbf{3^{H}}$ in CD_3CN at RT.

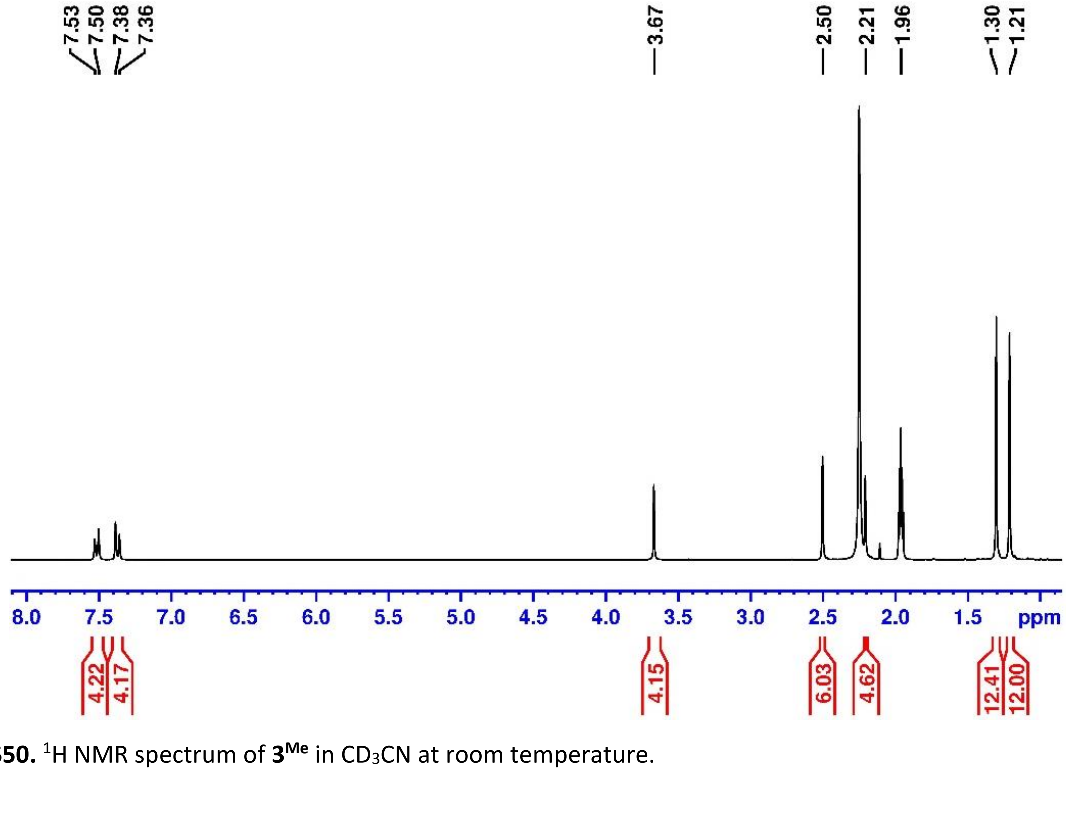

Figure S50. ^{1}H NMR spectrum of $\mathbf{3^{Me}}$ in CD_3CN at room temperature.

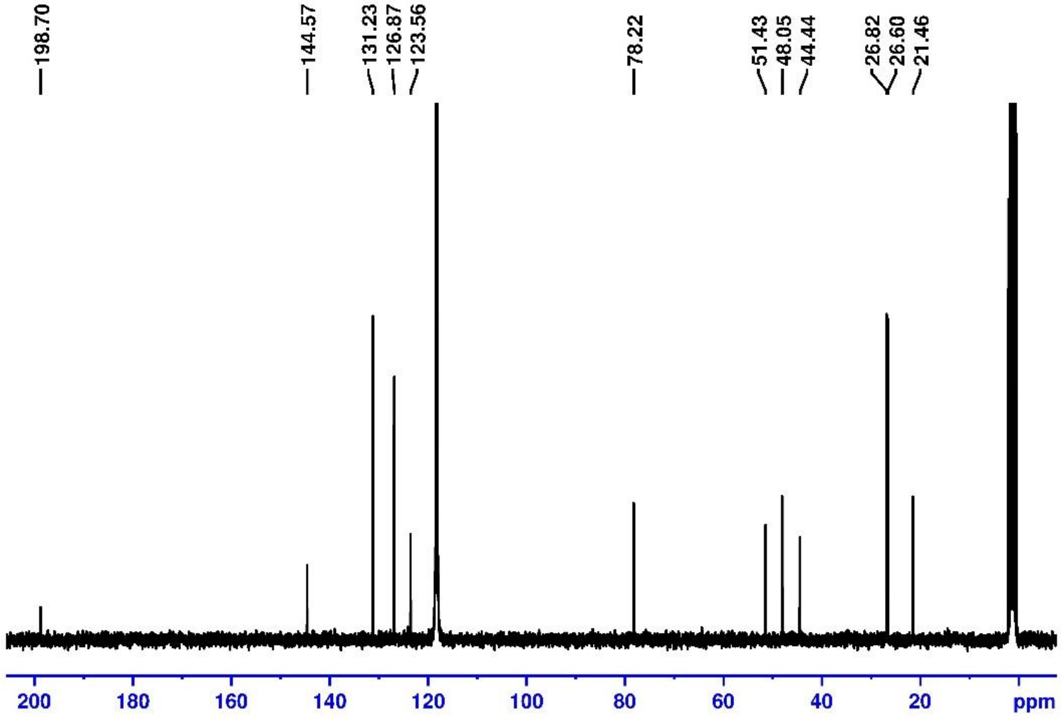

Figure S51. $^{13}C\{^1H\}$ NMR spectrum of $\mathbf{3^{Me}}$ in CD_3CN at room temperature.

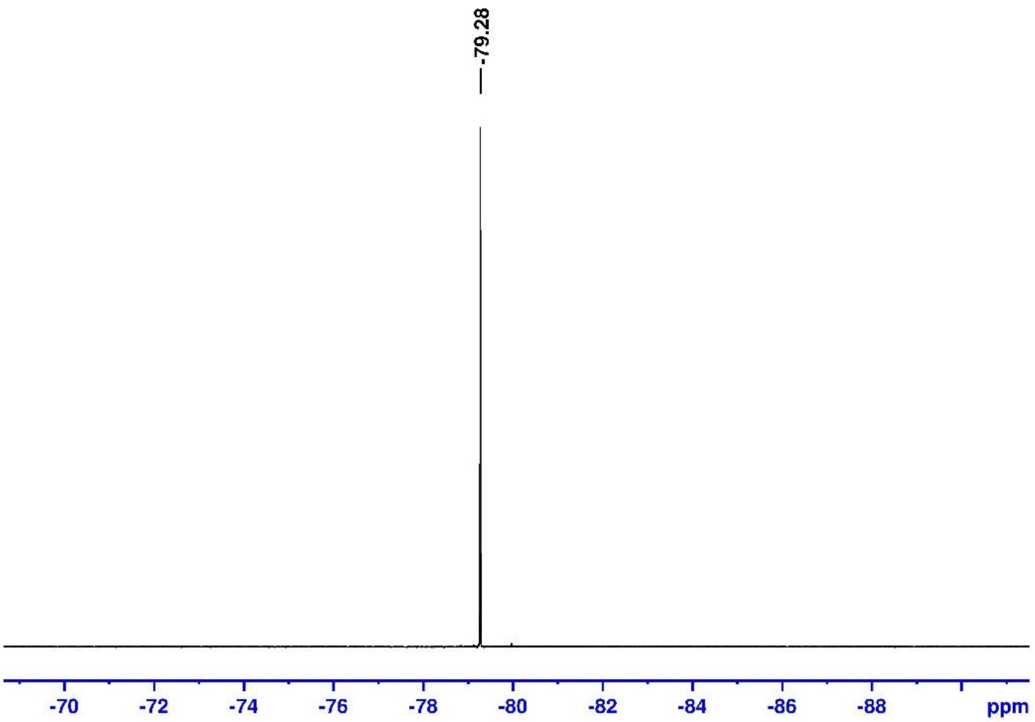

Figure S52. $^{19}F\{^1H\}$ NMR spectrum of $\mathbf{3^{Me}}$ in CD_3CN at room temperature.

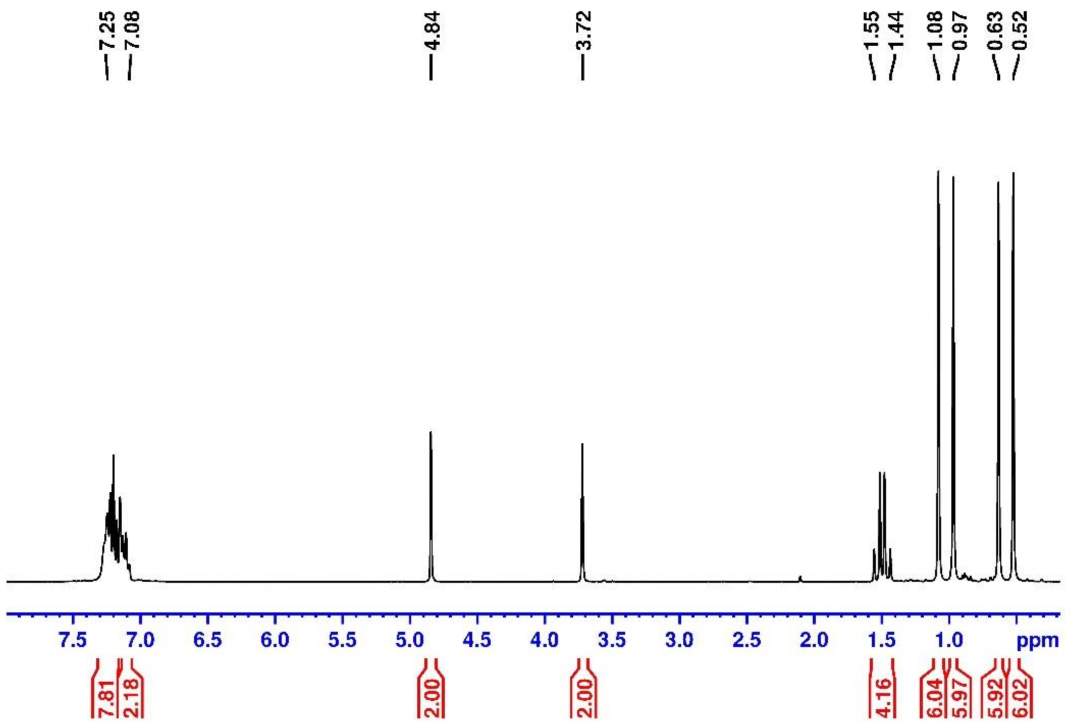

Figure S53. ^{1}H NMR spectrum of $\mathbf{6^{H}}$ in C_6D_6 at room temperature.

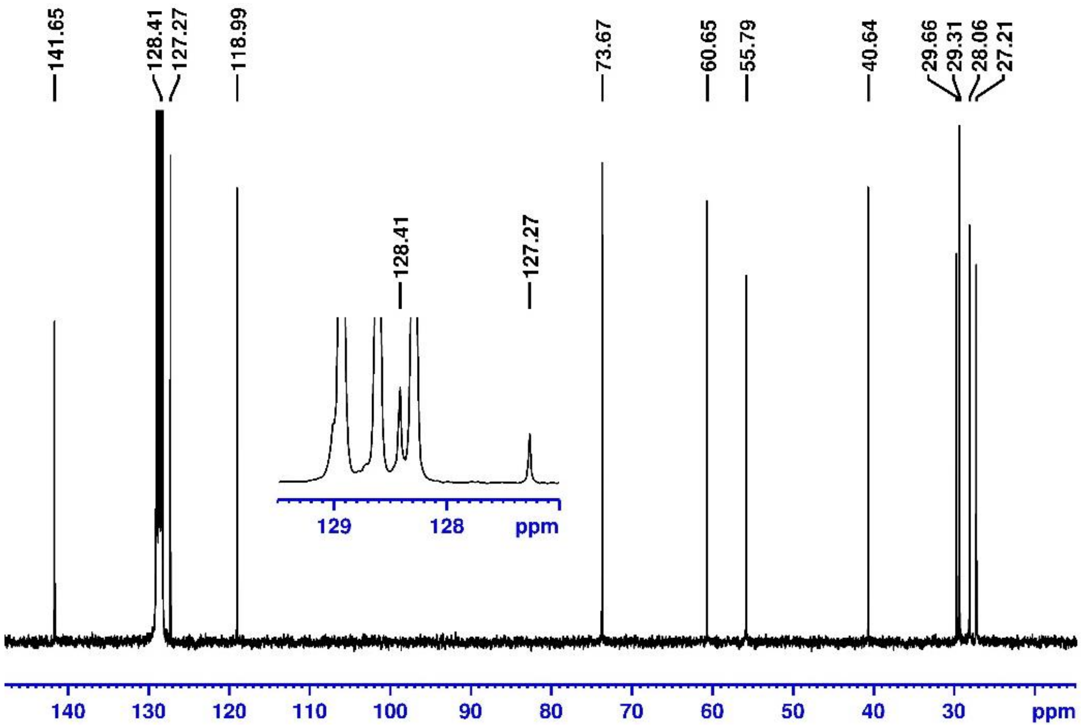

Figure S54. $^{13}C\{^{1}H\}$ NMR spectrum of $\mathbf{6^{H}}$ in C_6D_6 at room temperature.

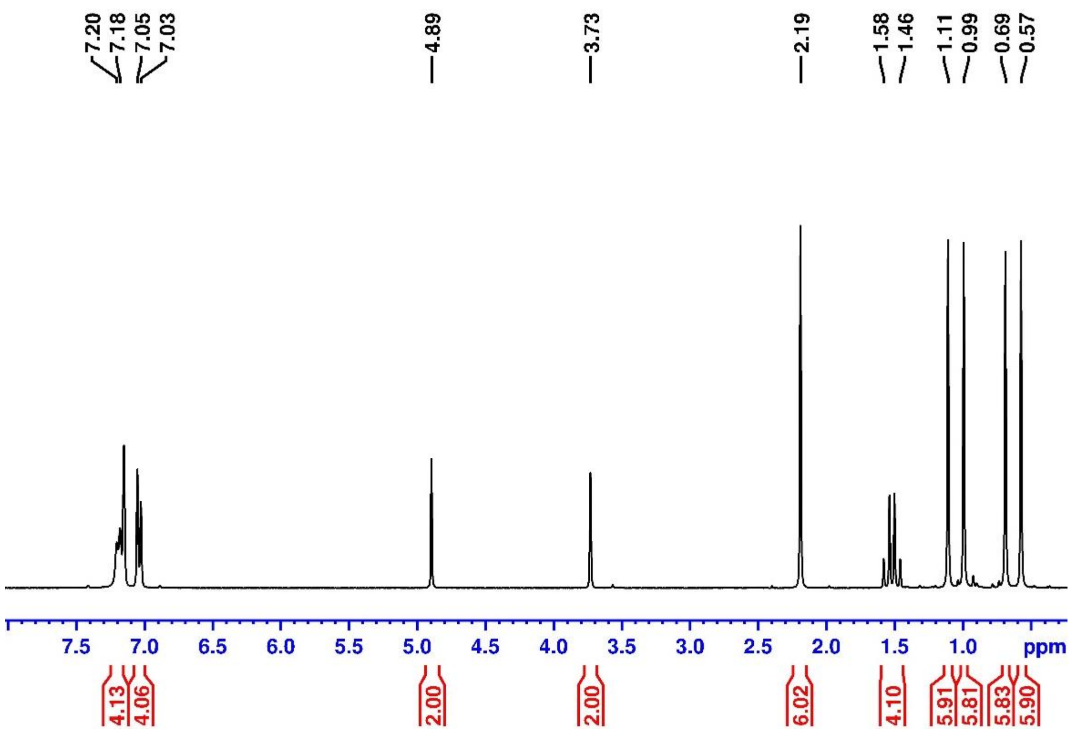

Figure S55. ^{1}H NMR spectrum of **6^{Me}** in C_6D_6 at room temperature.

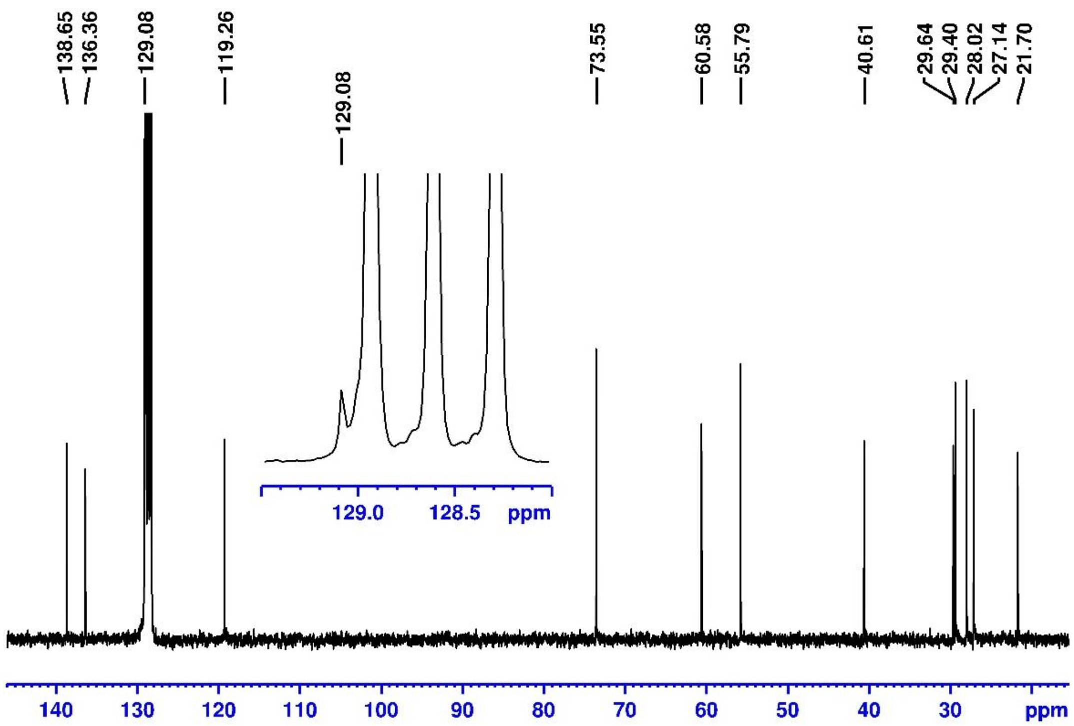

Figure S56. $^{13}C\{^{1}H\}$ NMR spectrum of **6^{Me}** in C_6D_6 at room temperature.

UV/vis Spectra

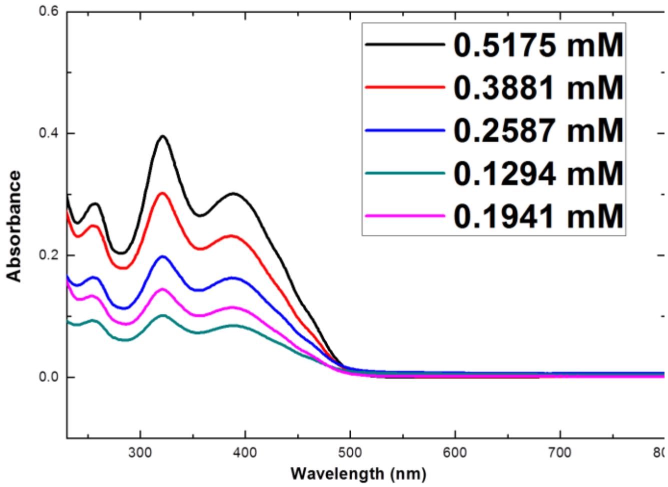

Figure S57. UV/vis spectrum of $\mathbf{7^H}$ in THF at various concentrations at room temperature.

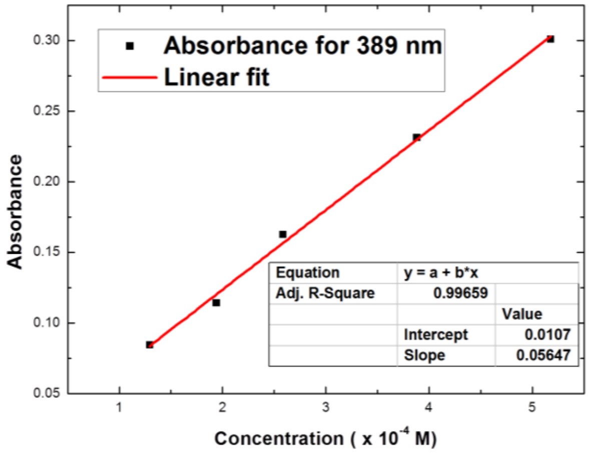

Figure S58: Linear regression of $\mathbf{7^H}$ at 389 nm.

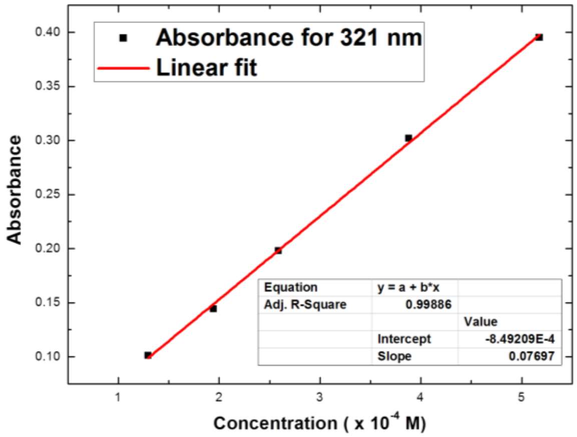

Figure S59. Linear regression of $\mathbf{7^H}$ at 321 nm.

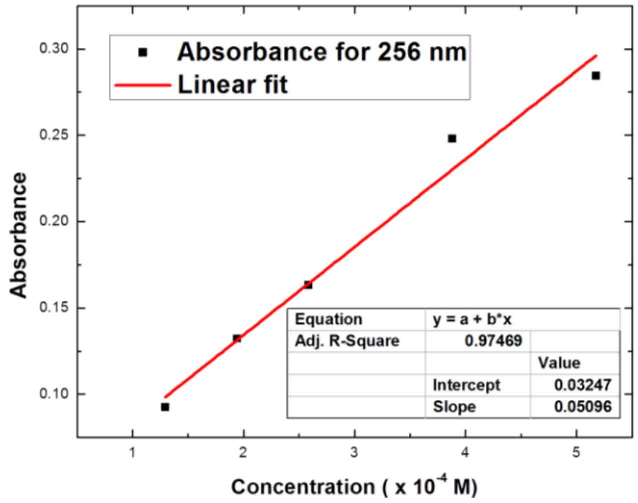

Figure S60. Linear regression of $\mathbf{7^{H}}$ at 256 nm.

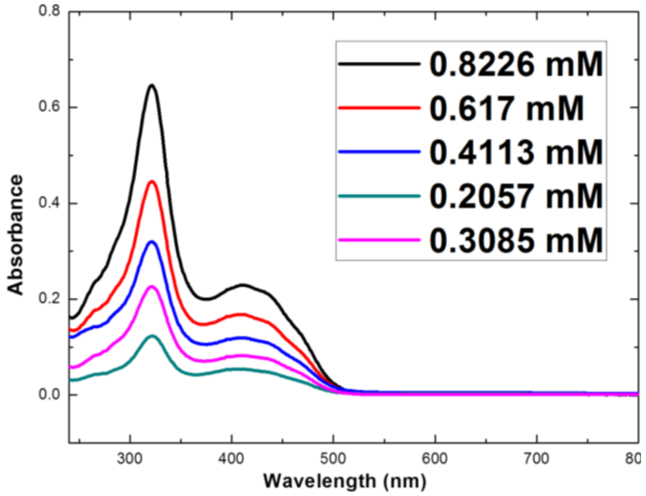

Figure S61. UV/Vis spectrum of $\mathbf{7^{Me}}$ in THF at various concentrations at room temperature.

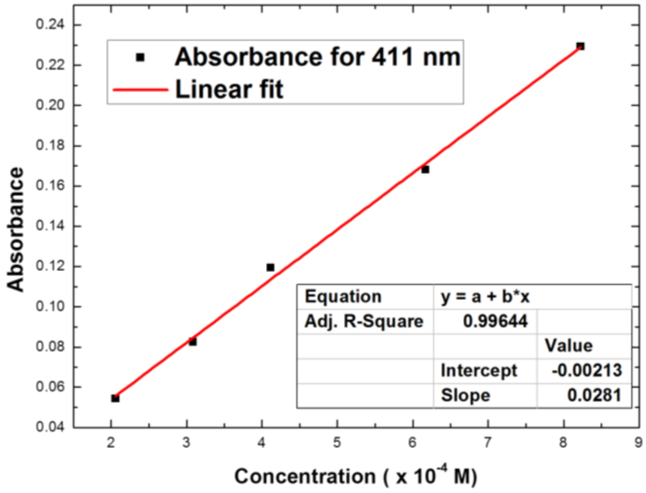

Figure S62. Linear regression of $\mathbf{7^{Me}}$ at 411 nm.

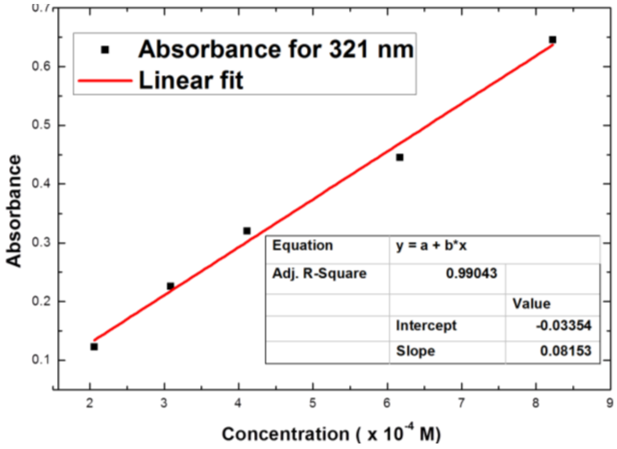

Figure S63. Linear regression of **7^{Me}** at 321 nm.

Crystallographic Details

Single-crystal X-ray diffraction data of $\mathbf{2^{H}}$, $\mathbf{2^{Me}}$, $\mathbf{3^{H}}$, $\mathbf{3^{Me}}$, $\mathbf{6^{H}}$, $\mathbf{6^{Me}}$, $\mathbf{7^{H}}$, and $\mathbf{7^{Me}}$ were collected using a Rigaku diffractometer with graphite-monochromated molybdenum $K\alpha$ radiation, λ = 0.71073 Å. Data integration and reduction were processed with CrysAlisPro software.[S22] An empirical absorption correction was applied to the collected reflections with SCALE3 ABSPACK integrated with CrysAlisPro. The structures were solved by direct methods using SHELXT[S23] program and refined by full matrix least-squares method based on F^2 by using SHELXL[S24] program through Olex2[S25] interface. All non-hydrogen-atoms were refined with anisotropic displacement parameters. The hydrogen atoms were refined isotropically on calculated positions using a riding model with their U_{iso} values constrained to 1.5 U_{eq} of their pivot atoms for terminal sp^3 carbon atoms and 1.2 times for the aromatic carbon atoms. Crystal data and structure refinement of all these compounds are summarized in Tables S5-S12.

Table S5: Crystal data and structure refinement for **2^{H}** (CCDC 1902844)

Identification code	AJ0237	
Empirical formula	$C_{22}H_{28}N_2$	
Formula weight	320.46	
Temperature	276.46(10)	
Wavelength	0.71073 Å	
Crystal system	orthorhombic	
Space group	$Pna2_1$	
Unit cell dimensions	a = 12.6922(5) Å	α = 90°
	b = 7.1119(4) Å	β = 90°
	c = 21.6435(9) Å	γ = 90°
Volume	1953.67(16) $Å^3$	
Z	4	
Density (calculated)	1.090 g/cm^3	
Absorption coefficient	0.063 mm^{-1}	
F(000)	696.0	
Crystal size	0.534 x 0413 x 0.236 mm^3	
Theta range for data collection	3.015 to 26.497	
Index ranges	$-15 \le h \le 15$, $-8 \le k \le 7$, $-27 \le l \le 26$	
Reflections collected	21099	
Independent reflections	3947 [Rint = 0.0417, Rsigma = 0.0385]	
Completeness to theta = 26.497°	100%	
Absorption correction	MULTI-SCAN	
Max. and min. transmission	0.664 and 1.000	
Refinement method	Full-matrix least-squares on F2	
Data / restraints / parameters	3947/1/221	
Goodness-of-fit on F^2	1.042	
Final R indices [I>2sigma(I)]	R1 = 0.0635, wR2 = 0.1572	
R indices (all data)	R1 = 0.0820, wR2 = 0.1758	
Extinction coefficient	N/A	
Largest diff. peak and hole	0.34/-0.22 e.$Å^{-3}$	

Table S6: Crystal data and structure refinement for **2^Me** (CCDC 1902845)

Identification code	AJ0315	
Empirical formula	$C_{24}H_{32}N_2$	
Formula weight	348.51	
Temperature	120.00(10)	
Wavelength	0.71073Å	
Crystal system	monoclinic	
Space group	$P2_1/n$	
Unit cell dimensions	a = 6.5653(5)Å	α = 90°
	b = 20.8366(16)Å	β = 96.484(8)°
	c = 7.6807(6) Å	γ = 90°
Volume	1043.99(14)Å^3	
Z	2	
Density (calculated)	1.109g/cm^3	
Absorption coefficient		
F(000)	380.0	
Crystal size	0.15 x 0.12 x 0.11 mm^3	
Theta range for data collection	2.842 to 28.894	
Index ranges	-7 ≤ h ≤ 8, -27 ≤ k ≤ 26, -8 ≤ l ≤ 9	
Reflections collected	7709	
Independent reflections	2406 [Rint = 0.0438, Rsigma = 0.0539]	
Completeness to theta = 28.894°	100%	
Absorption correction	MULTI-SCAN	
Max. and min. transmission	0.433 and 1.000	
Refinement method	Full-matrix least-squares on F2	
Data / restraints / parameters	2406/0/122	
Goodness-of-fit on F^2	1.084	
Final R indices [I>2sigma(I)]	R_1 = 0.0690, wR_2 = 0.1704	
R indices (all data)	R_1 = 0.0946, wR_2 = 0.1827	
Extinction coefficient	N/A	
Largest diff. peak and hole	0.33/-0.29 e.Å^{-3}	

Table S7: Crystal data and structure refinement for **3^H** (CCDC 1902846)

Identification code	AJ0253	
Empirical formula	$C_{16}H_{21}F_3NO_3S$	
Formula weight	364.40	
Temperature	293(2)	
Wavelength	0.7103 Å	
Crystal system	monoclinic	
Space group	*C*2/*c*	
Unit cell dimensions	a= 11.9074(10) Å	α = 90°
	b = 17.5699(11)Å	β = 90.910(7)°
	c = 17.2457(11)Å	γ = 90°
Volume	3607.5(4)$Å^3$	
Z	8	
Density (calculated)	1.342g/cm^3	
Absorption coefficient	0.222 mm^{-1}	
F(000)	1528.0	
Crystal size	0.363 x 0.259 x 0.133 mm^3	
Theta range for data collection	2.602 to 26.499	
Index ranges	-14 ≤ h ≤ 14, -18 ≤ k ≤ 21, -20 ≤ l ≤ 21	
Reflections collected	13790	
Independent reflections	3721 [R_{int} = 0.0354, R_{sigma} = 0.0368]	
Completeness to theta = 26.499°	100%	
Absorption correction	MULTI-SCAN	
Max. and min. transmission	0.663 and 1.000	
Refinement method	Full-matrix least-squares on F2	
Data / restraints / parameters	3721/0/221	
Goodness-of-fit on F^2	1.097	
Final R indices [I>2sigma(I)]	R_1 = 0.0728, wR_2 = 0.1615	
R indices (all data)	R_1 = 0.0862, wR_2 = 0.1671	
Extinction coefficient	N/A	
Largest diff. peak and hole	0.71/-0.50 e.$Å^{-3}$	

Table S8: Crystal data and structure refinement for **3^{Me}** (CCDC 1902848)

Identification code	AJ0325	
Empirical formula	$C_{17}H_{23}F_3NO_3S$	
Formula weight	378.42	
Temperature	119.64(10)	
Wavelength	0.7103 Å	
Crystal system	monoclinic	
Space group	$P2_1/c$	
Unit cell dimensions	a = 12.0989Å	α = 90°
	b = 14.3607Å	β = 115.785(6)°
	c = 11.8214Å	γ = 90°
Volume	1849.45(16) Å^3	
Z	4	
Density (calculated)	1.359g/cm^3	
Absorption coefficient	0.219 mm^{-1}	
F(000)	796.0	
Crystal size	0.7 x 0.274 x 0.268 mm^3	
Theta range for data collection	3.398 to 26.5	
Index ranges	-12 ≤ h ≤ 15, -15 ≤ k ≤ 18, -14 ≤ l ≤ 14	
Reflections collected	13967	
Independent reflections	3794 [Rint = 0.0258, Rsigma = 0.0259]	
Completeness to theta = 26.5°	100%	
Absorption correction	MULTI-SCAN	
Max. and min. transmission	0.583 and 1.000	
Refinement method	Full-matrix least-squares on F2	
Data / restraints / parameters	3794/0/231	
Goodness-of-fit on F^2	1.069	
Final R indices [I>2sigma(I)]	R_1 = 0.0375, wR_2 = 0.0952	
R indices (all data)	R1 = 0.0439, wR2 = 0.0983	
Extinction coefficient	N/A	
Largest diff. peak and hole	0.35/-0.46 e.Å^{-3}	

Table S9: Crystal data and structure refinement for **6^{H}** (CCDC 1902849)

Identification code	AJ0367	
Empirical formula	$C_{15}H_{21}N$	
Formula weight	215.33	
Temperature	120.15	
Wavelength	0.71073	
Crystal system	monoclinic	
Space group	$P2_1/c$	
Unit cell dimensions	a= 11.8621(6) Å	α = 90°
	b = 12.1669(6) Å	β = 107.166(5)°
	c = 9.3004(5) Å	γ = 90°
Volume	1282.49(12)Å^3	
Z	4	
Density (calculated)	1.115g/cm^3	
Absorption coefficient	MULTI-SCAN	
F(000)	472.0	
Crystal size	0.607 x 0.385 x 0.295 mm^3	
Theta range for data collection	2.839 to 28.894	
Index ranges	-15 ≤ h ≤ 16, -12 ≤ k ≤ 16, -12 ≤ l ≤ 11	
Reflections collected	17952	
Independent reflections	3129 [R_{int} = 0.0356, R_{sigma} = 0.0285]	
Completeness to theta =28.894°	100%	
Absorption correction	MULTI-SCAN	
Max. and min. transmission	0.364 and 1.000	
Refinement method	Full-matrix least-squares on F2	
Data / restraints / parameters	3129/0/149	
Goodness-of-fit on F^2	1.076	
Final R indices [I>2sigma(I)]	R_1 = 0.0462, wR_2 = 0.1147	
R indices (all data)	R_1 = 0.0585, wR_2 = 0.1212	
Extinction coefficient	N/A	
Largest diff. peak and hole	0.31/-0.22 e.Å^{-3}	

Table S10: Crystal data and structure refinement for **6^{Me}** (CCDC 1902850)

Identification code	AJ0372	
Empirical formula	$C_{16}H_{23}N$	
Formula weight	229.35	
Temperature	120.00(10)	
Wavelength	0.7103 Å	
Crystal system	monoclinic	
Space group	$P2_1/n$	
Unit cell dimensions	a= 6.2169(5)Å	α = 90°
	b = 19.8523(12)Å	β = 94.099(6)°
	c = 11.1176(7)Å	γ = 90°
Volume	1368.62(16)Å^3	
Z	4	
Density (calculated)	1.113g/cm^3	
Absorption coefficient	0.064 mm^{-1}	
F(000)	504.0	
Crystal size	0.198 x 0.106 x 0.059 mm^3	
Theta range for data collection	2.754 to 28.653	
Index ranges	-8 ≤ h ≤ 6, -23 ≤ k ≤ 26, -13 ≤ l ≤ 14	
Reflections collected	10891	
Independent reflections	3178 [Rint = 0.0398, Rsigma = 0.0460]	
Completeness to theta = 28.653°	100%	
Absorption correction	MULTI-SCAN	
Max. and min. transmission	0.454 and 1.000	
Refinement method	Full-matrix least-squares on F2	
Data / restraints / parameters	3178/0/159	
Goodness-of-fit on F^2	1.049	
Final R indices [I>2sigma(I)]	R_1 = 0.0481, wR_2 = 0.1179	
R indices (all data)	R_1 = 0.0652, wR_2 = 0.1252	
Extinction coefficient	N/A	
Largest diff. peak and hole	0.28/-0.20 e.Å^{-3}	

Table S11: Crystal data and structure refinement for **7^H** (CCDC 1902852)

Identification code	AJ0562	
Empirical formula	$C_{31}H_{42}N_2O_3F_3S$	
Formula weight	579.72	
Temperature	120.02(10)	
Wavelength	0.7103 Å	
Crystal system	monoclinic	
Space group	*Cc*	
Unit cell dimensions	a = 20.953(5) Å	α = 90°
	b = 10.3842(10) Å	β = 131.68(4)°
	c = 18.453(4) Å	γ = 90°
Volume	2998.7(17) $Å^3$	
Z	4	
Density (calculated)	1.284g/cm^3	
Absorption coefficient	0.161 mm^{-1}	
F(000)	1236.0	
Crystal size	0.14 x 0.13 x 0.12 mm^3	
Theta range for data collection	2.956 to 29.05	
Index ranges	$-28 \le h \le 27$, $-14 \le k \le 11$, $-20 \le l \le 24$	
Reflections collected	16775	
Independent reflections	6048 [R_{int} = 0.0719, R_{sigma} = 0.0996]	
Completeness to theta = 29.05°	100%	
Absorption correction	MULTI-SCAN	
Max. and min. transmission	0.296 and 1.000	
Refinement method	Full-matrix least-squares on F2	
Data / restraints / parameters	6048/421/473	
Goodness-of-fit on F^2	1.032	
Final R indices [I>2sigma(I)]	R_1 = 0.0719, wR_2 = 0.1435	
R indices (all data)	R_1 = 0.1329, wR_2 = 0.1684	
Extinction coefficient	N/A	
Largest diff. peak and hole	0.36/-0.24 e.$Å^{-3}$	

Table S12: Crystal data and structure refinement for **7^{Me}** (CCDC 1902853)

Identification code	AJ0454	
Empirical formula	$C_{33}H_{46}F_3N_2O_3S$	
Formula weight	607.78	
Temperature	120.15	
Wavelength	0.7103	
Crystal system	monoclinic	
Space group	*C2/c*	
Unit cell dimensions	a= 21.170(4)Å	α = 90°
	b = 11.589(2) Å	β = 125.81(3)°
	c = 16.143(3) Å	γ = 90°
Volume	3211.8(12) $Å^3$	
Z	4	
Density (calculated)	1.257g/cm^3	
Absorption coefficient	0.153 mm^{-1}	
F(000)	1300.0	
Crystal size	0.14 x 0.12 x 0.1 mm^3	
Theta range for data collection	3.013 to 28.932	
Index ranges	$-25 \le h \le 28$, $-15 \le k \le 15$, $-21 \le l \le 18$	
Reflections collected	24016	
Independent reflections	3886 [R_{int} = 0.0423, R_{sigma} = 0.0288]	
Completeness to theta = 28.932°	100.0%	
Absorption correction	MULTI-SCAN	
Max. and min. transmission	0.802 and 1.000	
Refinement method	Full-matrix least-squares on F2	
Data / restraints / parameters	3886/18/219	
Goodness-of-fit on F^2	1.058	
Final R indices [I>2sigma(I)]	R_1 = 0.0675, wR_2 = 0.1783	
R indices (all data)	R_1 = 0.0817, wR_2 = 0.1869	
Extinction coefficient	N/A	
Largest diff. peak and hole	0.72/-0.70 e.$Å^{-3}$	

References

[S1] a) R. S. Grainger, A. Patel, *Chem. Commun*. **2003**, 1072–1073; b) B. R. Dohner, W. H. Saunders Jr., *J. Am. Chem. Soc*. **1986**, *108*, 245–247.

[S2] S. Stoll, A. Schweiger, *J. Magn. Reson.* **2006**, *178*, 42–55.

[S3] F. Neese, *WIREs Comput Mol Sci* **2018**, *8*, 1327–1332.

[S4] C. Adamo, V. Barone, *J. Chem. Phys.* **1999**, *110*, 6158.

[S5] F. Weigend, R. Ahlrichs, *Phys. Chem. Chem. Phys.* **2005**, *7*, 3297.

[S6] S. Grimme, *J. Comput. Chem.* **2006**, *27*, 1787;

[S7] S. Grimme, *J. Comput. Chem.* **2004**, *25*, 1463–1473.

[S8] S. Grimme, J. Antony, S. Ehrlich, H. Krieg, *J. Chem. Phys.* **2010**, *132*, 154104.

[S9] S. Grimme, S. Ehrlich, L. Goerigk, *J. Comput. Chem.* **2011**, *32*, 1456–1465.

[S10] T. Petrenko, S. Kossmann, F. Neese, *J. Chem. Phys.* **2011**, *134*, 54116.

[S11] F. Neese, G. Olbrich, *Chem. Phys. Lett.* **2002**, *362*, 170.

[S12] R. Izsák, F. Neese, *J. Chem. Phys.* **2011**, *135*, 144105.

[S13] J. L. Whitten, *J. Chem. Phys.* **1973**, *58*, 4496.

[S14] O. Vahtras, J. Almlöf, M. W. Feyereisen, *Chem. Phys. Lett.* **1993**, *213*, 514.

[S15] F. Neese, F. Wennmohs, H. Hansen, U. Becker, *Chem. Phys.* **2009**, *356*, 98.

[S16] F. Neese, *J. Comput. Chem.* **2003**, *24*, 1740.

[S17] K. Eichkorn, O. Treutler, H. Öhm, M. Häser, R. Ahlrichs, *Chem. Phys. Lett.* **1995**, *242*, 652–660.

[S18] K. Eichkorn, F. Weigend, O. Treutler, R. Ahlrichs, *Theor. Chem. Acc.* **1997**, *97*, 119.

[S19] V. Barone, M. Cossi, *J. Phys. Chem. A* **1998**, *102*, 1995.

[S20] M. D. Hanwell, D. E. Curtis, D. C. Lonie, T. Vandermeersch, E. Zurek, G. R. Hutchison, *J. Cheminform.* **2012**, *4*, 17.

[S21] *Avogadro: an open-source molecular builder and visualization tool. Version 1.2.0. modified version with extended ORCA support http://avogadro.openmolecules.net/ and https://orcaforum.cec.mpg.de/*.

[S22] CrysAlisPro: Rigaku Oxford Diffraction (**1995-2017**). Oxford Diffraction Ltd, Abingdon, Oxfordshire, England.

[S23] G. M. Sheldrick, *Acta Cryst*. **2015**, *A71*, 3–8.

[S24] G. M. Sheldrick, *Acta Cryst*. **2015**, *C71*, 3–8.

[S25] O. V. Dolomanov, L. J. Bourhis, R. J. Gildea, J. A. K. Howard, H. Puschmann, *J. Appl. Crystallogr*. **2009**, *42*, 339–341.
